# Highly Efficient Mechanochromic Thermally Activated Delayed Fluorescence in the Deep Red to Near‐Infrared in Copper(I) [2.2]Isoindolinophanyl‐Carbene Carbazolates

**DOI:** 10.1002/anie.5338298

**Published:** 2026-04-25

**Authors:** André M. T. Muthig, Sabyasachi Maity, Andreas Prüfer, Justin Krieger, Andrei Belyaev, Jan‐Benedikt Weiß, Sebastian Henke, Benjamin Hupp, Björn Ewald, Jens Pflaum, Andreas Steffen

**Affiliations:** ^1^ Department of Chemistry and Chemical Biology TU Dortmund University Dortmund Germany; ^2^ Experimental Physics VI Julius‐Maximilians University Würzburg Würzburg Germany

**Keywords:** carbenes, copper, mechanochromism, Near‐IR, TADF

## Abstract

Low energy triplet emitters are highly relevant for the development of OLEDs and fiber optics‐based IT applications, but typically suffer from nonradiative decay due to the energy gap law (EGL). Excited state deactivation can be limited by enhancing the radiative decay rate via thermally activated delayed fluorescence (TADF), bypassing spin‐forbidden phosphorescence. We report on linear copper(I) complexes bearing a recently reported [2.2]isoindolinophanyl‐carbene (iPC) ligand as potent excited state π‐acceptor. The compounds show efficient TADF from ligand‐to‐ligand charge transfer (^1/3^LLCT) states with reverse intersystem‐crossing (RISC) of *k*_RISC_ = 0.6–21·10^9^ s^−1^, quantum yields of up to 0.8 and *k*_TADF_ of 0.8‐1.9·10^6^ s^−1^ that are among the fastest for Cu^I^ emitters, outcompeting traditional triplet emitters based on Ir^III^ and Pt^II^ as well as organic deep red to near‐IR TADF emitters. While yellow to red emission is observed in single crystals, embedding the complexes into polymers or grinding shifts the luminescence into the deep red to near‐IR. The mechanochromism is due to disruption of C─H⋯π interactions between the ligands, reducing the energy gap between the ground state and ^1/3^LLCT states. The Cu^I^ iPC complexes bear potential for devices operating under electroluminescent conditions as demonstrated by a proof‐of‐concept deep‐red OLED application.

## Graphical abstract

Electrophilic [2.2]isoindolinophanyl‐based carbene iPC has been employed as charge‐transfer acceptor ligand in a series of [Cu(iPC)(carbazolate)], which show mechanochromism and environment‐dependent luminescence due to intermolecular C─‐H‐π interactions. The complexes exhibit efficient red to NIR TADF with quantum yields of 0.11–0.80 and exceptional radiative rate constants k_r_ of up to 1.9·10^6^ s^−1^, and are among the fastest TADF emitters to date.

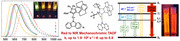

## 1 Introduction

Molecular triplet emitters in the deep red to NIR are of great interest as active components in ultra HD night vision and OLED displays as well as in room‐temperature single photon sources for tap‐proof quantum communication [1, 2, 3, 4, 5, 6, 7]. However, such low energy emission from longer‐lived triplet excited states is typically prone to facile nonradiative decay and small luminescence quantum yields *Φ* due to the energy‐gap law (EGL). The EGL dictates that small energy differences between the excited state and the ground state lead to large vibrational wavefunction overlap with concomitant high nonradiative decay rates *k_nr_* and generally lower radiative rate constants *k_r_* compared to high‐energy emission [8, 9, 10]. This effect is even observed for transition metal complexes of rare and heavy 5d elements, such as Os^II^, Ir^III^, and Pt^II^, that usually mediate strong spin‐orbit coupling (SOC) for fast phosphorescence with high $\;k_{r}^{T1}$ in the visible region of the electromagnetic spectrum [4, 6, 11].

An alternative possibility to reduce the limitation of the EGL is to enhance radiative decay of triplet excitons indirectly via thermally activated delayed fluorescence (TADF) [12, 13]. This process bypasses spin‐forbidden phosphorescence T_1_→S_0_ by endothermic reverse intersystem‐crossing (RISC) and subsequent spin‐allowed fluorescence S_1_→S_0_ to exploit both types of excitons formed under the operating conditions in electrically driven devices [14, 15, 16, 17]. The critical factors for efficient TADF are thus i) a small energy gap *ΔE*(S_1_–T_1_), which determines the rate constant *k_RISC_* and thus the steady‐state equilibrium T_1_ ⇄ S_1_, ii) a high oscillator strength *f*(S_1_→S_0_) giving a high rate constant $k_{r}^{S1}$ for fluorescence, and iii) sufficient SOC for efficient state mixing. Understanding the molecular structural requirements to design the excited states to meet all criteria is key to achieve emitters with a resulting overall radiative rate constant *k*_TADF_ to outcompete the EGL at room temperature for exploiting singlet and triplet excitons alike.

**FIGURE 1:**
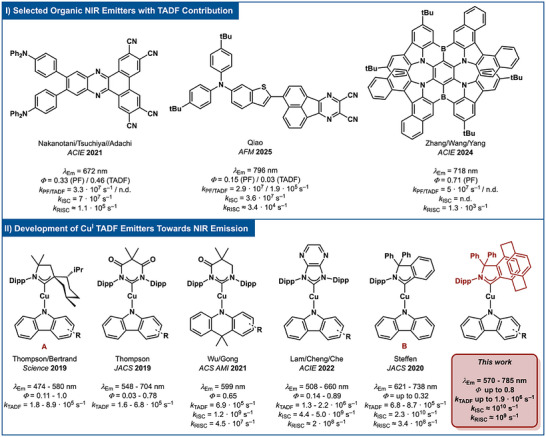
Selected examples of I) reported organic NIR emitters with dual fluorescence and TADF contribution [25, 27, 28], and II) Cu^I^‐based pure TADF emitters with various electrophilic carbenes as π‐chromophores leading to high *k*_TADF_ for emission involving triplet excited states [15, 22, 24, 31, 32].

A typical design strategy for TADF emitters involves a donor(D)–acceptor(A) motif, where the charge‐transfer (CT) ensures very small and favorable *ΔE*(S_1_–T_1_). However, maintaining high $k_{r}^{S1}$ can become challenging as ^1^CT states often exhibit small orbital overlap between the D/A sites, reducing the oscillator strength *f*. Organic TADF emitters usually benefit from high $k_{r}^{S1}$ of up to 10^8^ s^−1^, while ISC is comparably slow due to inefficient SOC (typically *k_ISC_* < 10^7^ s^−1^) (Figure 1). Thus, for most cases, the dominant photoluminescence mechanism is prompt fluorescence. The RISC process can still occur on the timescale of 10^5^–10^6^ s^−1^, and for a few outstanding examples of through‐space CT TADF emitters *k_RISC_* has been found to be ca. 10^7^ s^−1^, albeit at the expense of $k_{r}^{S1}$ of only 10^6^ s^−1^ [18, 19]. Consequently, the RISC allows for TADF to be involved as an additional radiative path in the electroluminescent device. However, it is important to note that the resulting *k*_TADF_ for triplet exciton harvesting via delayed fluorescence in such organic systems, which must not be confused with the much higher values of $k_{r}^{S1}$ often reported, does not exceed 10^5^ s^−1^. This would lead to relatively low brightness in terms of photons/second in the deep red to near IR as required for many applications as well as to stability issues. An alternative is to increase the SOC via heavy metals to accelerate the spin‐forbidden processes of the TADF mechanism [12].

Employing this strategy, the highest *k*_TADF_ for TADF to date of up to 4·10^6^ s^−1^ have been achieved for d^10^ coinage metal complexes based on precious Ag and Au complexes, where various carbenes serve as acceptor moieties in combination with electron‐rich anionic π‐donor ligands to form ligand‐to‐ligand ^1/3^(LL)CT states [12, 20, 21]. Noteworthy, these classes of emitters by far outcompete the most efficient phosphorescent 5d element complexes. However, structurally related systems based on earth abundant Cu^I^ appear to be less efficient with *k*_TADF_ of 10^5^–10^6^ s^−1^ [12, 14, 22, 23], although Che et al. demonstrated that *k*_TADF_ up to 2.2·10^6^ s^−1^ can be achieved using pyrazine‐fused carbene ligands due to a small *ΔE*(S_1_–T_1_) of ca. 400–500 cm^−1^ in conjunction with a high $k_{r}^{S1}$ of ca. 3·10^7^ s^−1^ [24].

Interestingly, efficient TADF with high *k*_TADF_ > 10^5^ s^−1^ suitable for device and IT technologies in the near‐IR is very rare, even for purely organic NIR emitters that are mostly fluorescent with some TADF contribution [25, 26, 27, 28], and requires energetic adjustment of the frontier orbital region without sacrificing the parameters relevant for *k*_TADF_ (Figure 1) [6, 7, 25, 26, 27, 28, 29, 30]. For example, we have shown that extending the conjugation of the acceptor ligand´s π system in TADF‐active [Cu(Cz)(cAAC)] (**A**) (cAAC = cyclic amino(alkyl) carbene, Cz = carbazolate) by exchange of the carbene for cyclic amino(aryl) carbene (cAArC) in **B** reduces the LUMO energy and shifts *λ*_em_ bathochromically from 470 and 492 nm in polystyrene and 2‐MeTHF, respectively, into the deep‐red to near‐IR (solid/PMMA/THF: 621/638/738 nm) (Figure 1) [22]. In addition, this modification greatly enhanced *k*_TADF_ by a factor of 3 to ca. 9·10^5^ s^−1^. The latter effect is the result of a shorter radiative lifetime *τ*(S_1_) of 17 ns compared to 150 ns for **A** as determined by single molecule spectroscopy, in combination with a reduced *ΔE*(S_1_–T_1_) of only 532 cm^−1^ leading to fast RISC. Such low energy emission in nonsolvated linear coordinated coinage metal Cz complexes has only been achieved with a conjugated diamido carbene (DAC) as acceptor ligand, giving *λ*_em_ = 704 and 658 nm in polystyrene (PS) and the solid, but to the expense of low *k*_TADF_ of 1.6 and 3.1·10^5^ s^−1^, respectively [31]. Another strategy to achieve near‐IR TADF harvesting triplet states is based on aggregation of (metal)organic dyes, although similarly small *k*_TADF_ << 10^6^ s^−1^ are found and aggregates are limited with regard to applications [27, 28, 29].

Considering that linearly coordinated coinage metal complexes are the most efficient class of TADF emitters in terms of the radiative rate constants, we are interested in the molecular design of the acceptor carbene moieties in such structures to modulate the excited state properties and to achieve efficient TADF in the deep red to NIR. We have recently reported the synthesis, electronic, and photophysical properties of a sterically demanding [2.2]isoindolinophanyl‐based carbene (iPC) bearing a paracyclophane moiety, which is a further extension of the cAArC ligand to provide through‐space conjugation [33]. In contrast to structurally related [Cu(cAAC)_2_]PF_6_ emitting in the UV‐blue with short radiative *τ* = 9 µs [34], ultralong‐lived phosphorescence of 185 µs in the green‐yellow region of the electromagnetic spectrum from triplet intraligand (^3^IL)CT states in [Au(iPC)_2_]OTf in solution was observed, which has been exploited in energy transfer photocatalysis [33]. We anticipated that the unusual electronic structure of the iPC ligand with its extended *π* conjugation may influence the TADF behavior in Cu^I^ Cz complexes, which we report herein and found that very high *k*_TADF_ of up to 1.9·10^6^ s^−1^ and highly efficient deep red to NIR emission can be achieved, serving as a new design motif (Figure 1).

## 2 Results and Discussion

### 2.1 Synthesis and Structural Characterization

**FIGURE 2:**
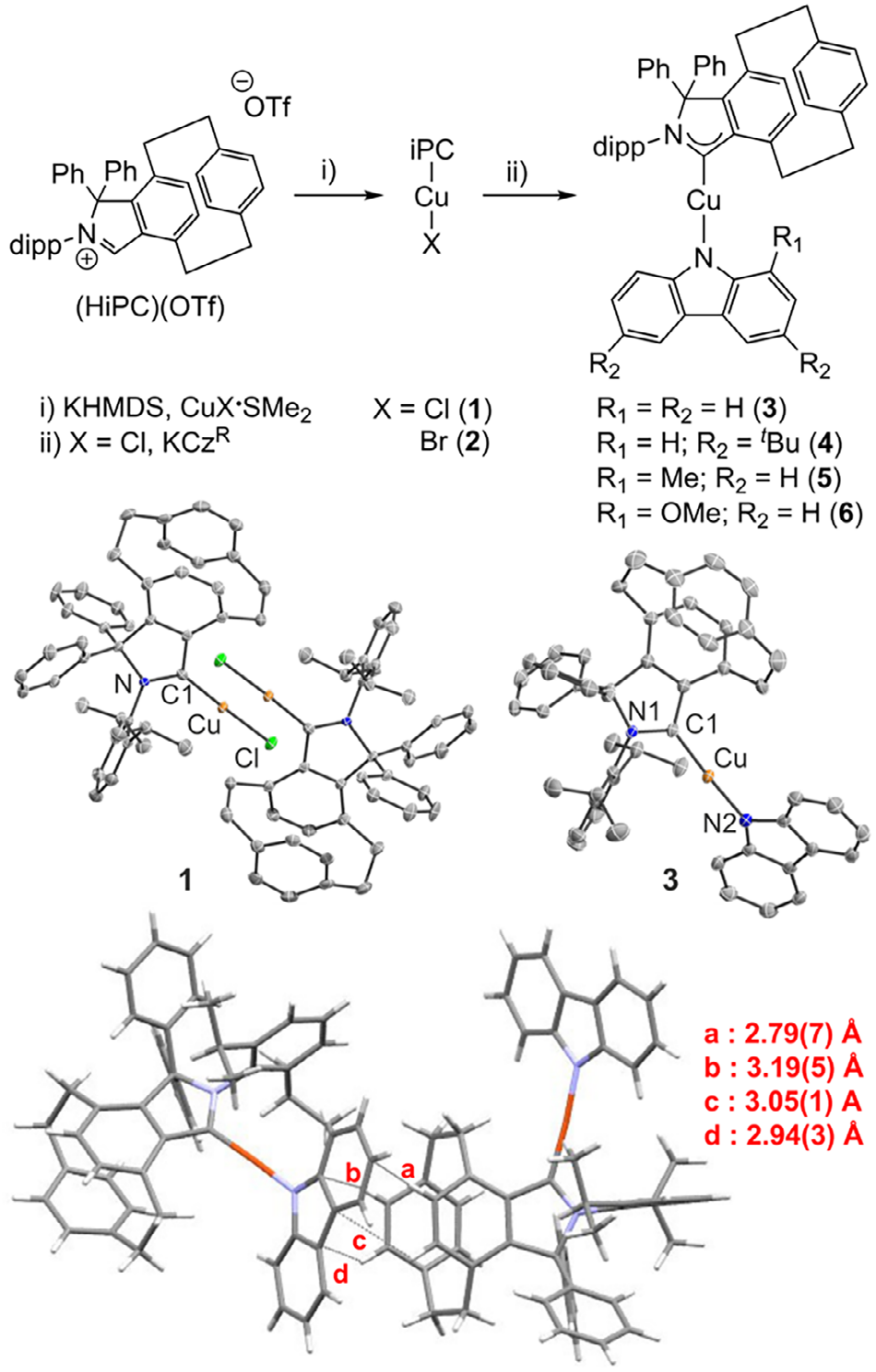
Top: Synthesis of the Cu^I^ iPC complexes **1–6**. Middle: Molecular structures of **1** and **3** in the solid state determined by SC‐XRD (C1─Cu─L (°): 174.77(10) (**1**), and 175.10(9) (**3**); dihedral angle between the planes of the Cz and the iPC ligand: 57° (**3**). Bottom: C─H⋯π interactions between the Cz and iPC ligands in **3**.

The synthons [CuX(iPC)] (X = Cl (**1**), Br (**2**)) were prepared by in situ deprotonation of (HiPC)OTf with potassium hexamethyldisilazane (KHMDS) at ^−^85°C in the presence of CuX·SMe_2_ (Figure 2). Diffusion of pentane into saturated THF solutions of **1** or **2** gave orange, weakly red phosphorescent single crystals (Figures S27, S29, S30 and Table S4), of which X‐ray diffraction studies (SC‐XRD) confirmed the linear coordination and revealed weak intermolecular side‐on interactions to form a Cu_2_X_2_ square in the solid state (Figure S18). Addition of the respective potassium carbazolates to a solution of **2** in THF at RT led to the formation of the linear copper(I) complexes **3–6**, and single crystals could be obtained in excellent yields of 74%–79% for structural characterization and photophysical studies (Figure 2). Linear d^10^ carbene coinage metal carbazolate complexes often adopt coplanar arrangements [12, 15, 17, 20, 21, 22, 23, 35], but the steric demand of the iPC ligand in **3–6** leads to dihedral angles between 31°–84° in the crystalline solid state, with the largest distortion observed for [Cu(Cz^Me^)(iPC)] (**5**) (Figures S19–S22). Importantly, the paracyclophane moiety of the iPC ligand undergoes strong C─H⋯π interactions with the Cz ligand of the neighboring complex molecules in **3–6** (Figures 2, S23–S26).

### 2.2 Photophysical and Mechanochromic Properties

**FIGURE 3:**
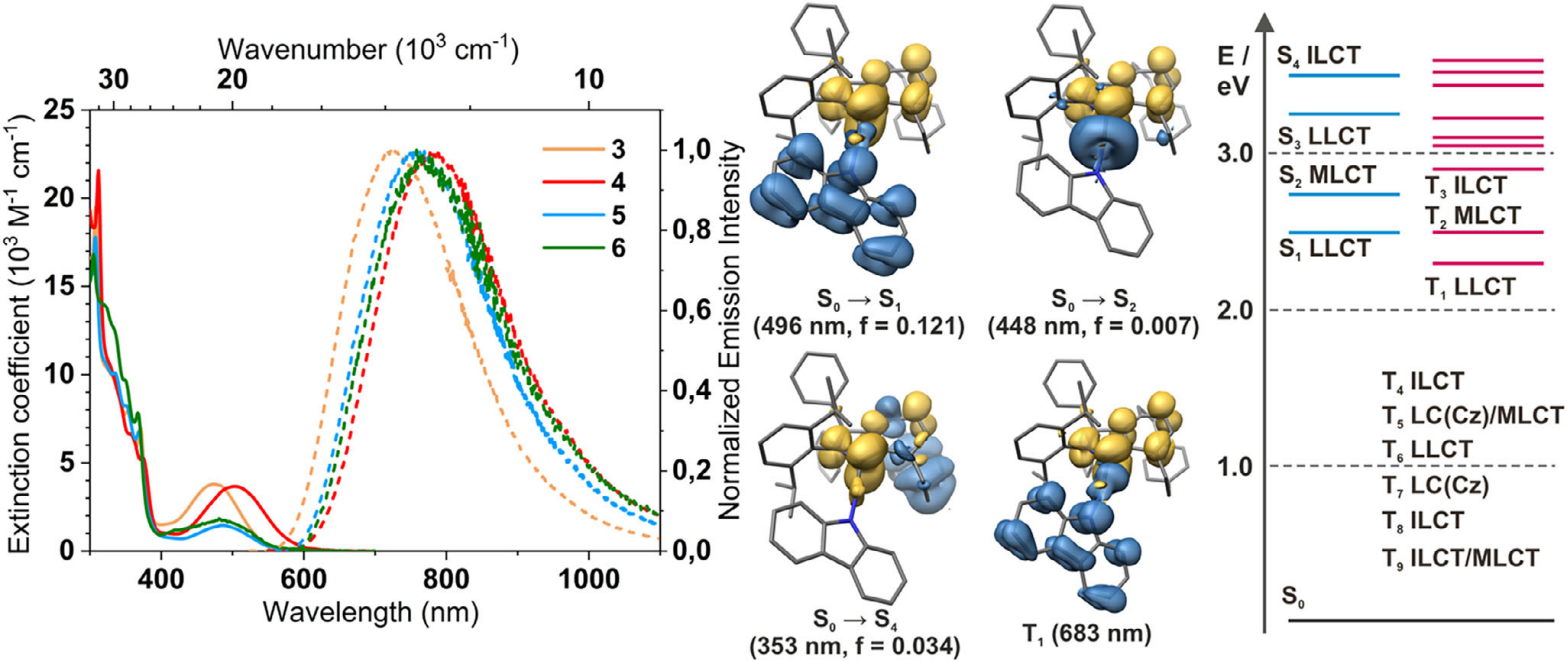
Left: Experimental UV–vis absorption (solid) and luminescence spectra (dashed) of **3–6** in THF solution. Middle: Electron density differences (EDD) of selected vertical transitions from the ground state S_0_ of [Cu(Cz)(iPC)] (**3**), and of the geometry optimized T_1_ state (calculated at the D3BJ‐PBE0/ZORA/def2‐SVP level of theory; loss of electron density is indicated in blue and gain in yellow; isosurface values ±0.001; see also ESI). Right: Franck‐Condon diagram based on the TD‐DFT calculations for **3**.

The UV–vis absorption spectra of **3–6** in THF are similar to previously reported [Cu(Cz)(cAArC)] (**B**) [22] and display high energy absorptions beyond 300 nm with *ε* > 20.000 M^−1 ^cm^−1^ that can be assigned to LC(π‐π*) transitions of the aromatic substituents (Figure 3). A broad IL(iPC)CT transition between 300 and 340 nm (*ε* = ca. 10–14·10^3^ M^−1^ cm^−1^) overlaps with a vibrationally resolved band between 340 and 375 nm (*ε* = 7–10·10^3^ M^−1^ cm^−1^), which we deduce from comparison with [Au(iPC)_2_]OTf and [RhCl(CO)_2_(iPC)] recently reported by us [33], and from our TD‐DFT calculations. In addition, a broad low energy ML/LLCT band centered at *λ*_abs_ = 475 nm (*ε* = 4,000 M^−1^ cm^−1^) is observed for **3**, which is bathochromically shifted upon Cz substitution with the most pronounced effect for the *t*Bu derivative in **4** (*λ*_abs_ = 510 nm). Noteworthy, the absorptivity of this transition in **5** and **6** is reduced by a factor of 2 because substituents in the 1‐position of the Cz ligand favor noncoplanar conformations, reducing the oscillator strength of the LLCT contribution.

The iPC complexes **3–6** show broad near‐IR luminescence in THF solution with maxima between *λ*_em_ = 725–785 nm, which is prone to nonradiative decay via structural flexibility in solution and the EGL (Table 1, Figure 3). Photoluminescence quantum yields *Φ*_PL_ of 0.010, 0.002, and 0.003 could be determined for **3**–**5**, respectively, and an observed short lifetime of 21 ns provides a radiative rate constant of *k*_r_ = 4.8·10^5^ s^−1^ for **3**, indicating the involvement of spin‐forbidden processes. Although the iPC complexes are competitive to recently reported NIR‐emissive dicopper(I) compounds, which exhibit luminescence efficiencies of 0.02%–0.50% in solution and have been applied in light emitting electrochemical cells [30, 36], the signal‐to‐noise ratio precluded more detailed studies regarding the luminescence mechanism in solution.

**TABLE 1:** Selected photoluminescence data for the copper(I) iPC complexes **3–6** in various media recorded at 297 K under argon.

Cpd.	Medium	*λ*_em_ / nm	*τ* / ns^a^	*Φ*	*k*_r_ / 10^5^ s^−1^	*k*_nr_ / 10^5^ s^−1^	Cpd.	Medium	*λ*_em_ / nm	*τ* / ns^a^	*Φ*	*k*_r_ / 10^5^ s^−1^	*k*_nr_ / 10^5^ s^−1^
**3**	Microcrystalline	570	577	0.80	14	3.5	**4**	Microcrystalline	610	398	0.75	19	6.3
	Ground solid	665	231	0.22	9.5	34		Ground solid	700	112	0.11	9.9	35
	PMMA	595	722	0.58	8.0	5.8		PMMA	630	451	0.30	6.7	16
	PS	615	700	0.62	8.9	5.5		PS	650	390	0.34	8.7	17
	THF	725	21	0.01	4.8	475		THF	785	n.d.	0.002		
**5**	Microcrystalline	620	374	0.51	14	13	**6**	Microcrystalline	605	253	0.29	11	27
	Ground solid	665	175	0.15	8.6	49		Ground solid	665	41	0.03	7.2	233
	PMMA	610	565	0.33	5.8	12		PMMA	610	245	0.11	4.5	36
	THF	755	n.d.	0.003				THF	765	n.d.	n.d.		

^a^ The time‐resolved luminescence decays were of multiexponential nature and the respective lifetime components *τ*_i_ and normalized pre‐exponential factors B_i_ have been used to calculate amplitude‐weighted average lifetimes $\tau =\sum_{i}\left(\tau_{i}\cdot B_{i}\right)$(see ESI).

**FIGURE 4:**
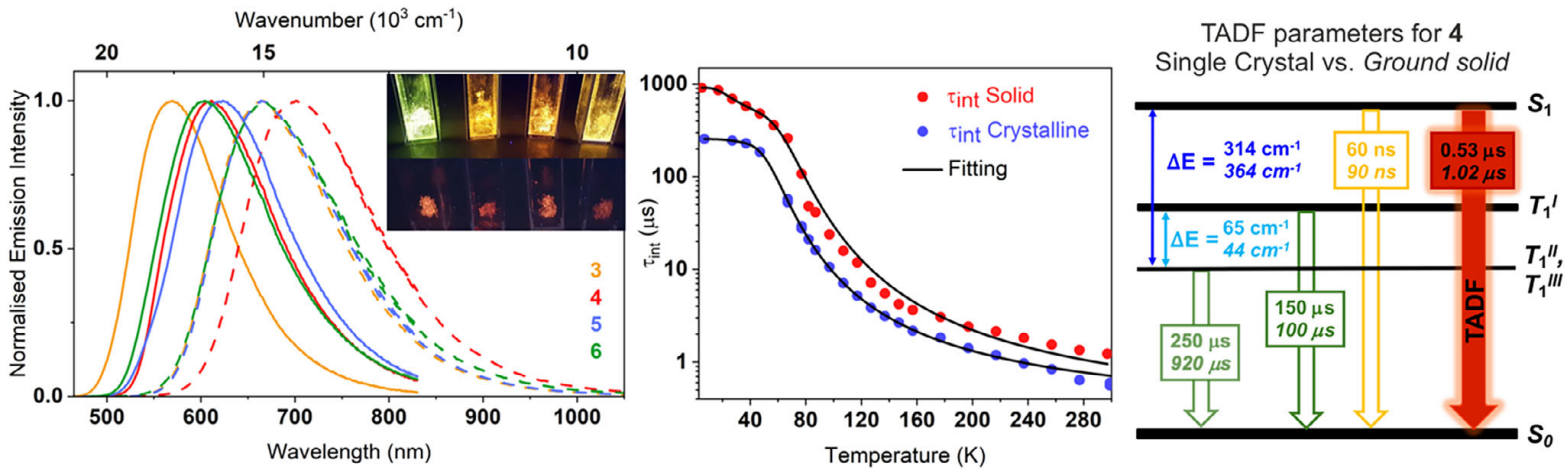
Left: emission spectra (*λ*_ex_ = 475 nm) and photographs under UV lamp irradiation of **3–6** in the microcrystalline solid state (solid) and upon grinding (dashed). Middle: temperature dependence of the radiative (intrinsic) lifetimes *τ*_int_ of crystalline [Cu(Cz^tBu^)(iPC)] (**4**) between 297 and 5 K. Right: Jablonski diagram for **4** with TADF relevant parameters obtained by fitting the VT time‐resolved data according to the equation given in the main text.

However, in the single crystalline solid state, very intense photoluminescence in the yellow to red region of the electromagnetic spectrum with high quantum yields *Φ*_PL_ of 0.29–0.80 at room temperature are observed (Figure 4 and Table 1). The broad spectral appearance suggests that the emission stems from LLCT/MLCT excited states, while the observed emission lifetimes *τ* of 250–560 ns are indicative for the presence of triplet states. Importantly, very high radiative rate constants of *k_r_* = 1.1–1.9·10^6^ s^−1^ are obtained, which are among the highest values observed so far for copper(I) complexes, outcompeting traditional Ir^III^‐ and Pt^II^‐based emitters [12, 15, 20, 23, 24, 31, 37, 38]. In line with the EGL, nonradiative decay becomes more efficient with increasing *λ*_em_ when comparing *k_nr_* of **3** and **4** (3.5 vs. 6.3·10^5^ s^−1^), and substitution in the 1‐position of the Cz ligand is disadvantageous as it further enhances *k_nr_* to 1.3 and 2.7·10^6^ s^−1^ for **5** and **6**, respectively, although *λ*_em_ is similar to **4** (Table 1).

High *k*_r_ are often associated with TADF due to small *ΔE*(S_1_–T_1_) leading to a steady‐state equilibrium between ^1/3^ML/LLCT states at room temperature by ISC/RISC processes, and our time‐resolved variable temperature (TR‐VT) luminescence measurements of the fastest emitter [Cu(Cz^tBu^)(iPC)] (**4**) confirm this assignment (Figure 4). Upon lowering the temperature to 5 K, the radiative intrinsic lifetime *τ*_int_ = 1/*k_r_* increases to 240 µs and the emission spectrum shows additional shoulders, which suggest that the ^1/3^LLCT states are thermally populated from an excited state with significant ^3^LC character with little operative SOC (Figure S35). The temperature dependence of *τ*_int_ can be fitted according to the following Equation 1 for a 4‐state‐scenario,

(1) $$\tau_{int}=\frac{1}{k_{r}}=\frac{2+e^{-\Delta E\left(T_{1}^{III}-T_{1}^{I,II}\right)/k_{B}T}+e^{-\Delta E\left(S_{1}-T_{1}^{I,II}\right)/k_{B}T}}{2\cdot k_{r}\left(T_{1}^{II,III}\right)+k_{r}\left(T_{1}^{I}\right)\cdot e^{-\Delta E\left(T_{1}^{III}-T_{1}^{I,II}\right)/k_{B}T}+k_{r}\left(S_{1}\right)\cdot e^{-\Delta E\left(S_{1}-T_{1}^{I,II}\right)/k_{B}T}}\;$$

giving *ΔE*(S_1_–T_1_) = 314 cm^−1^, which matches the difference of the emission onsets between 297 and 5 K, and a radiative lifetime 1/$k_{r}^{S1}$ = *τ*_F_ of 60 ns for the spin‐allowed S_1_→S_0_ transition.

Further insight into the excited state behavior is provided by employing the kinetic analysis of Dias, Penfold, and Monkman (DPM model, see also Supporting Information for details) [39]. Under the assumption of nonradiative decay occurring dominantly from S_1_ due to the spin‐allowed nature of this process, and thus quantitative RISC (*Φ*_RISC_ = 1), the following equations can be used:

(2) $$\Phi_{PF}=k_{r}^{S1}\;\cdot \tau_{PF}$$

(3) $$k_{ISC}=\frac{\Phi_{ISC}}{\tau_{PF}}=\frac{\Phi_{DF}}{\tau_{PF}\left(\Phi_{PF}+\Phi_{DF}\right)}\;$$

(4) $$k_{RISC}=\frac{k_{\mathrm{TADF}}}{\left(1-\Phi_{ISC}\right)}\;$$

(5) $$k_{nr}^{S1}=\frac{1}{\tau_{PF}}\;-\left(k_{r}^{S1}+k_{ISC}\right)$$

With *Φ*_PF_, *Φ*_ISC_, and *Φ*_DF_ being the quantum yields of prompt fluorescence, ISC and delayed fluorescence, respectively, and $k_{r}^{S1}$, *k*_TADF_ (which is *k*_r_ in Table 1), $k_{nr}^{S1}$, *k_ISC_*, and *k_RISC_* are the rate constants for fluorescence, delayed fluorescence, nonradiative decay from the S_1_ state, ISC and RISC, respectively, at room temperature. The temporal resolution of our spectrometer of 150 ps was not sufficient to resolve the residual prompt fluorescence S_1_→S_0_, and thus the timescale of *τ*_PF_ must be faster than this value. For structurally and electronically related [Cu(Cz)(cAArC)] (**B**), we found a value of ca. 42 ps and we thus suggest this as a lower limit for **4** as well, i.e. *τ*_PF_ = 150–42 ps [22]. Despite this ambiguity, minimum and maximum parameters related to the TADF mechanism can be deduced with $k_{r}^{S1}$ = 1.67 ·10^7^ s^−1^ obtained from the VT experiments (see above). Accordingly, we calculate *k_ISC_* (min/max) = 0.66/2.38·10^10^ s%^1^ and *k_RISC_* (min/max) = 0.06/2.1·10^10^ s%^1^, which gives an equilibrium constant of the excited state population *K*_eq_(T_1_ ⇄ S_1_) (min/max) = 0.087/0.883 at room temperature. We note that estimation of this equilibrium by purely energetic factors according to Equation 6 leads to a value of only 0.070, which is significantly more in favor of the T_1_ state. This discrepancy suggests that SOC effects by electronic and vibrational coupling with higher lying T_2_ or T_3_ states of ^3^MLCT or ^3^ILCT nature may be involved in the spin‐forbidden processes and influence RISC to a larger extent than ISC as described for some organic TADF emitters, albeit with slower rate constants [40, 41, 42].

(6) $$K_{eq}\;\left(T_{1}⇌S_{1}\right)=\frac{1}{3}\;\exp\left(-\Delta E_{S_{1}-T_{1}}/k_{B}T\right)$$

In contrast, **B** (*λ*_em_ = 621 nm, ϕ = 0.32, *k*_TADF_ = 8.7·10^5^ s^−1^) has been inferred to exhibit $k_{r}^{S1}$ ≈ 2·10^7^ s^−1^ and *ΔE*(S_1_–T_1_) = 523 cm^−1^ in the solid state [22]. The DPM model also hints at very fast *k_ISC_* = 2.38·10^10^ s^−1^, but much slower *k_RISC_* of only 3.4·10^8^ s^−1^, resulting in a significantly reduced *K*_eq_(T_1_ ⇄ S_1_) = 0.014. We hypothesize that the beneficial effect of the iPC ligand in **4** in comparison to the cAArC in **B** results from its through‐space conjugation [33], which reduces the energy gap of the ^1/3^LLCT states involved in the TADF process. The smaller *ΔE*(S_1_–T_1_) slows down ISC, but significantly enhances *k_RISC_* and, consequently, the overall *k*_TADF_ by a more favorable S_1_/T_1_ equilibrium. Furthermore, non‐radiative decay from the S_1_ state with $k_{nr}^{S1}$ (max/min) = 1.0/0.6·10^7^ s^−1^ is also reduced in **4** due to the higher steric demand of the iPC ligand in comparison to cAArC (**B**: $k_{nr}^{S1}$ = 3.3·10^7^ s^−1^), adding to the enhanced photoluminescence quantum yield. The different effects of the smaller energy gap and the apparently important coupling to energetically higher lying excited states on the ISC/RISC processes require further sophisticated studies, which are outside the scope of this work. However, it is important to note that the RISC process in **4** is 2–4 orders of magnitude faster than observed in prominent organic NIR‐TADF emitters, which are dominantly prompt fluorescence emitters and for which triplet state deactivation is determined by *k_RISC_*, while in **4** it is by the $k_{r}^{S1}$ [6, 7, 25, 26, 27, 28, 29, 43].

**FIGURE 5:**
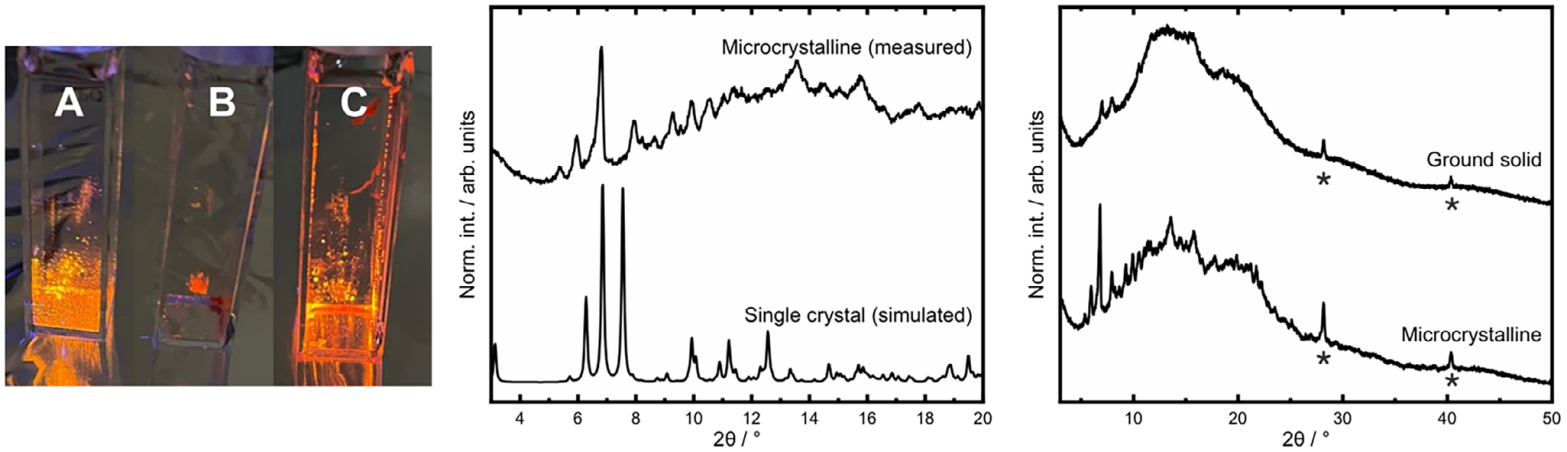
Left: Luminescence of **4** irradiated at 365 nm in the crystalline solid state (A), after grinding (B) and upon THF vapor exposure (C). Middle: PXRD pattern of the microcrystalline bulk sample of **4** in comparison to the pattern simulated from the crystal structure. Right: PXRD patterns of microcrystalline **4** before (A) and after grinding (B).

Upon grinding of **3–6**, the emission is severely bathochromically shifted into the deep red to near‐IR with *λ*_max_ = 665–700 nm (Figure 4), while high *k*_TADF_ of 7.2‐9.9·10^5^ s^−1^ are maintained that can still compete with nonradiative decay caused by the EGL, giving for such low emission energies involving triplet states high *Φ*_PL_ of up to 0.22 (Table 1). According to our TR‐VT measurements of **4**, the dominant luminescence mechanism is still TADF and the decrease in *k_r_* in comparison to the single‐crystalline solid state appears to originate from a slightly increased *ΔE*(S_1_–T_1_) = 364 cm^−1^ in combination with a longer *τ*_F_ of 90 ns (Figure 4). The process of mechanochromic TADF is reversible upon exposure to THF vapor, recovering the original emission properties (Figures 5 and S46).

We have recently shown for [Cu(Cz)(BINAP)] complexes that the luminescence energy and efficiency of the TADF process depends on intermolecular C─H⋯π interactions between the electron‐rich donor and electrophilic acceptor ligands of the emitter molecule with the neighboring complexes, which stabilizes the respective partial charges in the highly polar ground state S_0_ [35, 44]. Consequently, these interactions destabilize the less polar ^1/3^CT excited states characterized by an inverted and smaller permanent dipole moment, which the rigidly packed solid‐state environment cannot respond to by reorientation as typically observed in solution, i.e. solvatochromism. According to our SC‐XRD studies, **3–6** also exhibit extensive hydrogen bonding between the Cz donor and iPC acceptor units of neighboring complex molecules (Figures 2 and S19–S22), and their disruption by mechanical grinding should decrease the energy gap between the then destabilized ground state S_0_ and the ^1/3^LLCT states. Indeed, the ground powder samples exhibit bathochromic shifts of the lowest excitation band of 50–100 nm, which are most pronounced for **3** and **4** as found for the relative shifts of *λ*_em_ (Figures 4 and S27–S30, S34–S37, Table 1). Powder X‐ray diffraction (PXRD) studies indicate that the microcrystalline samples turn largely amorphous upon grinding, breaking up the symmetric packing as observed for [Cu(Cz)(BINAP)] (Figure 5) [44].

**FIGURE 6:**
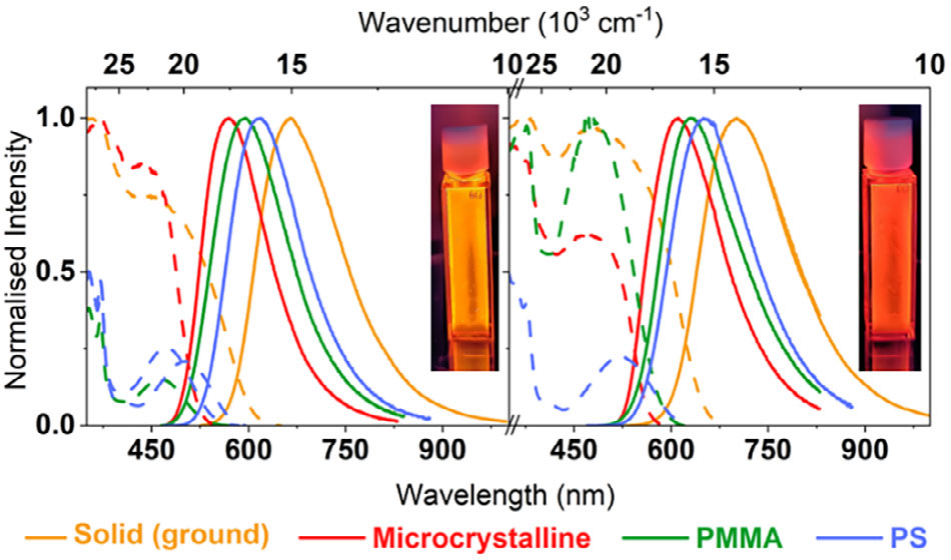
Excitation (dashed) and emission (solid) spectra of **3** (left) and **4** (right) in different environments at 297 K (*λ*_ex_ = 475 nm). Photographs under UV lamp irradiation of **3** and **4** in polystyrene films.

Important for potential technological applications of this class of emitters is the finding that environmental control over the luminescence properties is also possible when **3–6** are doped in organic polymer matrices. Although polar polymethylmetacrylate (PMMA), which undergoes hydrogen bonding with polar moieties (i.e. the respective ligands), leads to emission spectra very similar to the crystalline solid state, less polar polystyrene (PS) provides films with luminescence properties which resemble more those of the ground powders (Figures 6, S27–S30, S34–S37 and Table 1).

### 2.3 Electroluminescence

**FIGURE 7:**
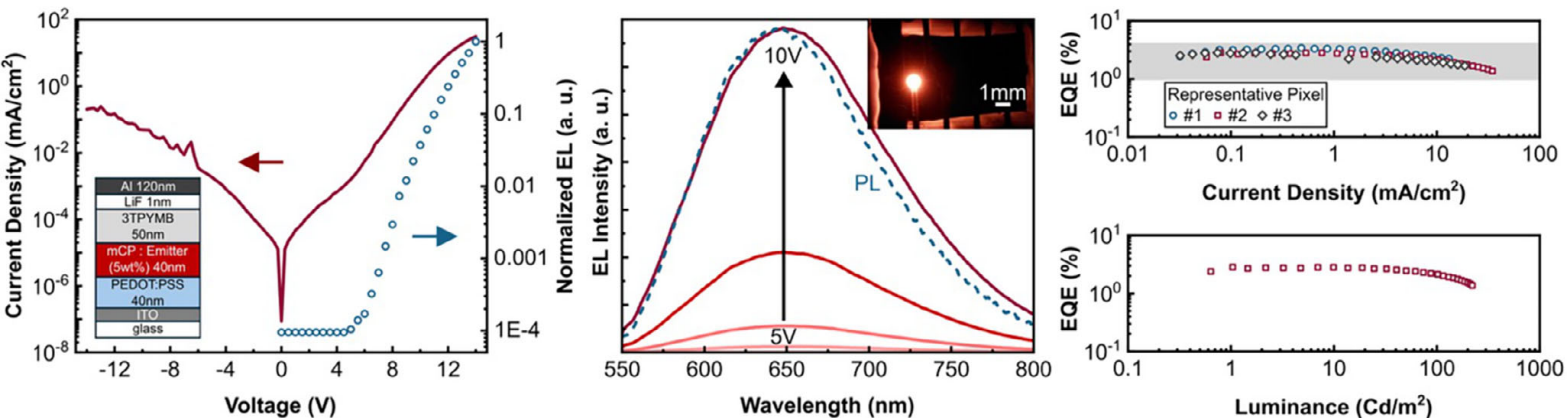
Basic characteristics of a prototypical OLED with **4** as deep red emitter. Left: Current density‐voltage‐electroluminescence characteristics (−14 to 14 V) in semilogarithmic depiction for a representative pixel. The device architecture is shown as inset. Middle: EL spectra measured under constant voltage operation from 5up to 10 V (inset: photograph of a representative pixel). The comparative PL spectrum in mCP (*λ_ex_* = 532 nm, dashed line) is scaled to the emission at 10 V. Right: *EQE* as function of the current density as well as the luminance. In case of the current density dependence, three different pixels (#1, #2, and #3) are shown with the representative *EQE* ranging from 1% to 4% as highlighted by the grey box. The luminance characteristics shown refer to pixel #2.

To demonstrate the general applicability of the Cu^I^ iPC complexes as electroluminescent emitter materials, we have implemented the Cz^tBu^ derivative **4** in a prototypical OLED architecture with a solution‐processed emissive layer. We employed the weakly polar host material 1,3‐bis(N‐carbazolyl)benzene (mCP) doped with 5‐wt % of **4** to conserve the deep red emission observed in non‐polar PS. The transient PL data of **4** dispersed in the mCP host are shown in Figure S42. The full device architecture displayed is displayed as inset in Figure 7 (left). The current density‐voltage‐electroluminescence (*J*‐*V*‐*EL*) characteristics demonstrate the stable device operation in a voltage regime from −14 to 14 V. The onset of light emission at around 5 V is accompanied by a change in slope of the *J*‐*V* characteristics and, additionally, the *EL* scales almost exponentially with increasing current density, respectively voltage. The absolute current density stays below 100 mA·cm^−2^ at 14 V with a pronounced *J*‐*V* dissymmetry of about two orders of magnitude when compared to the reverse bias direction, which is indicative of the absence of low ohmic parasitic pathways. The spectral emission of the OLED is voltage independent and resembles the PL spectrum of **4** in the same matrix material (blue dashed line) with an emission maximum centered at around 650 nm (see Figure 7, middle and Figure S43). Hence, the OLED emission can be clearly assigned to EL of the Cu^I^ iPC complex. The photograph given as inset in Figure 7 (middle) demonstrates the deep red appearance and the homogenous pixel emission. The external quantum efficiency (*EQE*) as function of *J* as well as of the luminance is depicted in Figure 7 (right). The *EQE* curves measured on three different pixels (top graph) highlight the reproducibility of our OLED configuration with an *EQE* within 1% to 4% over the full current density regime and with an identical roll‐off behaviour for all pixels. The maximum *EQE* is reached between 0.1 and 1 mA·cm^−2^ before the weakly pronounced roll‐off sets in. The *EQE* remains well above 1 % up to 100 mA·cm^−2^, which we attribute to efficient triplet up‐conversion and hence, the suppression of non‐radiative exciton annihilation processes. Our maximum *EQE* of 3.5 % is competitive to other deep‐red and NIR emitters, as values approaching 10 % are rarely reported and higher EQEs are typically achieved by addition of Ir^III^ or Pt^II^ complexes as FRET sensitizers driving the prompt fluorescence of the organic NIR‐TADF emitter [7, 26, 27, 43]. Luminance values above 200 Cd·m^−2^ are achieved at an *EQE* of 1 to 2 % (bottom graph). We note that the comparison of luminance values, which constitute a photopic measure, is not necessarily meaningful for emitter materials with significant radiation contributions outside the visible spectral range. The transient EL data (Figures S44 and S45) indicate the presence of excess charge carriers accumulated at interfaces or located at trap states, which results in a transient EL overshoot following a slow decay component [45, 46]. Thus, despite the reasonable *EQE* achieved for our prototypical OLED, the Cu^I^ iPC complex is not yet operating at its limit, as the EQE is restricted by the stack architecture used and not the emitter itself. Overall, the good performance of **4** under electroluminescent conditions highlights the potential of Cu^I^ iPC complexes as TADF materials with emission in the deep red to NIR.

## 3 Conclusion

We have reported the application of the paracyclophane‐based carbene iPC as excited state CT π‐acceptor in copper(I) carbazolate complexes. In comparison with structurally related cAAC and cAArC complexes, the through‐space coupling of the iPC ligand reduces the energy gap between the ^1/3^LLCT states and the ground state S_0_, which lowers the emission energy into the deep red to near‐IR. In addition, the iPC ligand greatly enhances the efficiency of the TADF process with very high *k*_TADF_ of the Cu^I^ complexes of up to 1.9·10^6^ s^−1^ in combination with, for such low energy emission (*λ* > 600 nm), very high photoluminescence quantum yields. With these properties, the Cu^I^ iPC complexes are highly efficient deep‐red emitters and amongst the fastest TADF materials to date in general, only surpassed by a few silver(I) and gold(I) complexes with regard to *k*_TADF_, and outcompete traditional emitters that can exploit triplet excited states based on heavy elements such as Ir^III^ and Pt^II^. The kinetic excited state analysis suggests that the iPC ligand greatly enhances the RISC process (*k_RISC_* = ca. 0.6‐21·10^9^ s^−1^) and that $k_{r}^{S1}$ is the rate‐determining factor for the TADF. This distinguishes the low energy Cu^I^ emitters from established organic deep red to NIR TADF emitters, which exhibit comparably low *k_RISC_* and high *k_F_* of the order of 10^4^–10^6^ and 10^7^–10^8^ s^−1^, respectively, and are thus dominantly prompt fluorescent dyes. Also, this new class of emitters is generally applicable in electroluminescent devices and thus demonstrates a possible approach of making the related light sources compatible with optical fiber networks. Our findings indicate that there is further potential to overcome the limitations of the EGL by designing the ligand´s electronic structure and thereby strengthening, in general, the research on molecular NIR emitters for photonic IT applications

## Author Contributions

**Sabyasachi Maity**: writing – original draft, validation, investigation, data curation, visualization. **Andrei Belyaev**: data curation, writing – review and editing. **Björn Ewald**: data curation, writing – review and editing, writing – original draft, investigation, formal analysis. **Andreas Steffen**: conceptualization, funding acquisition, writing – original draft, writing – review and editing, visualization, formal analysis, supervision, resources, project administration. **Jan–Benedikt Weiß**: data curation, writing – review and editing. **André M. T. Muthig**: writing – original draft, investigation, data curation, visualization. **Sebastian Henke**: supervision, writing – review and editing, funding acquisition. **Justin Krieger**: data curation, investigation, writing – review and editing. **Benjamin Hupp**: writing – review and editing, supervision, formal analysis. **Jens Pflaum**: supervision, writing – review and editing, formal analysis. **Andreas Prüfer**: data curation, investigation, writing – review and editing.

## Conflicts of Interest

The authors declare no conflicts of interest.

## Supporting information



The Supporting Information contain details of synthesis, NMR and structural characterization [47], luminescence and DFT data. The authors have cited additional references within the Supporting Information [48, 49, 50, 51, 52, 53, 54, 55, 56, 57, 58, 59].**Supporting File 1**: anie72342‐sup‐0001‐SuppMat.Pdf.

**Supporting File 2**: anie72342‐sup‐0002‐DataFile.Zip.

## Data Availability

The data that supports the findings of this study are available in the Supporting Information of this article.
