# Supplementary material for: Highly Efficient Mechanochromic Thermally Activated Delayed Fluorescence in the Deep Red to Near‐Infrared in Copper(I) [2.2]Isoindolinophanyl‐Carbene Carbazolates
Source: Angew Chem Int Ed Engl. 2026 Apr 25;65(24):e5338298. doi: 10.1002/anie.5338298 (PMC13245606; doi:10.1002/anie.5338298)
Supplement: Supplementary file 1 — The Supporting Information contain details of synthesis, NMR and structural characterization [47], luminescence and DFT data. The authors have cited additional references within the Supporting Information [48, 49, 50, 51, 52, 53, 54, 55, 56, 57, 58, 59].Supporting File 1: anie72342‐sup‐0001‐SuppMat.Pdf. [file ANIE-65-e5338298-s002.pdf]

1. GENERAL PROCEDURES 1
2. CHARACTERIZATION DATA 2
3. NMR SPECTRA 8
4. X-RAY CHARACTERIZATION DATA 14
5. STRUCTURAL DETAILS 20
6. PHOTOPHYSICAL MEASUREMENTS 25
7. UV-VISIBLE SPECTROSCOPY 26
8. EMISSION STUDIES 27
9. TD-DFT CALCULATIONS 36
10. OLED FABRICATION 41
11. MECHANOCHROMIC TADF 45
12. REFERENCES 47

1. General Procedures

All manipulations were carried out under an inert atmosphere of argon using standard Schlenk link[1] or glovebox techniques (GS MEGA E-Line, <0.5 ppm of H_2O and O_2). All reagents were used as supplied. Solvents such as tetrahydrofuran (THF), dichloromethane (DCM), cyclohexane, diethyl ether and n-pentane were used at HPLC grade purity from commercial sources (VWR and Fisher Chemicals) and dried using PureSolv MD 7 drying system.

NMR spectra were recorded on a Bruker Avance III HD NanoBay 400 or 600 MHz; H and chemical shifts (δ) are given in ppm relative to TMS, coupling constants (J) in Hz. The solvent signals were used as references, and the chemical shifts were converted to the TMS scale. CHN elemental analyses were performed on a Micro cube (Elementar).

(HiPC)(OTf), 1-Methyl-9H-carbazole and 1-Methoxy-9H-carbazole were synthesised according to literature procedures.[2,3]

The potassium salts of 9H-carbazole, 3,6-ditert-butyl-9H-carbazole, 1-Methyl-9H-carbazole, and 1-Methoxy-9H-carbazole were prepared by deprotonating the respective carbazoles with KHMDS in a 1:1 ratio in diethyl ether. After 24 hours, the respective potassium salt precipitated from the solution and was washed twice with 4 ml of diethyl ether. The spectroscopic data matches the reported literature.

2. Characterization Data

Synthesis of **1**

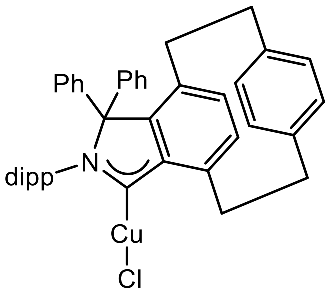

At -85°C, a solution of 69 mg (0.43 mmol, 1.00 equiv.) KHMDS in 2 ml THF was slowly added to a suspension of 300 mg (423 μmol, 1.00 equiv.) (HiPC)(OTf) and 70 mg copper(I) chloride dimethyl sulfide complex (0.44 mmol, 1.05 equiv.) in 20 ml THF. The yellow-orange suspension was warmed to room temperature and stirred overnight. All volatile components of the yellow-green suspension were removed under reduced pressure. After extraction with DCM and filtration over basic aluminum oxide, the filtrate was reduced to one-third volume, and the crude product was precipitated by the addition of n-pentane and washed twice with 2 ml n-pentane. The product was obtained by vapour diffusion of n-pentane into a THF/cyclohexane solution as orange crystals of compound **1** crystallized as 2:3 with THF (112 mg, 170 μmol, 40%).

The product slowly decomposes, and yellow-green crystals of (HiPC)(OTf) can be isolated from the decomposed mixture. To minimize the decomposition process, compound **1** was kept at -35°C inside the glovebox.

^{1}H-NMR (THF-d_8, 600 MHz, 298 K): 7.86 – 7.76 (m, 1H, Ar–H), 7.70 – 7.61 (m, 1H, Ar–H), 7.43 (tt, J = 7.3, 1.2 Hz, 1H, Ar–H), 7.37 – 7.30 (m, 1H, Ar–H), 7.27 – 7.21 (m, 4H, Ar–H), 7.17 – 6.97 (br, 2H, Ar–H), 6.94 (d, J = 8.1 Hz, 1H, Ar–H), 6.90 (dd, J = 7.7, 2.0 Hz, 1H, Ar–H), 6.78 (dd, J = 7.7, 1.9 Hz, 1H, Ar–H), 6.73 (dd, J = 5.4, 3.8 Hz, 1H, Ar–H), 6.66 (d, J = 7.6 Hz, 1H, Ar–H), 6.59 – 6.55 (m, 1H, Ar–H), 6.37 (dd, J = 8.2, 2.0 Hz, 1H, Ar–H), 5.00 (dd, J = 8.2, 2.0 Hz, 1H, Ar–H), 4.80 (ddd, J = 13.7, 10.4, 4.9 Hz, 1H, Ar–H), 3.54 (q, J = 6.7 Hz, 1H), 3.51 – 3.44 (m, 1H), 3.38 (q, J = 7.0 Hz, 1H), 3.22 (ddd, J = 13.3, 10.8, 4.9 Hz, 1H), 2.97 – 2.89 (m, 1H), 2.70 (ddd, J = 13.3, 10.2, 6.0 Hz, 1H), 2.53 (ddd, J = 14.1, 10.0, 6.0 Hz, 1H), 2.40 (ddd, J = 12.7, 9.9, 2.0 Hz, 1H), 1.35 (d, J = 6.7 Hz, 3H), 1.10 (dt, J = 13.0, 6.8 Hz, 2H), 0.48 (d, J = 6.7 Hz, 3H), 0.80 (d, *J* = 6.7 Hz, 3H), -0.20 (d, J = 6.7 Hz, 3H); **^{13}C {^{1}H} NMR** (THF-d_8, 151 MHz, 298 K): 229.60 (*C*Cu), 149.78 (Ar–CH), 146.03 (Ar–CH), 144.98 (Ar–CH), 143.28 (Ar–CH), 143.25 (Ar–CH), 140.54 (Ar–CH), 139.79 (Ar–CH), 139.49 (Ar–CH), 139.10 (Ar–CH), 136.55 (Ar–CH), 136.24 (Ar–CH), 134.89 (Ar–CH), 133.70 (Ar–CH), 133.05 (Ar–CH), 133.00 (Ar–CH), 132.77 (Ar–CH), 131.73 (Ar–CH), 131.13 (Ar–CH), 130.48 (Ar–CH), 129.65 (Ar–CH), 129.37 (Ar–CH), 128.43 (Ar–CH), 126.04 (Ar–CH), 124.52 (Ar–CH), 96.02, 35.78 (CH_2), 35.02 (CH_2), 34.57 (CH_2), 30.76 (CH_2), 30.49 (CH), 30.05 (CH), 27.25 (CH_3), 26.37 (CH_3), 23.35 (CH_3), 21.53 (CH_3); **EA** calc. for [$(C_{42}H_{41}ClCuN)_2.((CH_2)_4O)_3$] C, 75.17; H, 6.97; N, 1.83; found C: 75.1; H: 6.9; N: 1.9.

Synthesis of **2**

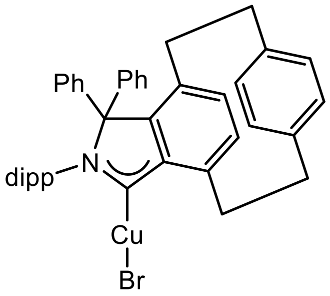

At -85°C, a solution of 69 mg (0.43 mmol, 1.00 equiv.) KHMDS in 2 ml THF was slowly added to a suspension of 300 mg (423 μmol, 1.00 equiv.) (HiPC)(OTf) and 87 mg copper(I) bromide dimethyl sulfide complex (0.43 mmol, 1.05 equiv.) in 20 ml THF. The yellow-orange suspension was warmed to room temperature and stirred overnight. All volatile components of the yellow-green suspension were removed under reduced pressure. After extraction with DCM and filtration over basic aluminum oxide, the filtrate was reduced to one-third volume, and the crude product was precipitated by the addition of n-pentane and washed twice with 2 ml n-pentane. The product was obtained by vapour diffusion of n-pentane into a THF/cyclohexane solution as orange crystals of compound **2** (21 mg, 30 μmol, 7%).

Low yield was observed for **2** in comparison to **1**, maybe due to incorporation of air or moisture during the reaction process.

The product slowly decomposes, and yellow-green crystals of (HiPC)(OTf) can be isolated from the decomposed mixture. To minimize the decomposition process, compound **2** was kept at -35°C inside the glovebox.

^{1}H-NMR (THF-d_8, 400 MHz, 298 K): 7.88 – 7.78 (br, 1H, Ar–H), 7.70 – 7.61 (br, 1H, Ar–H), 7.43 (tt, *J* = 7.3, 1.1 Hz, 1H, Ar–H), 7.36 – 7.29 (br, 1H, Ar–H), 7.28 – 7.19 (m, 4H, Ar–H), 7.15 – 7.03 (br, 2H, Ar–H), 6.97 – 6.91 (br, 1H, Ar–H), 6.90 (dd, *J* = 7.7, 2.0 Hz, 1H, Ar–H), 6.78 (dd, *J* = 7.7, 2.0 Hz, 1H, Ar–H), 6.73 (dd, *J* = 5.5, 3.8 Hz, 1H, Ar–H), 6.66 (dd, *J* = 7.6, 0.9 Hz, 1H, Ar–H), 6.57 (dd, *J* = 7.6, 0.9 Hz, 1H, Ar–H), 6.38 (dd, *J* = 8.2, 2.0 Hz, 1H, Ar–H), 5.01 (dd, *J* = 8.2, 2.0 Hz, 1H, Ar–H), 4.81 (ddd, *J* = 13.5, 10.3, 4.8 Hz, 1H Ar–H), 3.55 – 3.50 (m, 1H), 3.49 – 3.44 (m, 1H), 3.38 (q, *J* = 7.0 Hz, 1H), 3.22 (ddd, *J* = 13.2, 10.7, 4.9 Hz, 1H), 2.93 (dd, *J* = 13.9, 10.4 Hz, 1H), 2.70 (ddd, *J* = 13.1, 10.2, 5.9 Hz, 1H), 2.53 (ddd, *J* = 13.7, 9.8, 5.8 Hz, 1H), 2.48 – 2.35 (m, 1H), 1.35 (d, *J* = 6.7 Hz, 3H), 1.17 – 1.03 (m, 2H), 0.81 (d, *J* = 6.7 Hz, 3H), 0.47 (d, *J* = 6.7 Hz, 3H), -0.21 (d, *J* = 6.7 Hz, 3H); **EA** calc. for [$C_{42}H_{41}BrCuN$] C, 71.73; H, 5.88; N, 1.99; found C: 71.4; H: 6.0; N: 1.8.

Synthesis of **3**

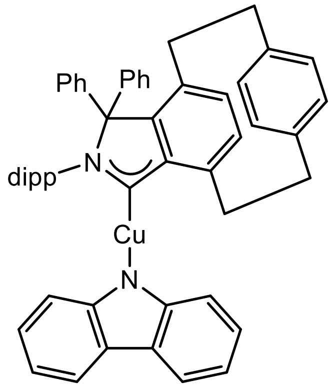

In a 20 ml scintillation vial, 16 mg (78 μmol, 1.03 equiv.) KCz (potassium salt of 9H-carbazole) was added to a solution of 50 mg (76 μmol, 1.0 equiv.) [CuCl(iPC)] **1** in 4 ml THF and stirred overnight. The deep red suspension was mixed with 2 ml diethyl ether and filtered over basic aluminum oxide and concentrated to one-fourth of the volume under reduced pressure. The product was obtained by vapour diffusion of a mixture of cyclohexane and n-pentane into the solution as yellow crystals of compound **3** crystallized as 2:3 with THF (45 mg, 57 μmol, 75%).

^{1}H-NMR (THF-d$_8$, 600 MHz, 298 K): 7.96 (d, *J* = 8.2 Hz, 1H, Ar–H), 7.83 (dt, *J* = 7.5, 1.4 Hz, 2H, Ar–H), 7.67 (t, *J* = 7.8 Hz, 1H, Ar–H), 7.49 (t, *J* = 7.8 Hz, 1H, Ar–H), 7.46 – 7.42 (m, 1H, Ar–H), 7.41 – 7.34 (m, 2H, Ar–H), 7.29 (t, *J* = 7.1 Hz, 1H, Ar–H), 7.17 (br, 2H, Ar–H), 7.00 (d, *J* = 8.2 Hz, 1H, Ar–H), 6.97 (d, *J* = 1.4 Hz, 1H, Ar–H), 6.96 (q, *J* = 1.6 Hz, 2H, Ar–H), 6.96 – 6.93 (m, 2H, Ar–H), 6.81 (q, *J* = 2.2 Hz, 1H, Ar–H), 6.79 (d, *J* = 1.0 Hz, 1H, Ar–H), 6.78 (d, *J* = 0.8 Hz, 1H, Ar–H), 6.77 (d, *J* = 1.1 Hz, 1H, Ar–H), 6.74 (q, *J* = 1.0 Hz, 1H, Ar–H), 6.73 – 6.70 (m, 2H, Ar–H), 6.64 – 6.58 (m, 1H, Ar–H), 6.50 (dd, *J* = 8.1, 2.2 Hz, 1H, Ar–H), 5.15 – 5.12 (m, 1H, Ar–H), 5.11 – 5.08 (m, 1H), 3.89 (dd, *J* = 13.7, 10.6 Hz, 1H), 3.74 – 3.67 (m, 1H), 3.66 – 3.63 (m, 1H), 3.37 (dtd, *J* = 9.2, 7.2, 5.3 Hz, 1H), 2.97 (dd, *J* = 14.0, 10.6 Hz, 1H), 2.73 (tdd, *J* = 10.0, 7.1, 4.2 Hz, 1H), 2.60 (ddd, *J* = 14.8, 10.0, 5.9 Hz, 1H), 2.42 (t, *J* = 11.6 Hz, 1H), 1.28 – 1.21 (m, 1H), 1.13 (d, *J* = 6.9 Hz, 3H), 0.87 (d, *J* = 8.2 Hz, 3H), 0.50 (d, *J* = 6.7 Hz, 3H), -0.16 (d, *J* = 6.7 Hz, 3H); **^{13}C {^{1}H} NMR** (THF-d$_8$, 151 MHz, 298 K): 231.03 (*C*Cu), 151.14 (Ar–CH), 150.52 (Ar–CH), 146.54 (Ar–CH), 144.87 (Ar–CH), 143.52 (Ar–CH), 140.41 (Ar–CH), 140.04 (Ar–CH), 139.62 (Ar–CH), 136.53 (Ar–CH), 136.46 (Ar–CH), 135.10 (Ar–CH), 133.80 (Ar–CH), 133.23 (Ar–CH), 133.09 (Ar–CH), 132.80 (Ar–CH), 131.25 (Ar–CH), 130.68 (Ar–CH), 129.77 (Ar–CH), 129.49 (Ar–CH), 126.64 (Ar–CH), 125.55 (Ar–CH), 125.27 (Ar–CH), 123.66 (Ar–CH), 119.56 (Ar–CH), 115.78 (Ar–CH), 115.22 (Ar–CH), 96.17, 35.84 (CH_2), 35.23 (CH_2), 35.06 (CH_2), 30.99 (CH_2), 30.57 (CH), 30.24 (CH), 27.01 (CH_3), 26.37(CH_3), 23.41 (CH_3), 21.89 (CH_3); **EA** calc. for [$(C_{54}H_{49}CuN_2)_2.((CH_2)_4O)_3$] C, 80.28; H, 6.85; N, 3.12; found C: 79.9; H: 6.9; N: 3.3.

Synthesis of **4**

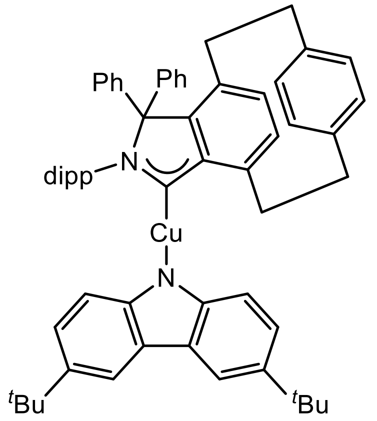

In a 20 ml scintillation vial, 28 mg (77 μmol, 1.00 equiv.) [KCz^{tBu} · $(Et_2O)_{2/3}$] (potassium salt of 3,6-ditert-butyl-9H-carbazole) was added to a solution of 50 mg (76 μmol, 1.00 equiv.) [CuCl(iPC)] **1** in 4 ml THF and stirred overnight. The deep red suspension was mixed with 2 ml diethyl ether and filtered over basic aluminum oxide and concentrated to one-fourth of the volume under reduced pressure. The product was obtained by vapour diffusion of a mixture of cyclohexane and n-pentane into the solution as deep yellow crystals of compound **4** crystallised as 4:5 with THF (51 mg, 57 μmol, 75%).

^{1}H-NMR (d_8-THF, 600 MHz, 298 K): 7.94 (d, *J* = 8.2 Hz, 1H, Ar–H), 7.90 (d, *J* = 2.0 Hz, 2H, Ar–H), 7.66 (t, *J* = 7.8 Hz, 1H, Ar–H), 7.49 (t, *J* = 7.8 Hz, 1H, Ar–H), 7.43 (tt, *J* = 7.4, 1.1 Hz, 1H, Ar–H), 7.39 (d, *J* = 1.5 Hz, 1H, Ar–H), 7.37 (d, *J* = 1.5 Hz, 1H, Ar–H), 7.35 – 7.30 (br, 1H, Ar–H), 7.30 – 7.25 (m, 1H, Ar–H), 7.23 – 7.10 (br, 2H, Ar–H), 7.09 (d, *J* = 2.1 Hz, 1H, Ar–H), 7.07 (d, *J* = 2.1 Hz, 1H, Ar–H), 7.05 – 6.98 (m, 1H, Ar–H), 6.98 – 6.96 (m, 1H, Ar–H), 6.96 (d, *J* = 1.5 Hz, 1H, Ar–H), 6.90 (dd, *J* = 7.6, 1.9 Hz, 1H, Ar–H), 6.76 (dd, *J* = 7.7, 1.9 Hz, 1H, Ar–H), 6.68 (d, *J* = 8.4 Hz, 2H, Ar–H), 6.63 – 6.58 (m, 1H, Ar–H), 6.55 – 6.51 (m, 1H, Ar–H), 6.47 (dd, *J* = 8.2, 2.0 Hz, 1H, Ar–H), 5.13 – 5.10 (m, 1H, Ar–H), 5.09 (d, *J* = 5.8 Hz, 1H), 3.82 (ddd, *J* = 13.6, 10.6, 2.9 Hz, 1H), 3.67 (ddd, *J* = 13.9, 10.7, 3.2 Hz, 1H), 3.28 (ddd, *J* = 13.2, 10.7, 5.1 Hz, 1H), 2.94 (ddd, *J* = 13.7, 10.3, 2.2 Hz, 1H), 2.69 (ddd, *J* = 13.1, 10.2, 5.9 Hz, 1H), 2.58 (ddd, *J* = 13.9, 9.9, 5.9 Hz, 1H), 2.41 (ddd, *J* = 12.7, 10.0, 1.8 Hz, 1H), 1.40 (s, 18H), 1.27 – 1.18 (m, 2H), 1.12 (d, *J* = 7.0 Hz, 3H), 0.84 (d, *J* = 6.7 Hz, 3H), 0.49 (d, *J* = 6.7 Hz, 3H), -0.17 (d, *J* = 6.6 Hz, 3H); **^{13}C {^{1}H} NMR** (d_8-THF, 151 MHz, 298 K): 231.18 (*C*Cu), 150.47 (Ar–CH), 149.81 (Ar–CH), 146.50 (Ar–CH), 144.85 (Ar–CH), 143.50 (Ar–CH), 143.35 (Ar–CH), 140.42 (Ar–CH), 140.02 (Ar–CH), 139.96 (Ar–CH), 139.55 (Ar–CH), 137.73 (Ar–CH), 136.58 (Ar–CH), 136.37 (Ar–CH), 135.15 (Ar–CH), 133.86 (Ar–CH), 133.20 (Ar–CH), 133.06 (Ar–CH), 132.82 (Ar–CH), 131.19 (Ar–CH), 130.58 (Ar–CH), 129.72 (Ar–CH), 129.43 (Ar–CH), 126.60 (Ar–CH), 125.45 (Ar–CH), 125.20 (Ar–CH), 121.26 (Ar–CH), 115.47 (Ar–CH), 114.70 (Ar–CH), 96.06, 35.82 (CH_2), 35.19 (CH_2), 35.06 (CH_2), 35.02 (CH_2), 32.79, 30.96 (CH), 30.23 (CH), 27.06 (CH_3), 26.37 (CH_3), 23.40 (CH_3), 23.20 ($CH_3(Cz^{tBu})$), 21.88 (CH_3); **EA** calc. for $[(C_{62}H_{65}CuN_2)_4.((CH_2)_4O)_5]$ C, 81.13; H, 7.62; N, 2.82; found C: 81.0; H: 7.3; N: 3.1.

Synthesis of **5**

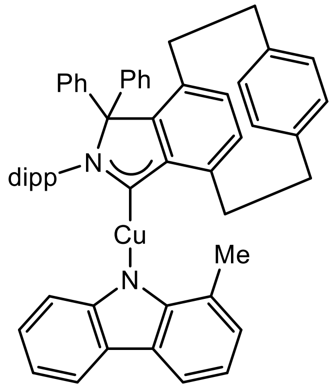

In a 20 ml scintillation vial, 17 mg (78 μmol, 1.00 equiv.) $K^{Me}Cz$ (potassium salt of 1-Methyl-9H-carbazole) was added to a solution of 50 mg (76 μmol, 1.00 equiv.) [CuCl(iPC)] **1** in 4 ml THF and stirred overnight. The deep red suspension was mixed with 2 ml diethyl ether and filtered over basic aluminum oxide and concentrated to one-fourth of the volume under reduced pressure. The product was obtained by vapour diffusion of a mixture of cyclohexane and n-pentane into the solution as deep yellow crystals of compound **5** crystallised as 2:3 with THF (48 mg, 60 μmol, 79%).

The crystals obtained from the above procedure have poor reflections and thus poor data quality in SC-XRD measurements. The crystals were then dissolved in DCM, and vapour diffusion of the mixture of n-pentane and cyclohexane gave yellow crystals of **5** with much better data quality for SC-XRD measurements.

^{1}H-NMR (THF-d_8,, 600 MHz, 298 K): 7.95 (d, *J* = 8.1 Hz, 1H, Ar–H), 7.87 (dd, *J* = 7.7, 0.7 Hz, 1H, Ar–H), 7.78 (dd, *J* = 7.8, 0.6 Hz, 1H, Ar–H), 7.74 (t, *J* = 7.6 Hz, 1H, Ar–H), 7.66 – 7.51 (br, 1H, Ar–H), 7.48 (tt, *J* = 7.3, 1.2 Hz, 1H, Ar–H), 7.39 (t, *J* = 7.8 Hz, 2H, Ar–H), 7.29 (td, *J* = 7.5, 3.8 Hz, 3H, Ar–H), 7.25 – 7.19 (m, 2H, Ar–H), 7.02 (ddd, *J* = 8.1, 5.5, 1.3 Hz, 3H, Ar–H), 6.93 (dt, *J* = 7.0, 1.1 Hz, 1H, Ar–H), 6.90 (dd, *J* = 7.6, 2.0 Hz, 1H, Ar–H), 6.86 (dd, *J* = 7.7, 1.6 Hz, 1H, Ar–H), 6.84 – 6.81 (m, 1H, Ar–H), 6.81 – 6.76 (m, 2H, Ar–H), 6.69 (dd, *J* = 7.5, 0.9 Hz, 1H, Ar–H), 6.61 (dd, *J* = 7.6, 0.9 Hz, 1H, Ar–H), 6.40 (dd, *J* = 8.1, 2.0 Hz, 1H, Ar–H), 5.06 (dd, *J* = 8.1, 2.0 Hz, 1H, Ar–H), 4.97 (ddd, *J* = 13.2, 10.4, 4.8 Hz, 1H), 3.53 (ddd, *J* = 13.4, 10.7, 3.5 Hz, 1H), 3.18 (ddd, *J* = 13.2, 10.7, 4.8 Hz, 1H), 3.03 – 2.95 (m, 1H), 2.74 (ddd, *J* = 13.0, 10.2, 5.8 Hz, 1H), 2.68 – 2.62 (m, 1H), 2.61 (s, 3H), 2.42 (ddd, *J* = 12.8, 9.9, 2.3 Hz, 1H), 1.29 – 1.22 (m, 2H), 1.12 (d, *J* = 7.0 Hz, 1H), 1.09 (d, *J* = 6.7 Hz, 3H), 0.94 (d, *J* = 6.7 Hz, 3H), 0.47 (d, *J* = 6.7 Hz, 3H), -0.13 (d, *J* = 6.7 Hz, 3H); **^{13}C {^{1}H} NMR** (THF-d_8, 151 MHz, 298 K): 231.57 (*C*Cu), 151.34 (Ar–CH), 149.96 (Ar–CH), 149.79 (Ar–CH), 146.10 (Ar–CH), 145.00 (Ar–CH), 143.72 (Ar–CH), 140.46 (Ar–CH), 140.13 (Ar–CH), 139.89 (Ar–CH), 139.68 (Ar–CH), 136.70 (Ar–CH), 136.56 (Ar–CH), 135.25 (Ar–CH), 133.92 (Ar–CH), 133.19 (Ar–CH), 132.90 (Ar–CH), 132.87 (Ar–CH), 131.93 (Ar–CH), 131.13 (Ar–CH), 130.77 (Ar–CH), 129.74 (Ar–CH), 129.55 (Ar–CH), 129.52 (Ar–CH), 128.35 (Ar–CH), 126.49 (Ar–CH), 126.03 (Ar–CH), 125.46 (Ar–CH), 125.02 (Ar–CH), 124.80 (Ar–CH), 123.42 (Ar–CH), 121.67 (Ar–CH), 119.69 (Ar–CH), 117.76 (Ar–CH), 116.01 (Ar–CH), 115.93 (Ar–CH), 115.83 (Ar–CH), 96.26, 35.92 (CH_2), 35.06 (CH_2), 34.78 (CH_2), 31.01 (CH_2), 30.70 (CH), 30.38 (CH), 27.13 (CH_3), 26.37(CH_3), 23.67 (CH_3), 21.89 (CH_3), 21.06 (CH_3(^{Me}Cz)); **EA** calc. for $[(C_{55}H_{51}CuN_2)_2.((CH_2)_4O)_3]$ C, 80.36; H, 6.97; N, 3.07; found C: 80.4; H: 6.9; N: 3.3.

Synthesis of **6**

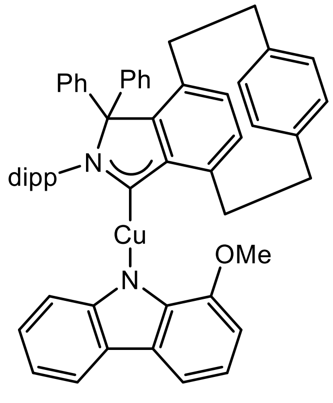

In a 20 ml scintillation vial, 20 mg (76 μmol, 1.00 equiv.) [$K^{OMe}Cz \cdot (Et_2O)_{1/3}$] (potassium salt of 1-Methoxy-9H-carbazole) was added to a solution of 50 mg (76 μmol, 1.00 equiv.) [CuCl(iPC)] **1** in 4 ml THF and stirred overnight. The deep red suspension was mixed with 2 ml diethyl ether and filtered over basic aluminum oxide and concentrated to one-fourth of the volume under reduced pressure. The product was obtained by vapour diffusion of a mixture of cyclohexane and n-pentane into the solution as deep yellow crystals of compound **6** crystallises as 1:1 with THF (46 mg, 56 μmol, 74%).

^{1}H-NMR (THF-d_8,, 600 MHz, 298 K): 8.01 (d, *J* = 8.1 Hz, 1H, Ar–H), 7.80 (ddd, *J* = 7.7, 1.4, 0.7 Hz, 1H, Ar–H), 7.73 (t, *J* = 7.7 Hz, 1H, Ar–H), 7.55 (dd, *J* = 7.6, 1.0 Hz, 1H, Ar–H), 7.47 (tt, *J* = 7.3, 1.1 Hz, 1H, Ar–H), 7.38 (t, *J* = 7.5 Hz, 1H, Ar–H), 7.27 (ddd, *J* = 7.3, 6.2, 1.8 Hz, 2H, Ar–H), 7.25 – 7.21 (m, 2H, Ar–H), 7.20 – 7.07 (m, 1H, Ar–H), 7.06 – 6.89 (m, 2H, Ar–H), 6.87 (ddd, *J* = 7.7, 3.3, 1.6 Hz, 2H, Ar–H), 6.83 (dt, *J* = 8.2, 0.9 Hz, 1H, Ar–H), 6.81 (d, *J* = 1.2 Hz, 1H, Ar–H), 6.80 – 6.79 (m, 2H, Ar–H), 6.78 (d, *J* = 1.8 Hz, 1H, Ar–H), 6.74 (ddt, *J* = 7.7, 5.9, 1.0 Hz, 2H, Ar–H), 6.67 (dt, *J* = 7.5, 1.2 Hz, 1H, Ar–H), 6.60 – 6.56 (m, 1H, Ar–H), 6.39 (dd, *J* = 8.1, 1.9 Hz, 1H, Ar–H), 5.02 (ddd, *J* = 13.6, 10.4, 5.0 Hz, 1H, Ar–H), 4.96 – 4.91 (m, 1H), 4.05 (s, 3H), 3.70 (p, *J* = 6.8 Hz, 1H), 3.56 – 3.51 (m, 1H), 3.44 (ddd, *J* = 13.4, 10.4, 3.1 Hz, 1H), 3.13 (ddd, *J* = 12.8, 10.9, 4.9 Hz, 1H), 3.01 – 2.94 (m, 1H), 2.72 (ddd, *J* = 12.9, 10.2, 6.0 Hz, 1H), 2.62 (ddd, *J* = 13.9, 9.9, 6.1 Hz, 1H), 2.48 – 2.41 (m, 1H), 1.33 – 1.29 (m, 1H), 1.27 (d, *J* = 6.7 Hz, 3H), 0.98 (d, *J* = 6.7 Hz, 3H), 0.52 (d, *J* = 6.7 Hz, 3H), -0.14 (d, *J* = 6.7 Hz, 3H); **^{13}C {^{1}H} NMR** (THF-d_8, 151 MHz, 298 K): 232.14 (*C*Cu), 150.63 (Ar–CH), 149.95 (Ar–CH), 148.35 (Ar–CH), 146.17 (Ar–CH), 145.61 (Ar–CH), 143.94 (Ar–CH), 143.39 (Ar–CH), 141.26 (Ar–CH), 140.60 (Ar–CH), 139.80 (Ar–CH), 139.72 (Ar–CH), 139.60 (Ar–CH), 136.53 (Ar–CH), 136.04 (Ar–CH), 135.38 (Ar–CH), 133.92 (Ar–CH), 133.08 (Ar–CH), 132.91 (Ar–CH), 132.80 (Ar–CH), 131.84 (Ar–CH), 131.11 (Ar–CH), 130.48 (Ar–CH), 129.65 (Ar–CH), 129.40 (Ar–CH), 128.25 (Ar–CH), 126.82 (Ar–CH), 126.20 (Ar–CH), 125.63 (Ar–CH), 124.79 (Ar–CH), 123.25 (Ar–CH), 119.56 (Ar–CH), 116.35 (Ar–CH), 115.69 (Ar–CH), 115.59 (Ar–CH), 113.32 (Ar–CH), 103.88 (Ar–CH), 96.16, 55.70 ($CH_3O(^{OMe}Cz)$), 36.02 (CH_2), 35.03 (CH_2), 34.93 (CH_2), 30.95 (CH_2), 30.70 (CH), 30.29 (CH), 27.08 (CH_3), 25.78 (CH_3), 23.54 (CH_3), 21.88 (CH_3); **EA** calc. for [$(C_{55}H_{51}CuN_2)_2.((CH_2)_4O)_3$] C, 79.47; H, 6.67; N, 3.14; found C: 79.4; H: 6.3; N: 3.4.

3. NMR spectra

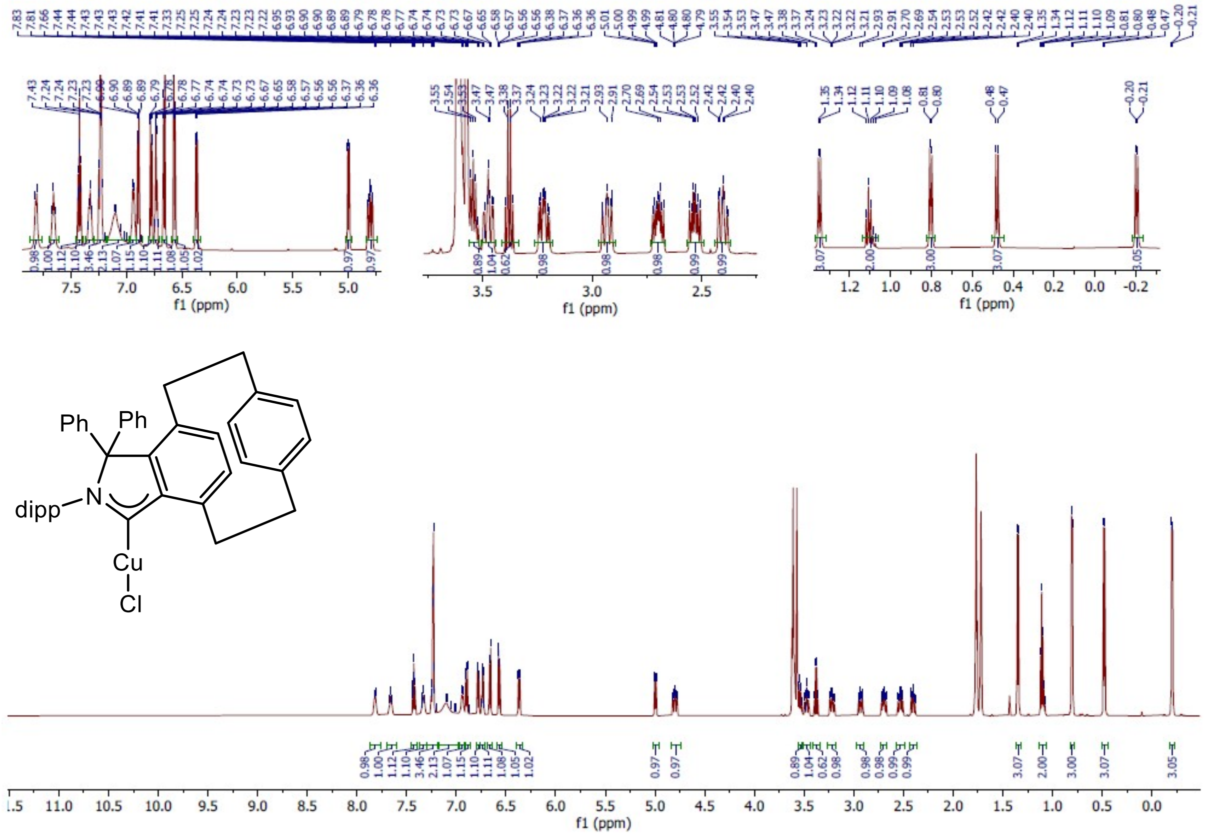

Figure S1.^{1}H-NMR (600 MHz, THF-d_8, 298 K) of **1**.

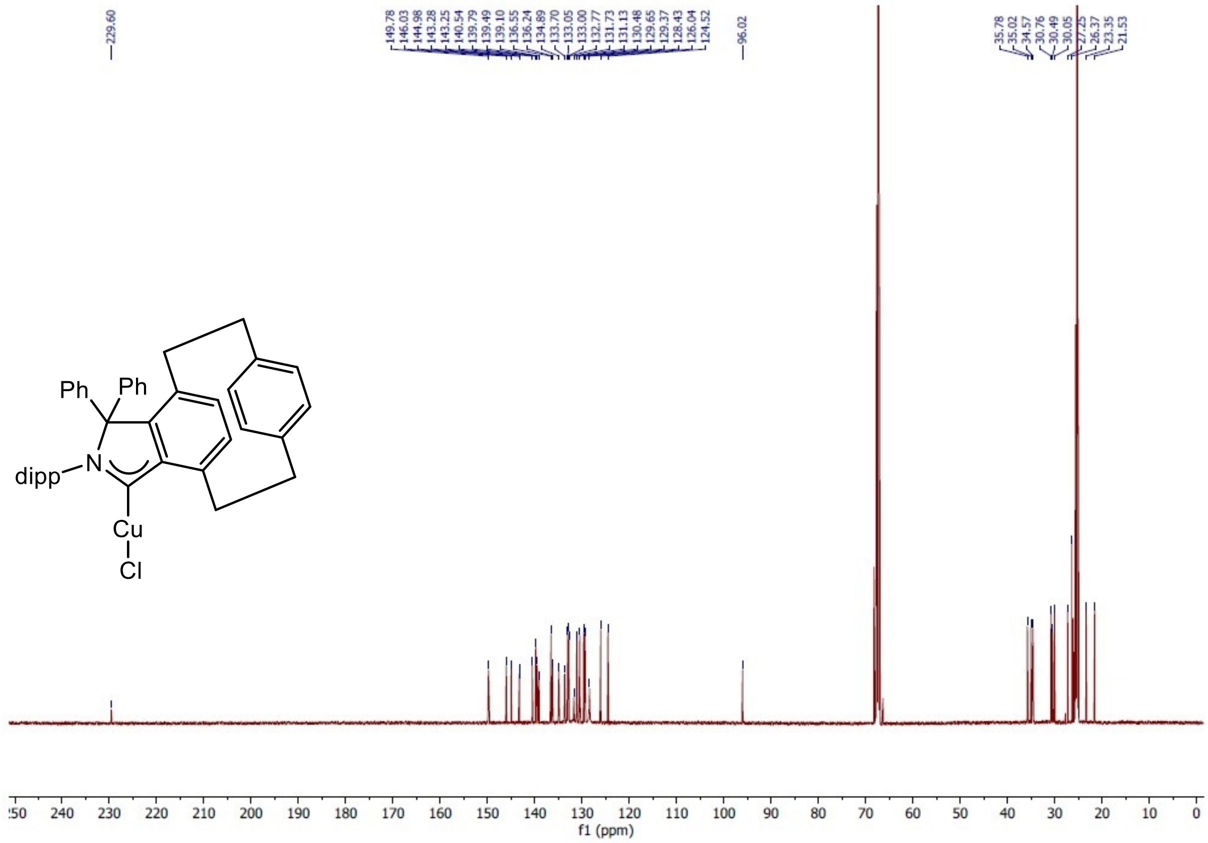

Figure S2. ^{13}C-NMR (151 MHz, THF-d_8, 298 K) of **1**.

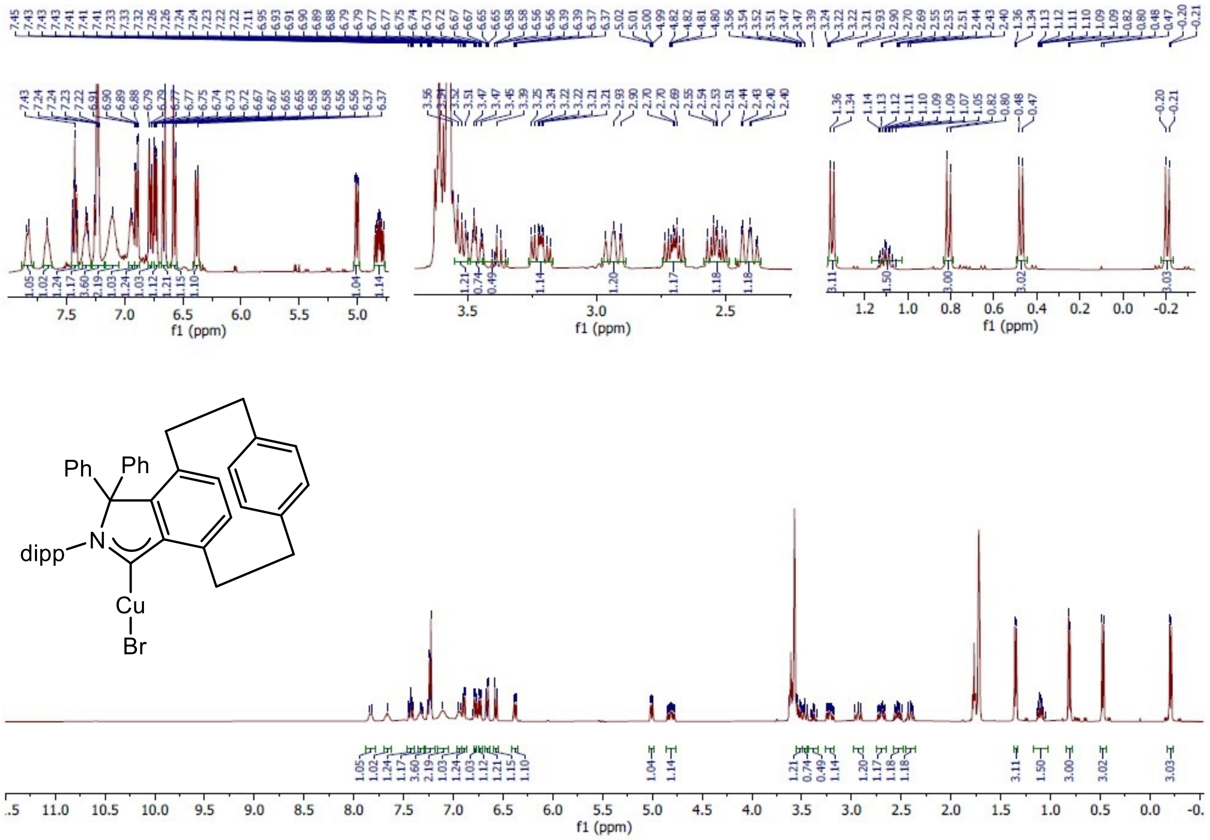

Figure S3.[1]**H-NMR** (400 MHz, THF-d$_8$, 298 K) of **2**.

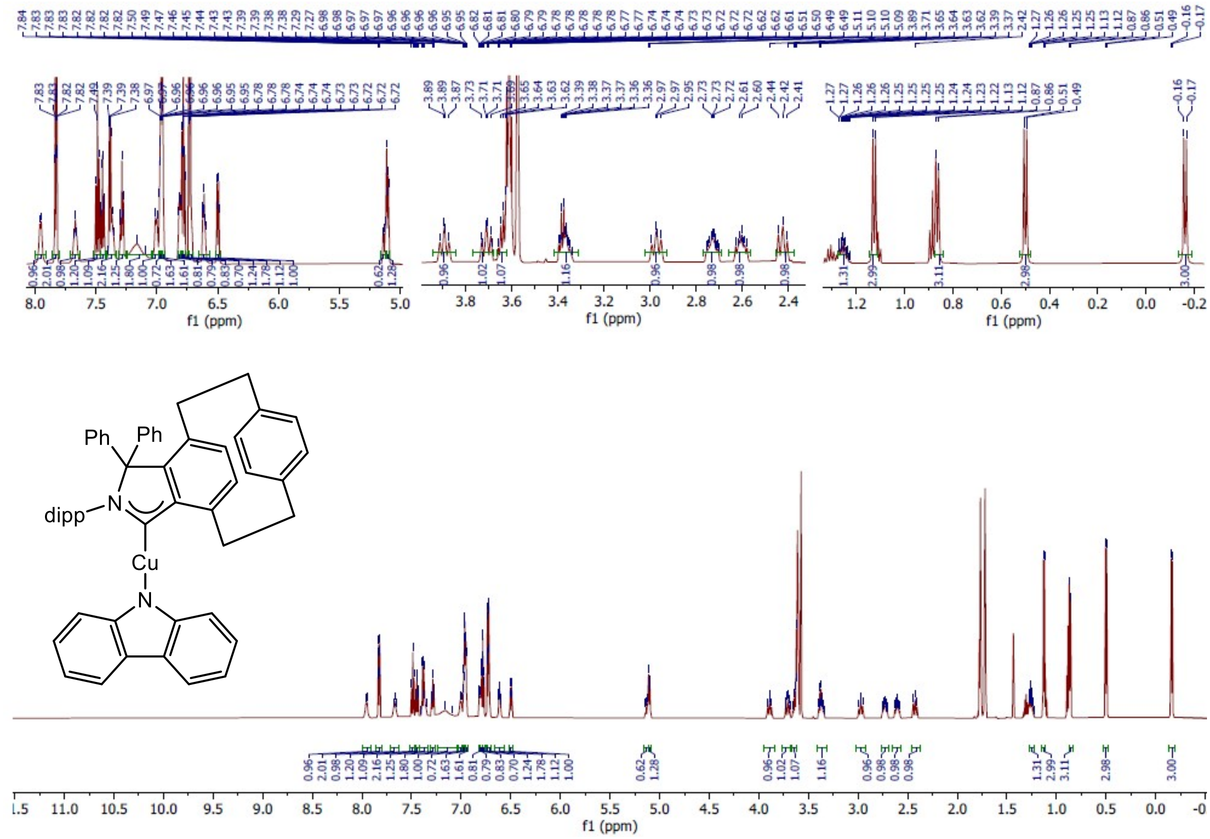

Figure S4. **^{1}H-NMR (**600 MHz, THF-d$_8$, 298 K) of **3**.

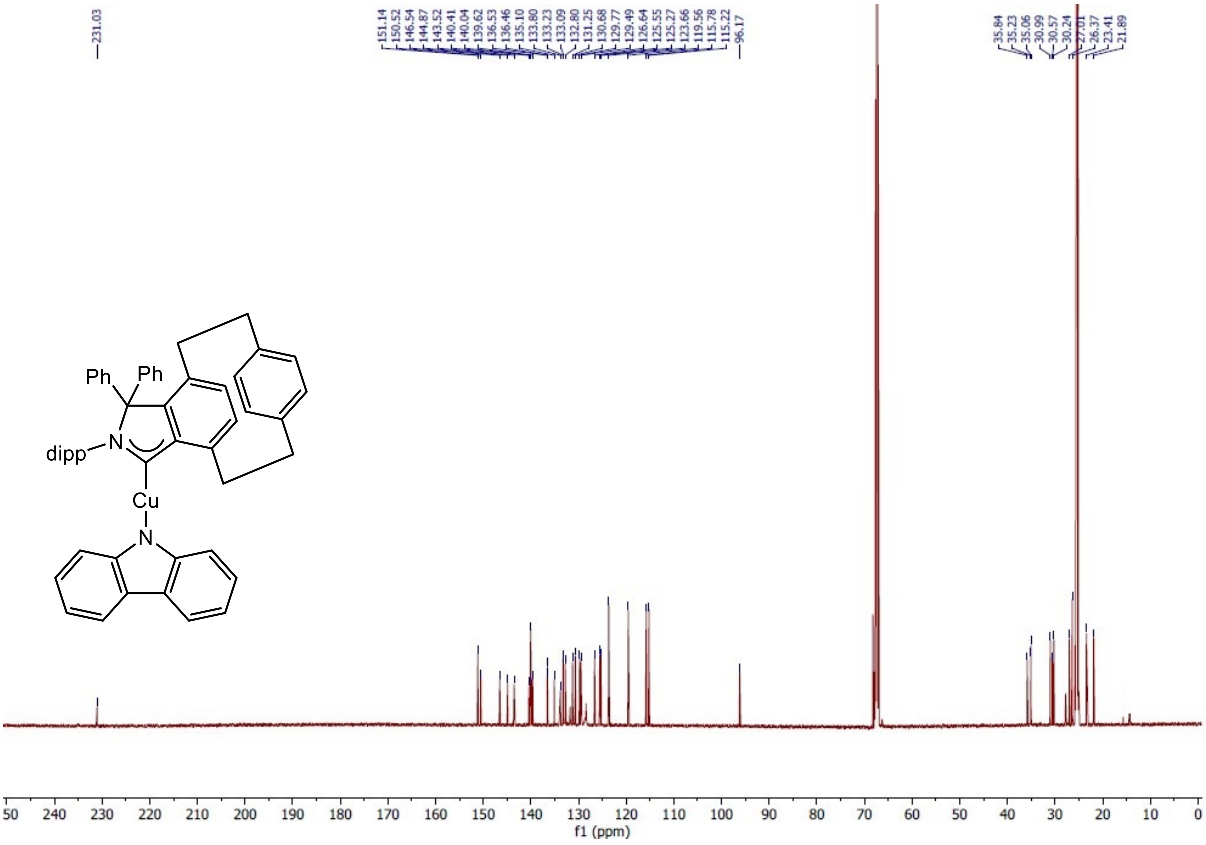

Figure S5. **^{13}C-NMR** (151 MHz, THF-d$_8$, 298 K) of **3**.

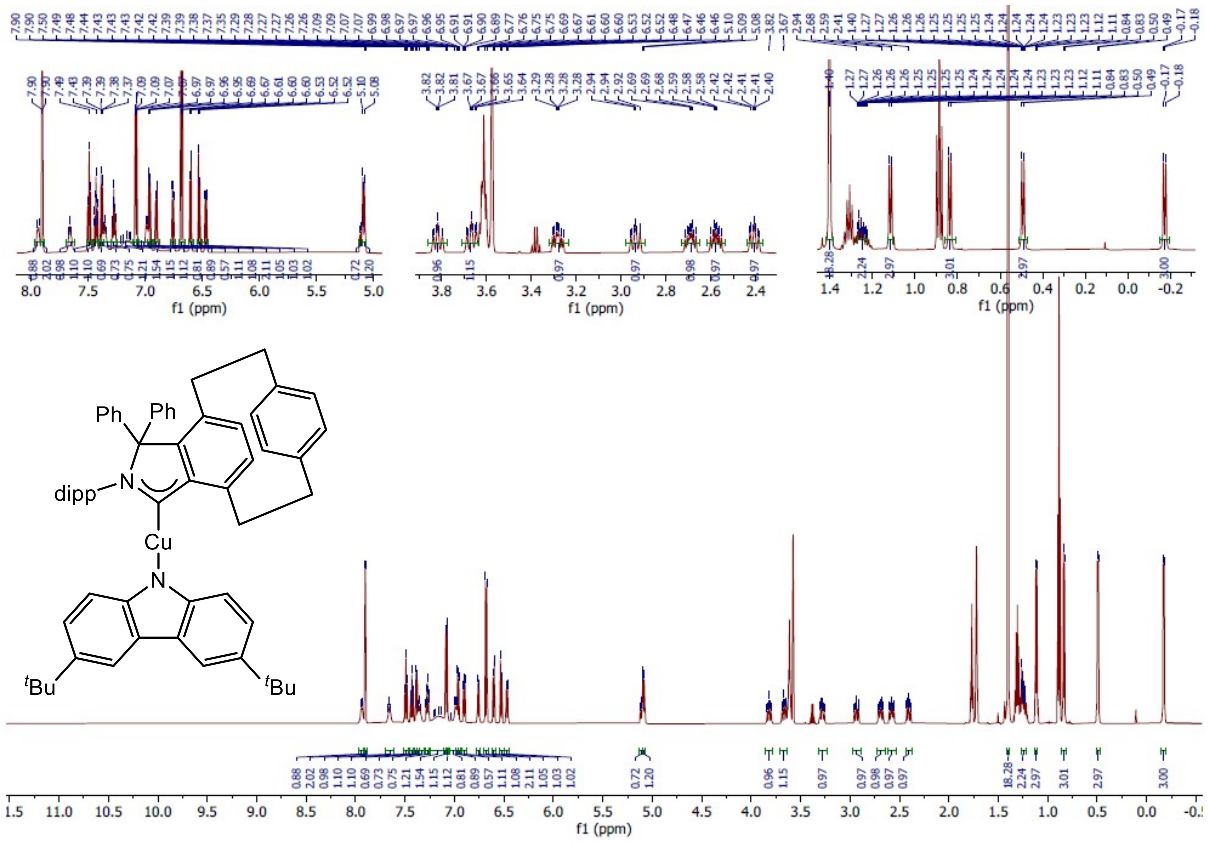

Figure S6. ^{1}H-NMR (600 MHz, THF-d$_8$, 298 K) of **4**.

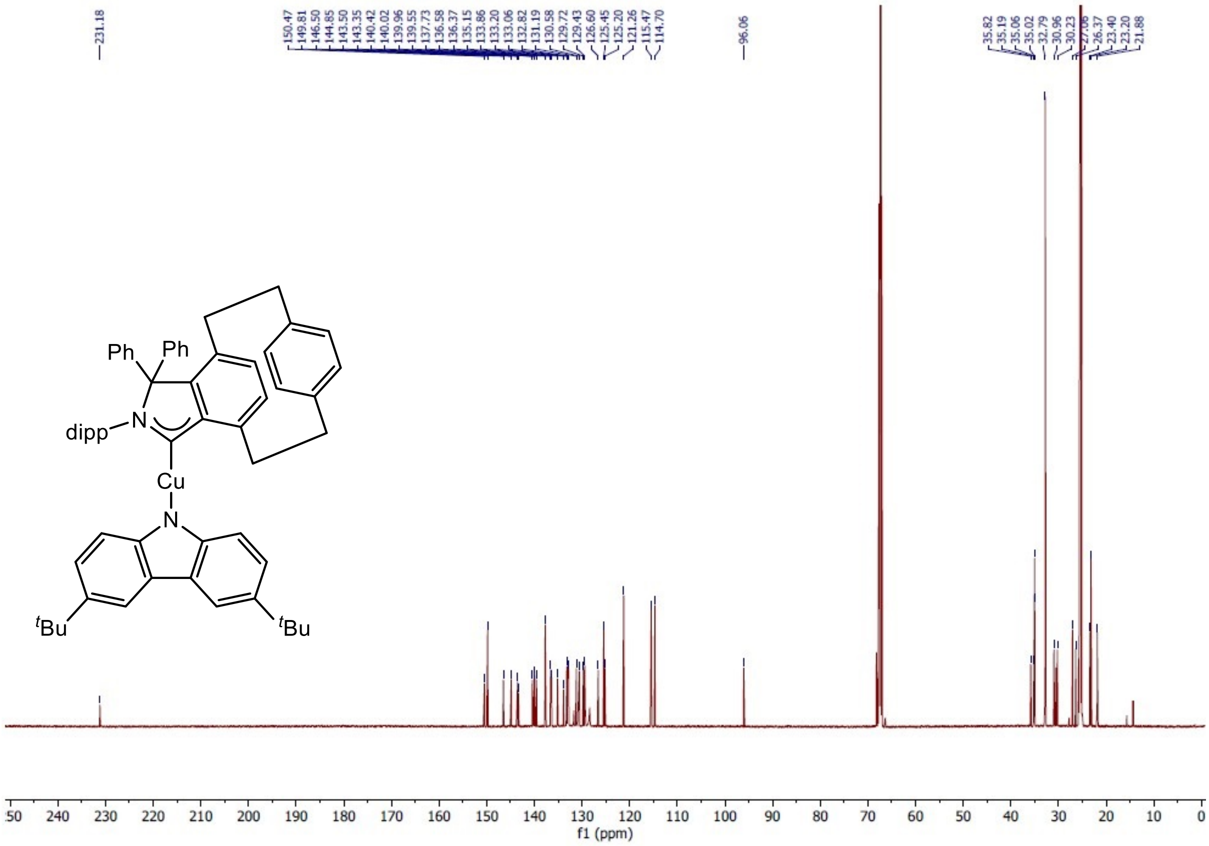

Figure S7. ^{13}C-NMR (151 MHz, THF-d$_8$, 298 K) of **4**.

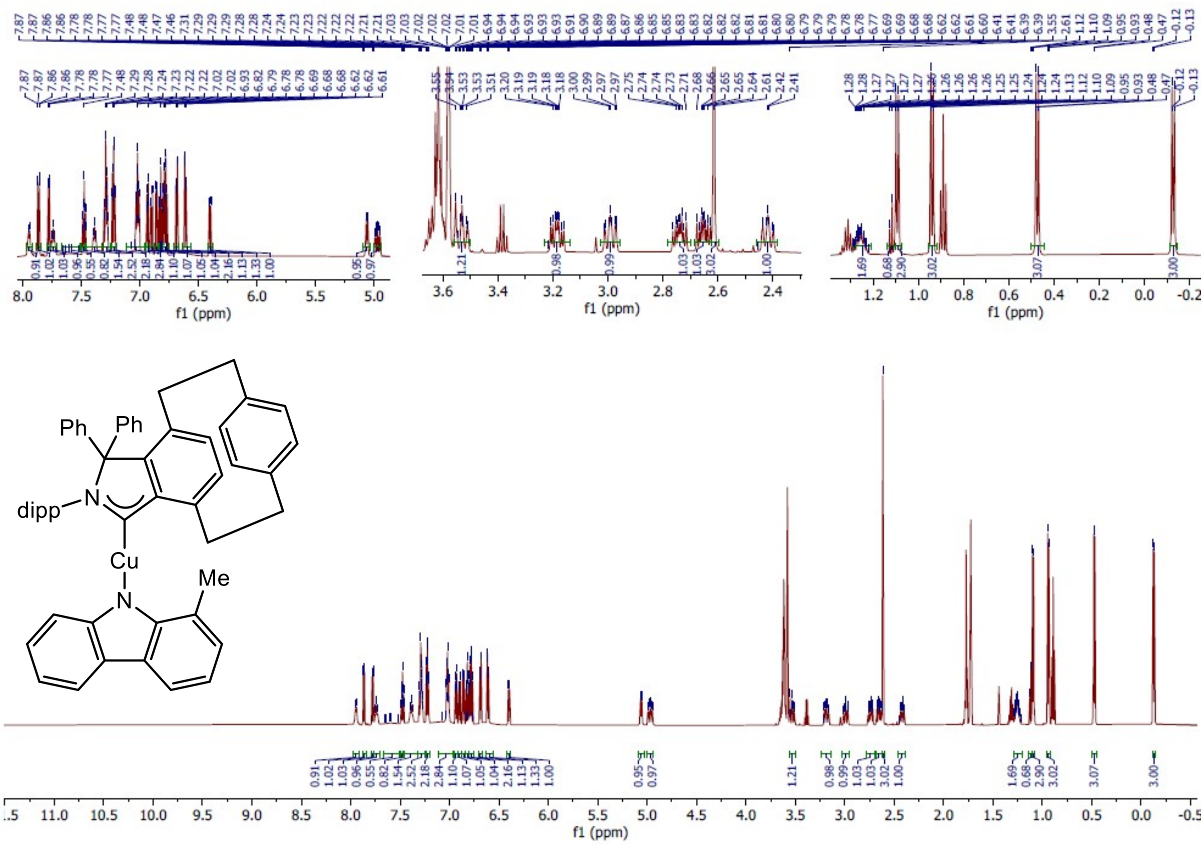

Figure S8. **^{1}H-NMR (**600 MHz, THF-d$_8$, 298 K) of **5**.

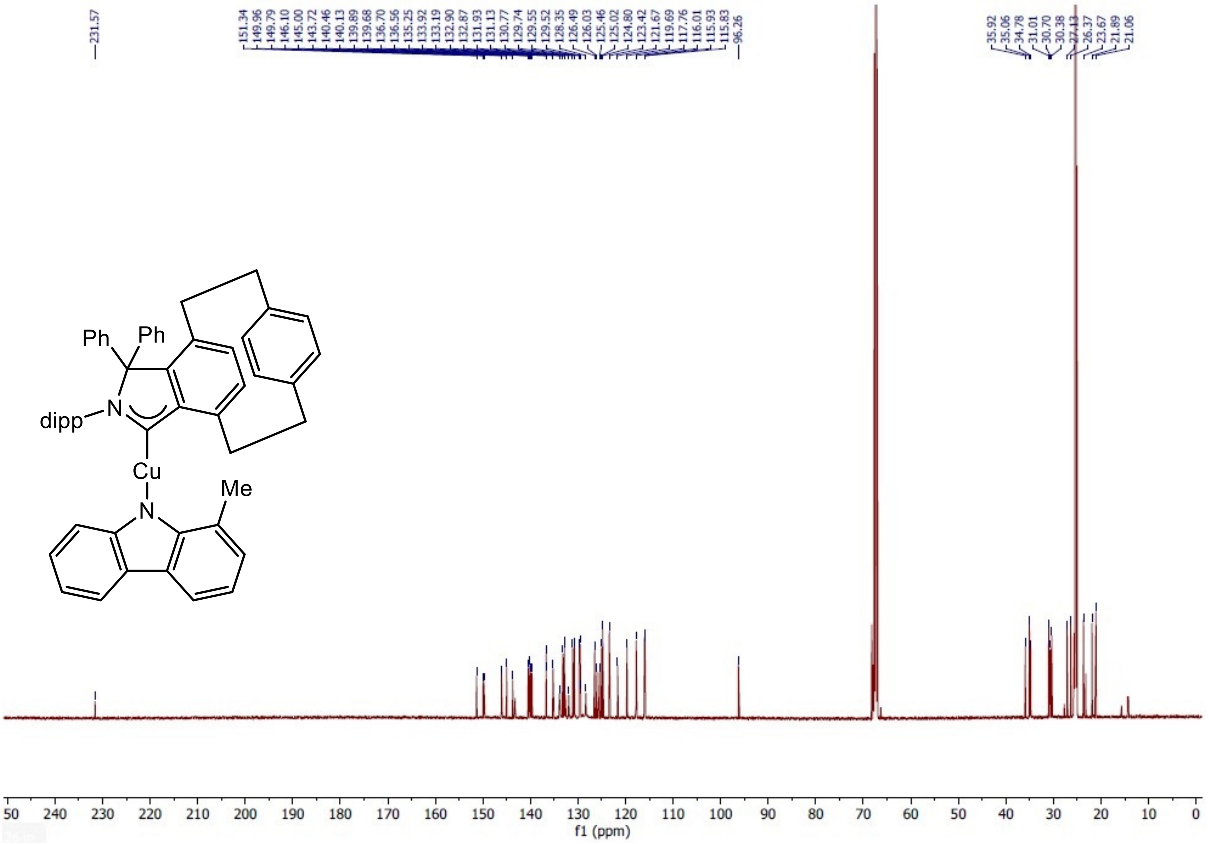

Figure S9. **^{13}C-NMR** (151 MHz, THF-d$_8$, 298 K) of **5**.

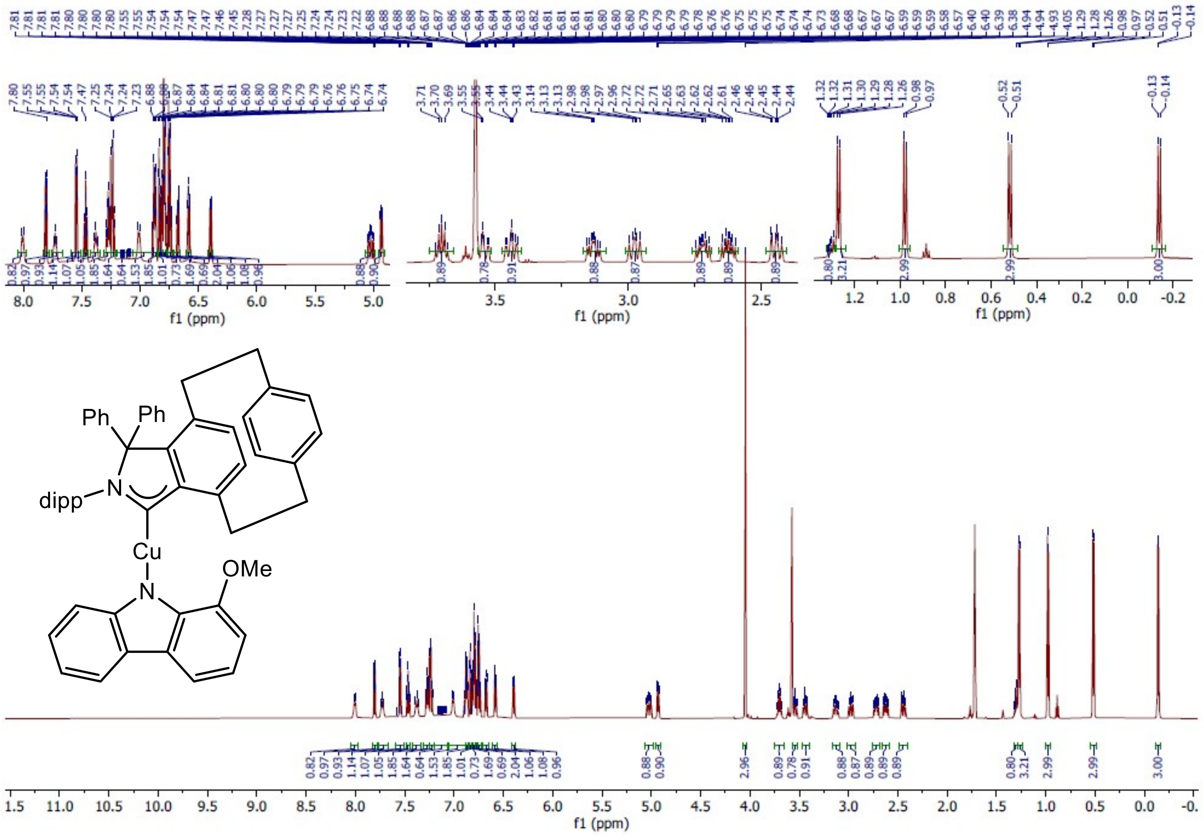

Figure S10. ^{1}**H-NMR (**600 MHz, THF-d$_8$, 298 K) of **6**.

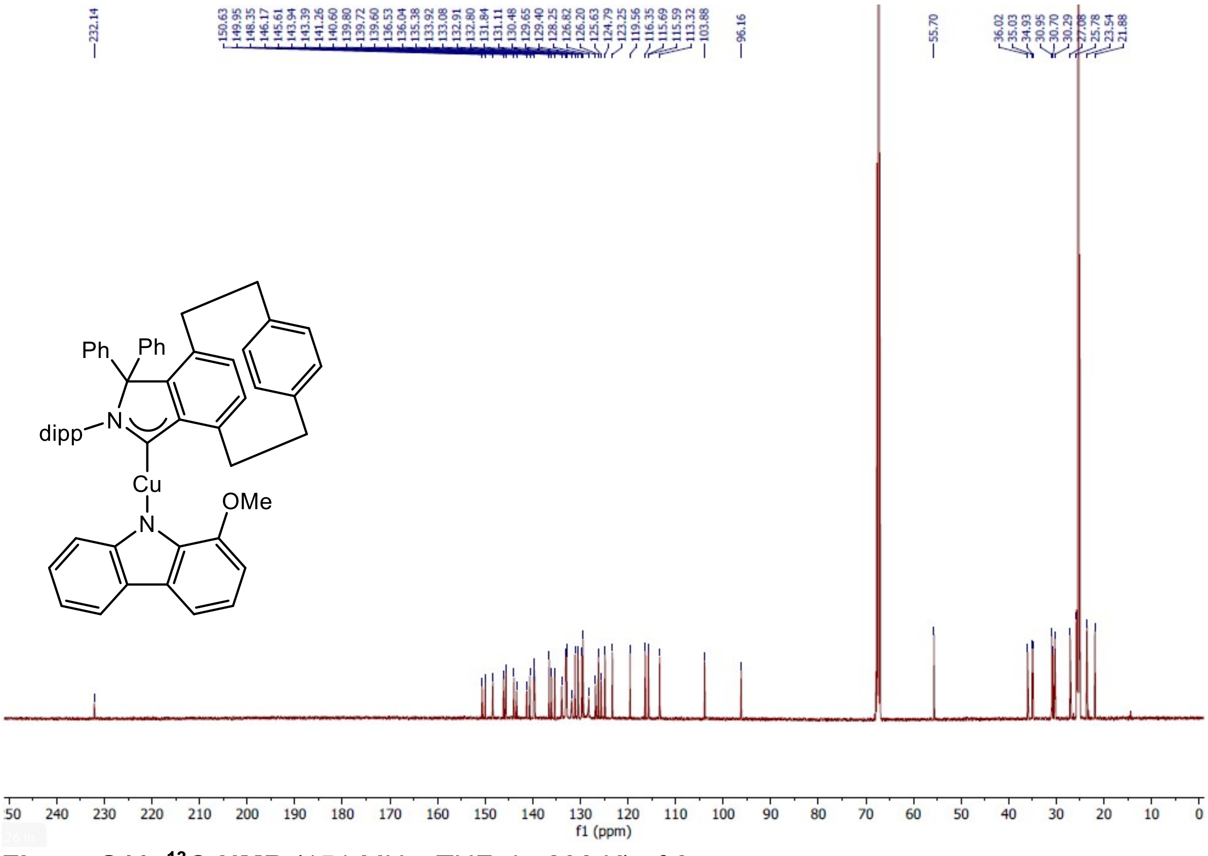

Figure S11. ^{13}**C-NMR** (151 MHz, THF-d$_8$, 298 K) of **6**.

4. X-ray characterization data

General part

X-ray diffraction determination

The crystals of **1–6** were immersed in a film of NVH or perfluoropolyether oil, mounted on a polyimide microloop (MiTeGen) and transferred to a stream of cold nitrogen (Bruker Kryoflex2), and measured at a temperature of 100 or 109 K. The X-ray diffraction data were collected on a Bruker D8 diffractometer with a CMOS Photon 100 and multilayer optics monochromated MoKα (0.71073Å) radiation (INCOATEC microfocus sealed tube). The frames were integrated with the Bruker SAINT software package using a narrow-frame algorithm. The APEX3 v2018.7-0 program package was used for cell refinements and data reductions. The structure was solved using the intrinsic phasing method,[4] refined and visualized with the OLEX2-1.5 program.[5] A semiempirical absorption correction (SADABS) was applied to all data. All non-hydrogen atoms were refined anisotropically. Hydrogen atoms were included in structure factors calculations. All Hydrogen atoms were assigned to idealized geometric positions. The unit cells of **3** and **4** contain disordered solvent molecules which have been treated as a diffuse contribution to the overall scattering without specific atom positions by SQUEEZE/PLATON.[6] The crystallographic details are summarized in Tables S1 and S2. CCDC **2271628**, **2386570-2386573**, and **2386579** numbers contain the supplementary crystallographic data for this paper.

The crystal quality of **2** [Cu(Br)(iPC)] was sufficient to confirm the connectivity.

X-ray Structures

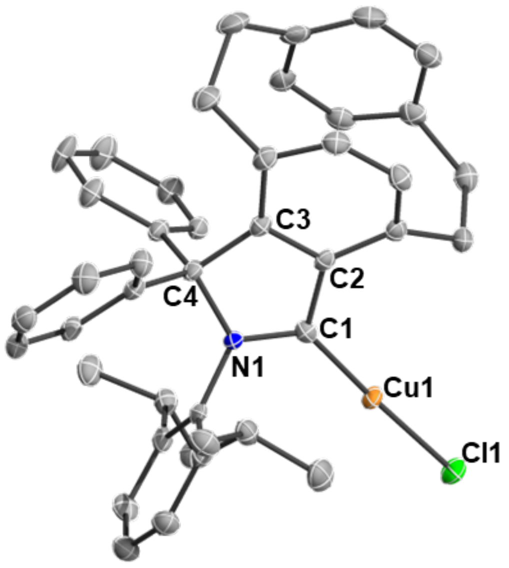

Figure S12. X-ray solid-state structure of **1 [CuCl(iPC)] (S-isomer)**. Thermal ellipsoids were drawn at the 50% probability level; H atoms, solvent molecule and R-isomer have been omitted for clarity. Selected bond lengths (Å) and angles [deg]: C1-N1 1.323(4), C1-Cu1 1.882(2), Cu1-Cl1 2.1236(9), C1-Cu1-Cl1 174.77(6).

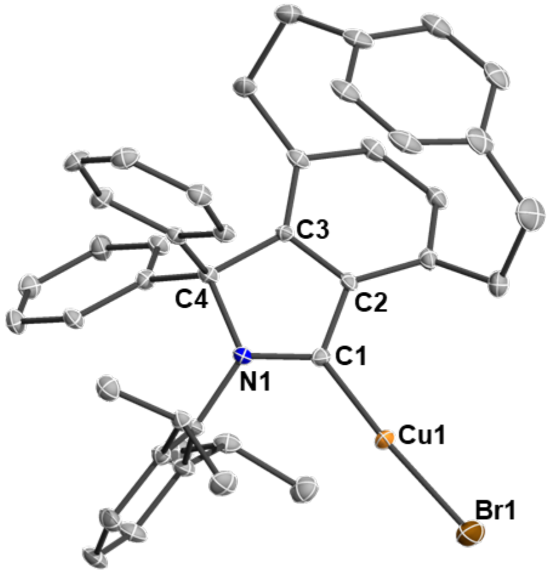

Figure S13. X-ray solid-state structure of **2 [CuBr(iPC)] (R-isomer)**. Thermal ellipsoids were drawn at the 50% probability level; H atoms and S-isomer have been omitted for clarity. Selected bond lengths (Å) and angles [deg]: C1-N1 1.316(4), C1-Cu1 1.897(3), Cu1-Br1 2.2478(6), C1-Cu1-Br1 174.43(9).

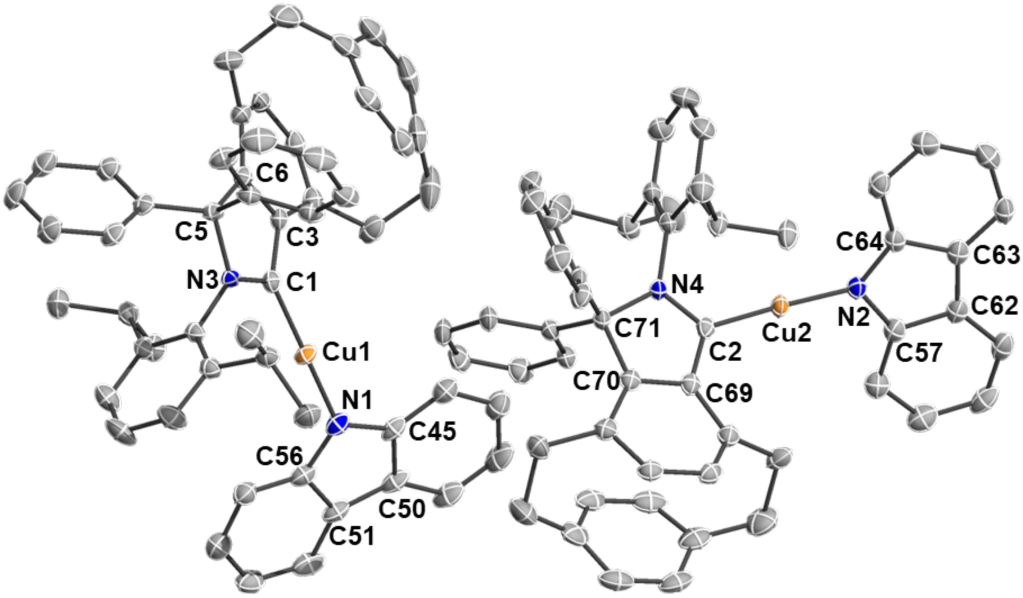

Figure S14. X-ray solid-state structure of **3 [Cu(Cz)(iPC)] (R-isomers)**. Thermal ellipsoids were drawn at the 50% probability level; H atoms, and S-isomers have been omitted for clarity. Selected bond lengths (Å) and angles [deg]: C1-N3 1.330(3), C1-Cu1 1.871(2), Cu1-N1 1.855(2), C1-Cu1-N1 174.87(9), C2-N4 1.325(3), C2-Cu2 1.873(2), Cu2-N2 1.858(2), C2-Cu2-N2 175.13(1).

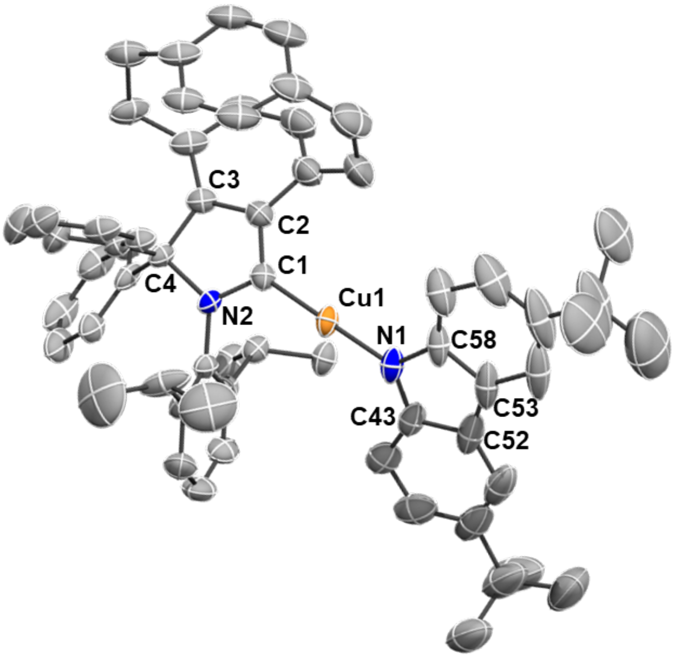

Figure S15. X-ray solid-state structure of **4 [Cu(Cz^{tBu})(iPC)] (R-isomer)**. Thermal ellipsoids were drawn at the 50% probability level; H atoms and S-isomer have been omitted for clarity. Selected bond lengths (Å) and angles [deg]: C1-N2 1.332(3), C1-Cu1 1.874(2), Cu1-N1 1.852(2), C1-Cu1-N1 178.10(9).

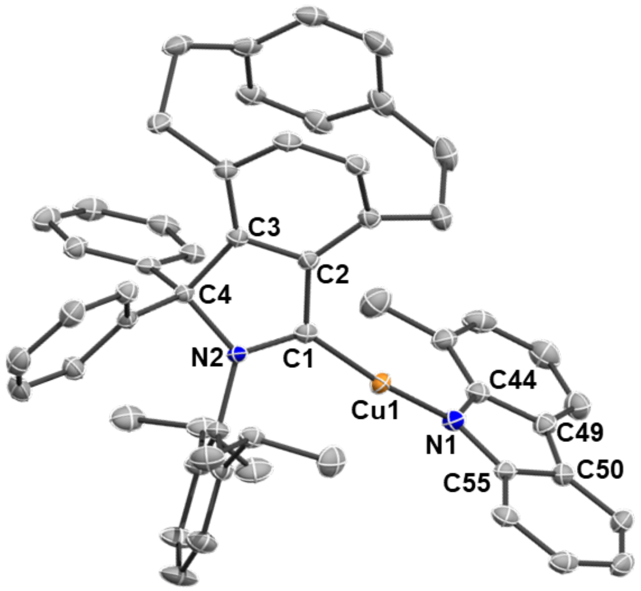

Figure S16. X-ray solid-state structure of **5 [Cu(^{Me}Cz)(iPC)] (R,S-conformer)**. Thermal ellipsoids were drawn at the 50% probability level; H atoms, solvent molecules and S,R-conformer have been omitted for clarity. Selected bond lengths (Å) and angles [deg]: C1-N2 1.325(2), C1-Cu1 1.872(2), Cu1-N1 1.859(2), C1-Cu1-N1 170.34(9).

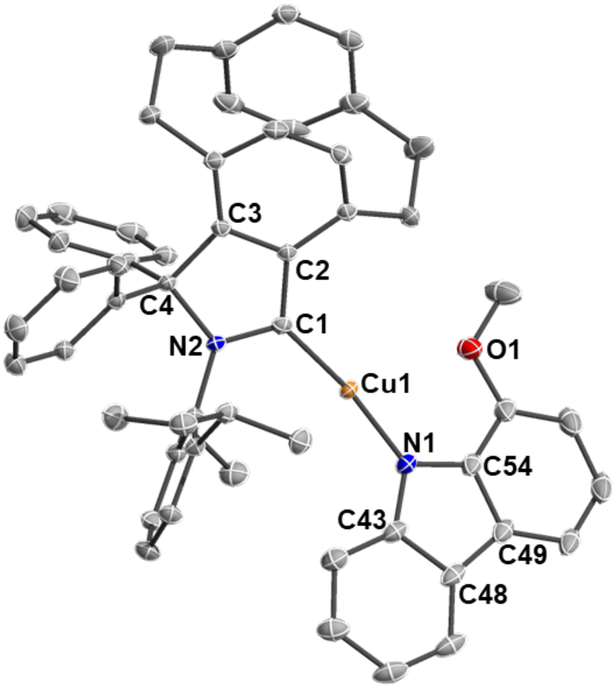

Figure S17. X-ray solid-state structure of **6 [Cu(^{OMe}Cz)(iPC)] (S,R-conformer)**. Thermal ellipsoids were drawn at the 50% probability level; H atoms, solvent molecules and R,S-conformer have been omitted for clarity. Selected bond lengths (Å) and angles [deg]: C1-N2 1.331(2), C1-Cu1 1.878(1), Cu1-N1 1.867(1), C1-Cu1-N1 171.27(7).

Table S1. Crystal data and structure refinement for **1, 3–6**.

Identification code	**1** [CuCl(iPC)]	**3** [Cu(Cz)(iPC)]	**4** [Cu(Cz^{tBu})(iPC)]	**5** [Cu(^{Me}Cz)(iPC)]	**6** [Cu(^{OMe}Cz)(iPC)]
CCDC number	**2271628**	**2386570**	**2386571**	**2386572**	**2386573**
Empirical formula	$C_{46}H_{49}ClCuNO$	$C_{54}H_{49}CuN_2$	$C_{62}H_{65}CuN_2$	$C_{59}H_{59}Cl_2CuN_2$	$C_{62}H_{59}CuN_2O$
Formula weight	730.85	789.49	901.70	930.52	911.65
Temperature [K]	100.00				
Crystal system	triclinic	triclinic	monoclinic	triclinic	monoclinic
Space group	P-1	P-1	$P2_1/n$	P-1	C2/c
a [Å]	9.770(2)	14.5590(5)	19.7377(7)	12.6764(4)	20.6394(6)
b [Å]	15.193(4)	16.8310(6)	17.5008(5)	14.4116(5)	14.7201(4)
c [Å]	15.794(4)	19.7336(7)	20.3020(7)	15.3308(5)	31.6213(11)
α [°]	63.057(7)	89.1390(10)	90	69.3780(10)	90
β [°]	79.152(8)	84.7080(10)	102.2620(10)	74.5390(10)	97.1470(10)
γ [°]	86.052(8)	79.8220(10)	90	64.5130(10)	90
Volume [Å^3]	2052.4(9)	4739.2(3)	6852.8(4)	2344.15(14)	9532.4(5)
Z	2	4	4	2	8
ρ_{calc} [g/cm^3]	1.183	1.107	0.874	1.318	1.270
μ [mm^{-1}]	0.630	0.496	0.349	0.622	0.504
F(000)	772.0	1664.0	1920.0	980.0	3856.0
Crystal size [mm^3]	0.2 × 0.05 × 0.05	0.177 × 0.12 × 0.101	0.092 × 0.078 × 0.034	0.201 × 0.097 × 0.054	0.144 × 0.111 × 0.08
Radiation type	MoKα (λ = 0.71073)				
2Θ range for data collection [°]	5.38 to 50	5.244 to 53.554	4.654 to 60.094	5.642 to 60.178	4.132 to 60.04
Index ranges	-11 ≤ h ≤ 11, -18 ≤ k ≤ 18, -18 ≤ l ≤ 18	-18 ≤ h ≤ 18, -21 ≤ k ≤ 21, -24 ≤ l ≤ 24	-27 ≤ h ≤ 27, -21 ≤ k ≤ 24, -28 ≤ l ≤ 28	-17 ≤ h ≤ 17, -20 ≤ k ≤ 20, -21 ≤ l ≤ 21	-29 ≤ h ≤ 28, -20 ≤ k ≤ 20, -44 ≤ l ≤ 37
Reflections collected	47616	96378	122818	67750	49424
Independent reflections	7214 [R_{int} = 0.1267, R_{sigma} = 0.0739]	20172 [R_{int} = 0.0893, R_{sigma} = 0.0637]	19975 [R_{int} = 0.0710, R_{sigma} = 0.0483]	13722 [R_{int} = 0.0792, R_{sigma} = 0.0635]	13861 [R_{int} = 0.0311, R_{sigma} = 0.0316]
Data/restraints/parameters	7214/6/455	20172/0/1035	19975/202/621	13722/138/597	13861/308/665
Goodness-of-fit on F^2 (a)	1.025	1.036	1.041	1.024	1.055
Final R indexes [I>=2σ (I)] (b)	R_1 = 0.0576, wR_2 = 0.1511	R_1 = 0.0456, wR_2 = 0.1198	R_1 = 0.0724, wR_2 = 0.1919	R_1 = 0.0508, wR_2 = 0.1180	R_1 = 0.0449, wR_2 = 0.1191
Final R indexes [all data] (b)	R_1 = 0.0769, wR_2 = 0.1670	R_1 = 0.0702, wR_2 = 0.1310	R_1 = 0.0986, wR_2 = 0.2092	R_1 = 0.0803, wR_2 = 0.1318	R_1 = 0.0527, wR_2 = 0.1241
Largest diff. peak/hole [e/Å^{-3}]	0.98/-0.62	0.54/-0.38	0.86/-0.54	1.33/-1.00	1.52/-0.44

a) GooF = S = $[[\sum w(F_o^2-F_c^2)^2]/(m-n)]^{1/2}$, where m = number of reflexes and n = number of parameters; (b) $R_1 = \sum||F_o|-|F_c||/\sum|F_o|$; $wR_2 = [\sum[w(F_o^2-F_c^2)^2/\sum[(wF_o^2)^2]]^{1/2}$; $w = 1/[\sigma^2(F_o^2)+(aP)^2+bP]$, where $P = (F_o^2+2F_c^2)/3$

Table S2. Crystal data and structure refinement for **2**.

Identification code	**2** [CuBr(iPC)]
CCDC number	**2386579**
Empirical formula	$C_{42}H_{41}BrCuN$
Formula weight	703.21
Temperature [K]	109.00
Crystal system	monoclinic
Space group	$P2_1/n$
a [Å]<br>b [Å]<br>c [Å]<br>α [°]<br>β [°]<br>γ [°]	12.3719(6)<br>14.6658(6)<br>18.0296(9)<br>90<br>90.052(2)<br>90
Volume [Å^3]	3271.4(3)
Z	4
ρ_{calc} [g/cm^3]	1.428
μ [mm^{-1}]	1.921
F(000)	1456.0
Crystal size [mm^3]	0.302 × 0.084 × 0.072
Radiation type	MoKα (λ = 0.71073)
2Θ range for data collection [°]	4.308 to 60.116
Index ranges	-17 ≤ h ≤ 17, -20 ≤ k ≤ 20, -25 ≤ l ≤ 25
Reflections collected	54326
Independent reflections	7113 [R_{int} = 0.0796, R_{sigma} = 0.0687]
Data/restraints/parameters	7113/0/410
Goodness-of-fit on F^2 (a)	1.084
Final R indexes [I>=2σ (I)] (b)	R_1 = 0.0601, wR_2 = 0.1657
Final R indexes [all data] (b)	R_1 = 0.0835, wR_2 = 0.1931
Largest diff. peak/hole [e/Å^{-3}]	0.70/-1.69

a) GooF = S = $[[\sum w(F_o^2-F_c^2)^2]/(m-n)]^{1/2}$, where m = number of reflexes and n = number of parameters; (b) $R_1 = \sum||F_o|-|F_c||/\sum|F_o|$; $wR_2 = [\sum[w(F_o^2-F_c^2)^2/\sum[(wF_o^2)^2]]^{1/2}$; $w = 1/[\sigma^2(F_o^2)+(aP)^2+bP]$, where $P = (F_o^2+2F_c^2)/3$

5. Structural Details

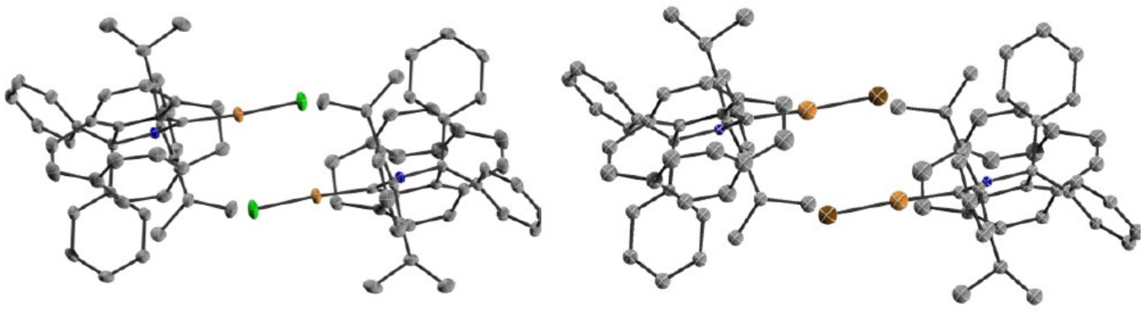

Figure S18. Aggregation of the complexes rac-[CuCl(iPC)] **1** (left) and rac-[CuBr(iPC)] **2** (right) in a single crystal at 100 K. Hydrogen atoms are omitted for clarity.

Table S3. Selected bond distances (in Å) and bond angles (in °) compounds **1** and **2** (X = Cl and Br).

	Cu-$C_{carbene}$	Cu-X	C1-N1	$C_{carbene}$-Cu-X	X-Cu-X'	Cu-Cu'	Cu-X'
**1**	1.887(2)	2.1233(9)	1.321(4)	174.77(10)	94.82(4)	3.7551(9)	3.2812(13)
**2**[a]	1.897(3)	2.2478(6)	1.316(4)	174.43(9)	90.70	4.18(1)	5.06(8)

[a]Approximate values are given for complex **2** due to poor data quality of crystal structure.

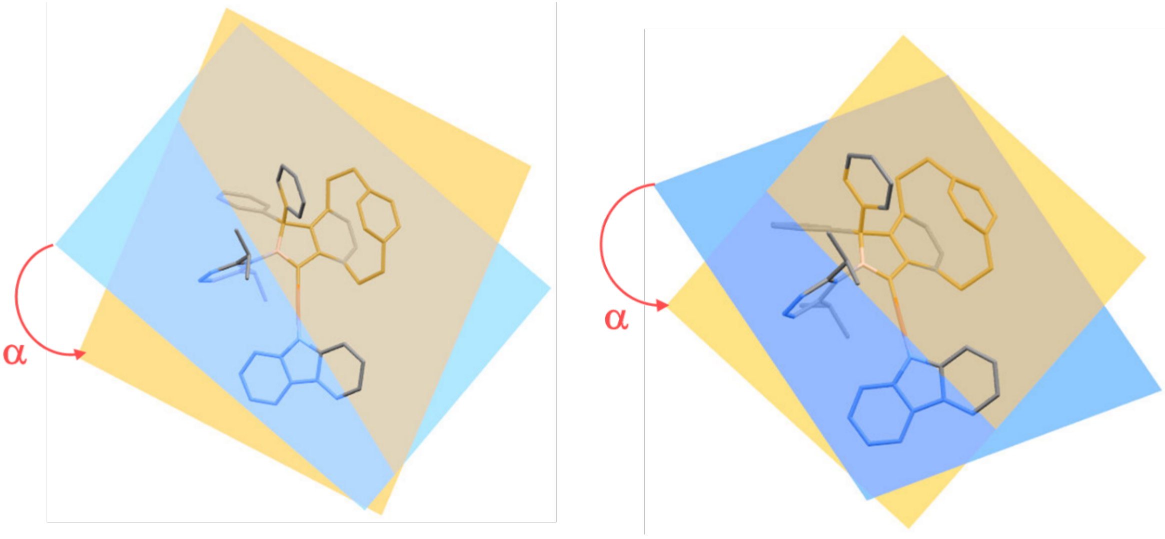

Figure S19. Dihedral angle in **3**. Right : Dihedral angle α = 46.4(2) ° between blue plane (mean: N3C1C3) and yellow plane (mean: N1C45C56). Left : Dihedral angle α = 52.2(4) ° between blue plane (mean: N4C2C69) and yellow plane (mean: N2C57C64).

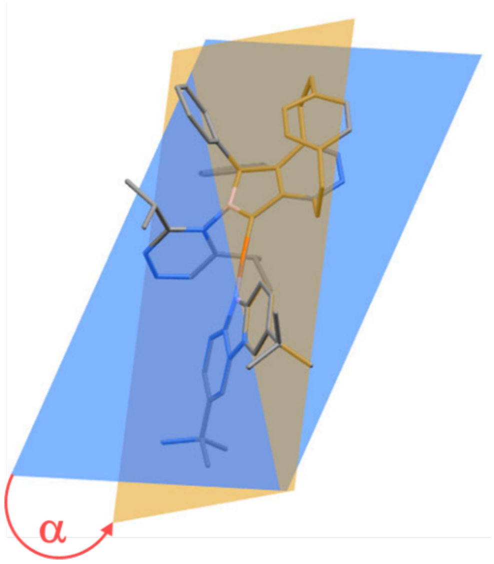

Figure S20. Dihedral angle α = 42.9(5) ° between blue plane (mean: N2C1C2) and yellow plane (mean: N1C43C58) in **4**.

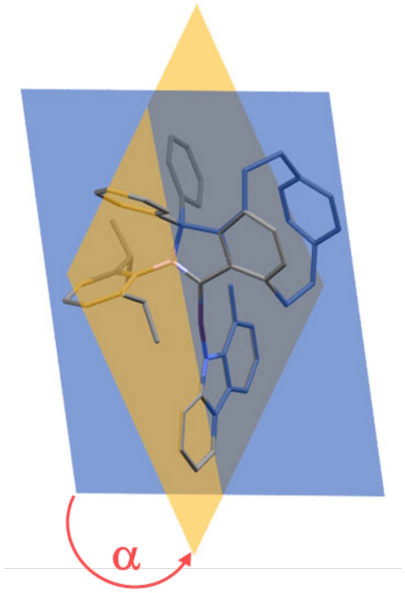

Figure S21. Dihedral angle α = 77.0(5) ° between blue plane (mean: N2C1C2) and yellow plane (mean: N1C44C55) in **5**.

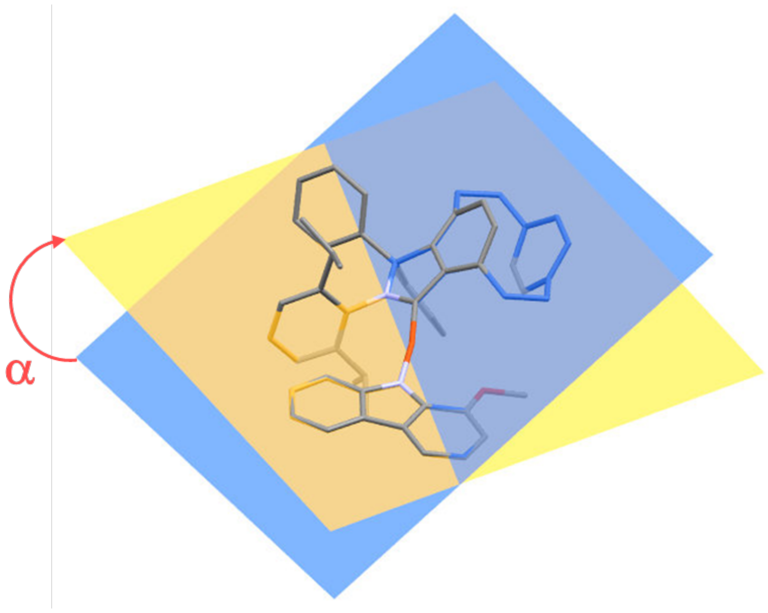

Figure S22. Dihedral angle α = 32.3(8) ° between blue plane (mean: N2C1C2) and yellow plane (mean: N1C43C54) in **6**.

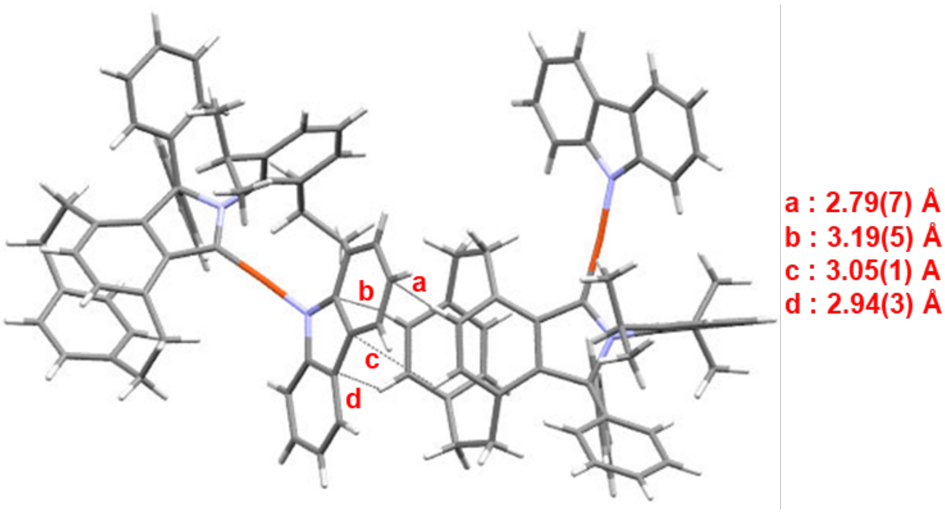

Figure S23. Intermolecular C–H···π interactions between the Cz and iPC ligands in **3**.

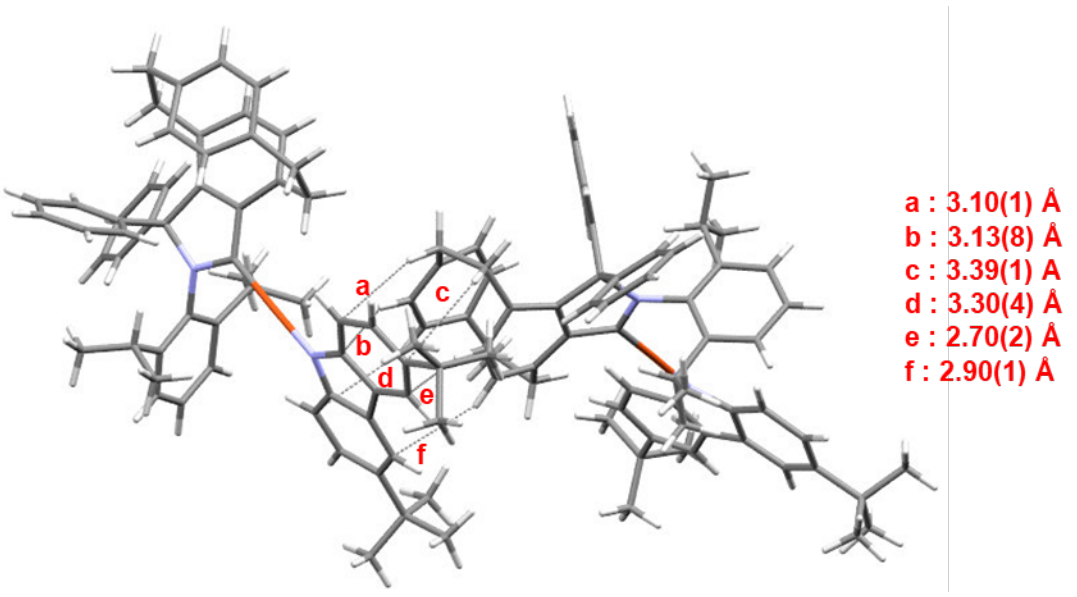

Figure S24. Intermolecular C–H···π interactions between the Cz^{tBu} and iPC ligands in **4**.

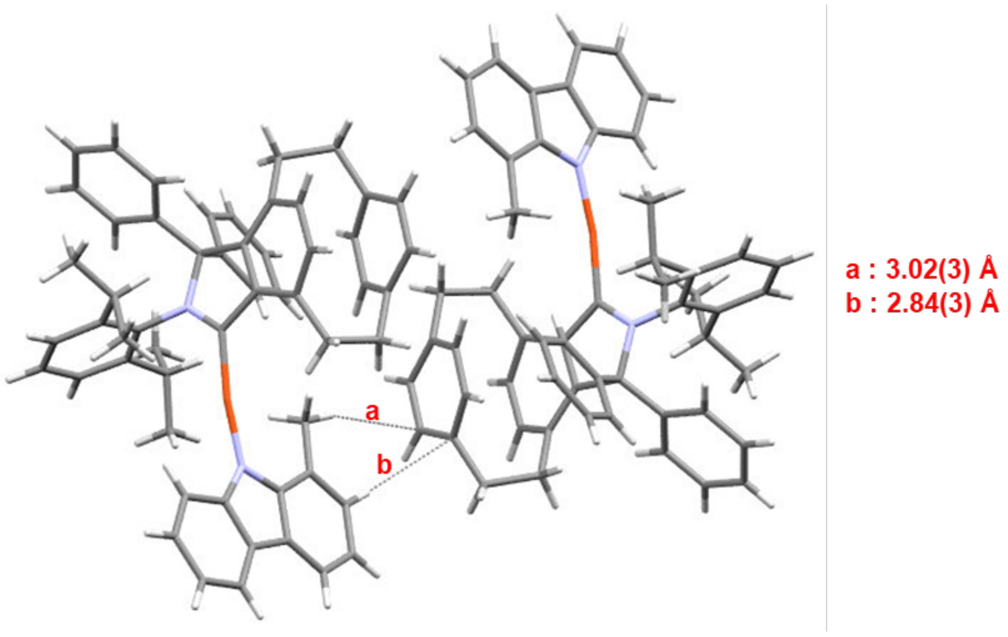

Figure S25. Intermolecular C–H···π interactions between the ^{Me}Cz and iPC ligands in **5**.

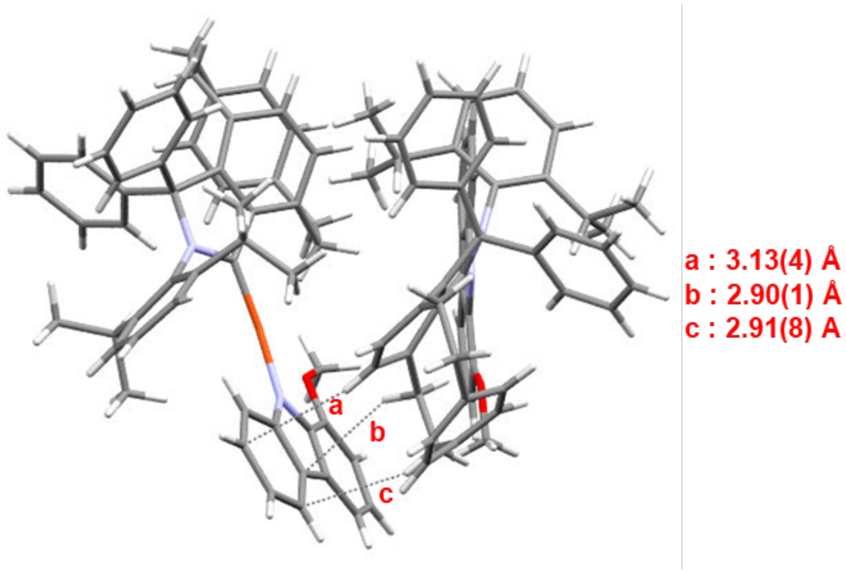

Figure S26. Intermolecular C–H···π interactions between the ^{OMe}Cz and iPC ligands in **6**.

6. Photophysical Measurements

All photophysical measurements were performed in dry and deaerated solutions. The solid-state measurements were performed either in single-crystalline form (sample denoted as 'crystals') or, to remove co-crystallized molecules of solvent, single-crystals were ground and dried under vacuum for 24 h (sample denoted as 'solid'). Optical absorption spectroscopy was performed using an Agilent Cary 5000 spectrophotometer using standard 1 cm path length quartz cells. Excitation and emission spectra were recorded on an Edinburgh Instrument FLS1000 spectrometer, equipped with a 450 W Xenon arc lamp, double monochromators for the excitation and emission pathways, and a red-sensitive photomultiplier (PMT-980) as a detector. For NIR measurements liquid N_2-cooled PMT-1400, range up to 1400 nm was used. The excitation and emission spectra were corrected using the standard corrections supplied by the manufacturer for the excitation source‘s spectral power and the detector‘s sensitivity. Quantum yields in solution were measured using an FLS1000 spectrometer equipped with an integrating sphere (N-M01), or Quantaurus-QY Absolute PL quantum yield spectrometer (C11347- 11 Series: Standard type) from Hamamatsu and the quantum yield of solid samples were measured using an integrating cryosphere (Microstat N2) from Oxford Instruments. The luminescence lifetimes were measured using a μF2 pulsed 60 W Xenon microsecond flashlamp, with a repetition rate of 100 Hz, and a time-correlated single photon counting (TCSPC) module or EPLED (365 nm with 1.7 μW), with 50 ns pulse width and an TCSPC module, depending on the time range and a multichannel scaling (MCS) module or VPLED (383.8 nm with 1.1 mW or 449.6 nm with 37 mW), with 48.1 ns or 59.8 ns minimum pulse width respectively and an MCS module, depending on the time range. The emission was collected at a right angle to the excitation source.

7. UV-Visible Spectroscopy

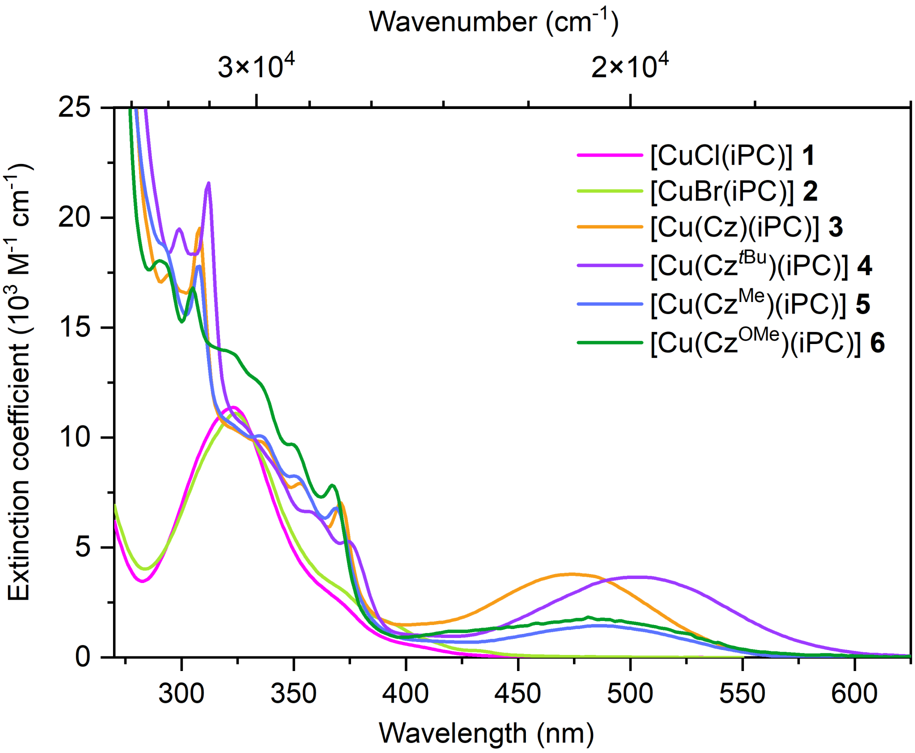

Figure S27. UV-Vis absorption spectra of compounds **1-6** in THF.

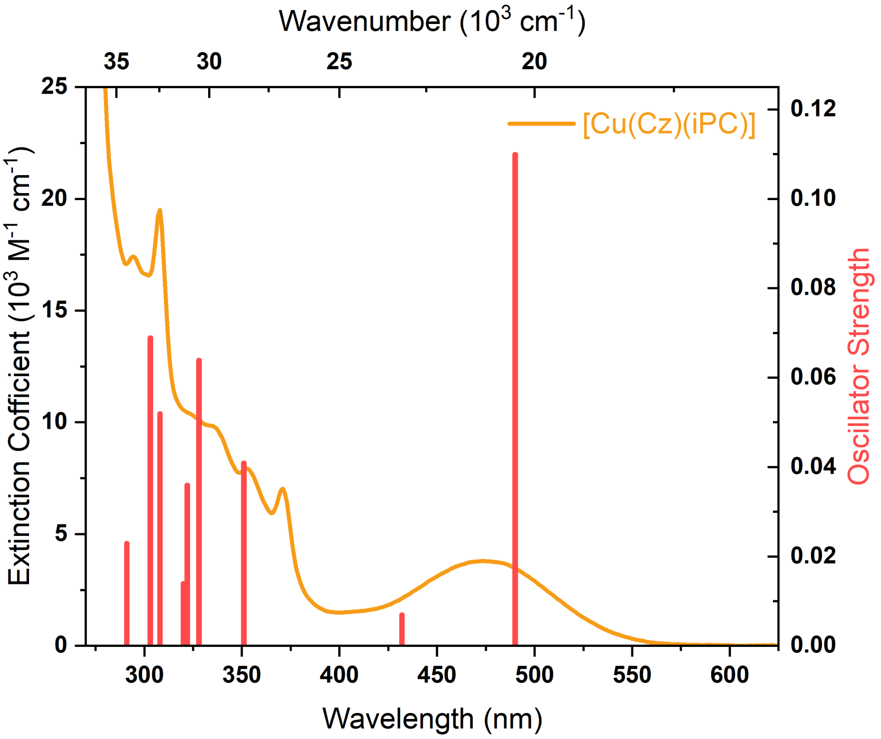

Figure S28. UV-Vis absorption spectra in THF and TD-DFT calculated oscillator strength of **3**.

8. Emission Studies

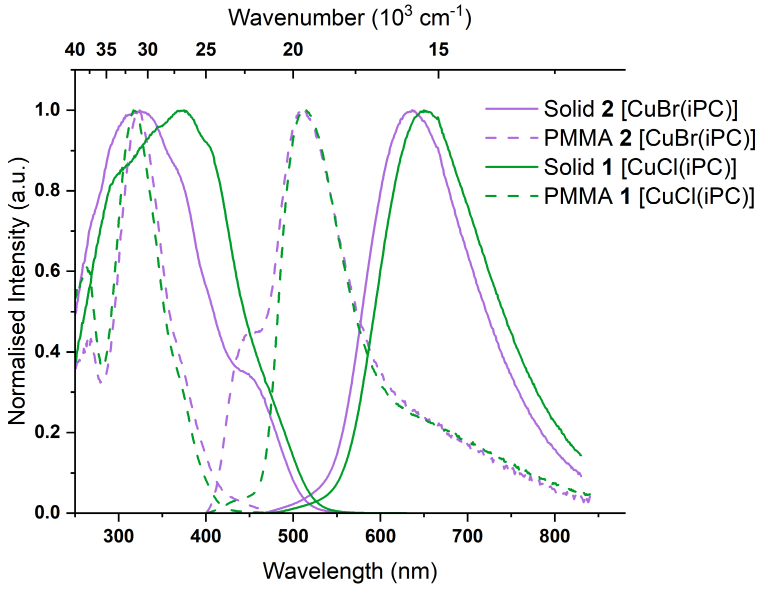

Figure S29. Normalized excitation and emission spectra of compounds **1** (green) and **2** (purple) at room temperature. Solid lines for solid state (ground) measurements and dash lines for measurements in PMMA matrix (1 wt% of the compound in DCM) .

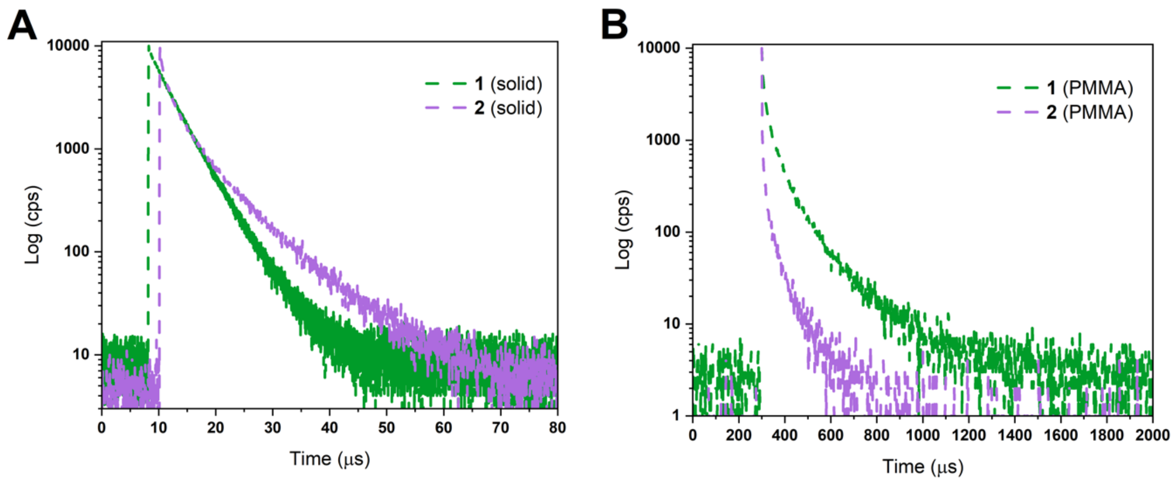

Figure S30. Emission decays of compound **1** (green) and **2** (purple) at room temperature (**A**: Solid state, **B**: PMMA)**.**

Table S4. Selected photophysical data of compound **1** and **2** at RT.

	Medium	λ_{max} (nm)	τ (μs)[a]	φ	k_r (10^5 s^{-1})[b]
**1**	Solid (ground)	650	2.8 (38.7)/ 4.8 (61.3)	0.13	0.31
	PMMA	510	13 (50.0)/ 50 (42.3)/ 159 (8.7)	0.03	0.0062
**2**	Solid (ground)	635	1.2 (40.4)/ 4.4 (42.6)/ 9.9 (16.9)	0.03	0.064
	PMMA	510	14 (78.7)/ 73 (21.3)	0.02	0.0089

(a) For lifetimes fitted with two or multi exponentials, the pre-exponential factors B are given in parentheses. (b) k_r was calculated using amplitude-weighted averaged lifetimes.

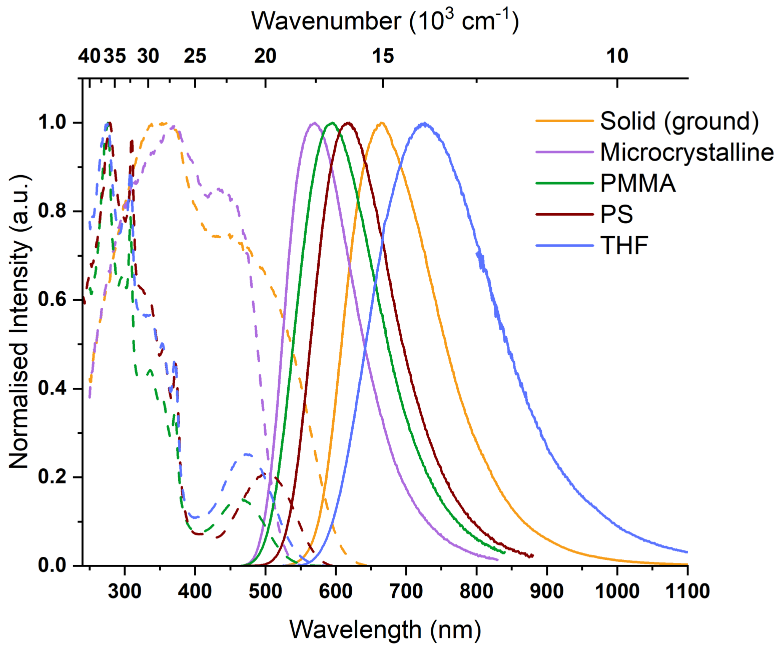

Figure S31. Normalized excitation and emission spectra of compound **3** in solid state (orange), microcrystalline state (purple), PMMA matrix (green; 1 wt% of the compound in DCM), PS matrix (brown; 1 wt% of the compound in DCM) and THF (blue) at room temperature. Solid lines for emission spectra and dash lines for excitation spectra.

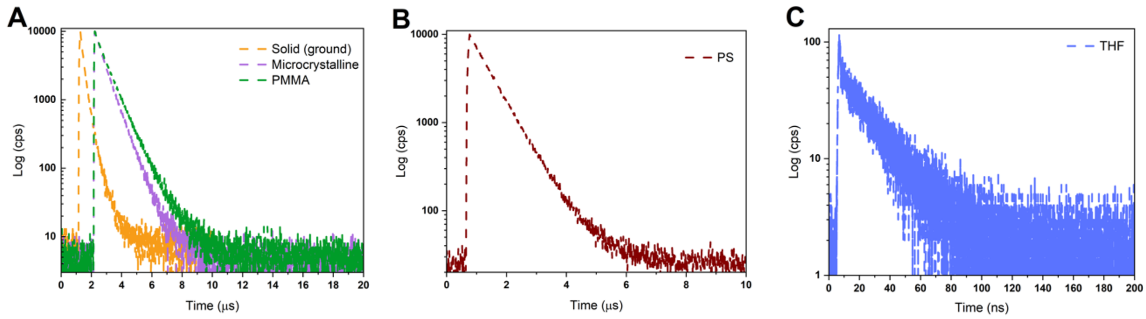

Figure S32. Emission decays of compound **3** at room temperature in solid state (ground; orange), microcrystalline state (purple), PMMA matrix (green), PS matrix (brown) and THF (blue).

Table S5. Selected photophysical data of compound **3** at RT.

	Medium	λ_{max} (nm)	τ (µs)[a]	φ	k_r (10^5 s^{-1})[b]
**3**	Solid(ground)	665	0.18 (66.3)/ 0.33 (33.7)	0.22	9.8
	Microcrystalline	570	0.46 (62.1)/ 0.77 (37.9)	0.80	14
	PMMA	595	0.56 (59.4)/ 0.96 (40.6)	0.58	8.1
	PS	615	0.69 (98.7)/ 1.62 (1.2)	0.62	8.7
	THF	725	0.021	0.01	4.8

(a) For lifetimes fitted with two exponentials, the pre-exponential factors B are given in parentheses. (b) k_r was calculated using amplitude-weighted averaged lifetimes.

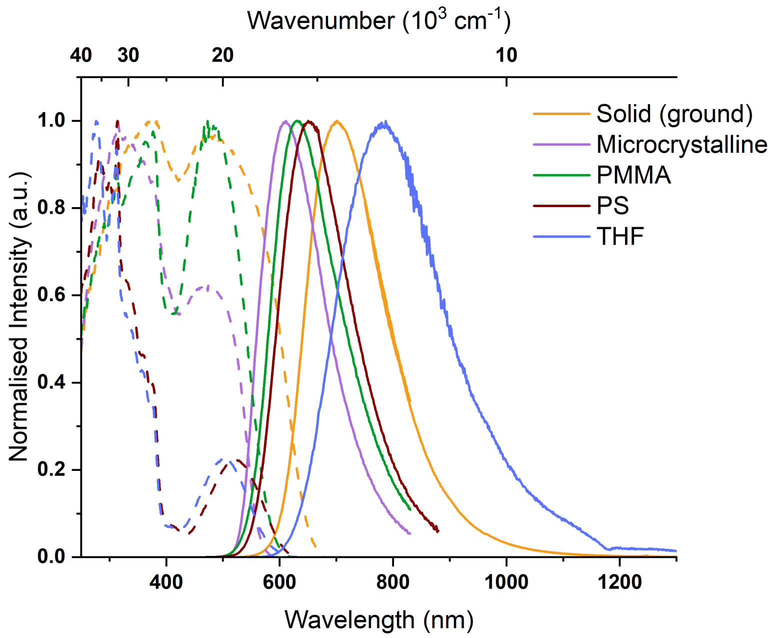

Figure S33. Normalized excitation and emission spectra of compound **4** in solid state (ground; orange), microcrystalline state (purple), PMMA matrix (green; 1 wt% of the compound in DCM), PS matrix (brown; 1 wt% of the compound in DCM) and THF (blue) at room temperature. Solid lines for emission spectra and dash lines for excitation spectra.

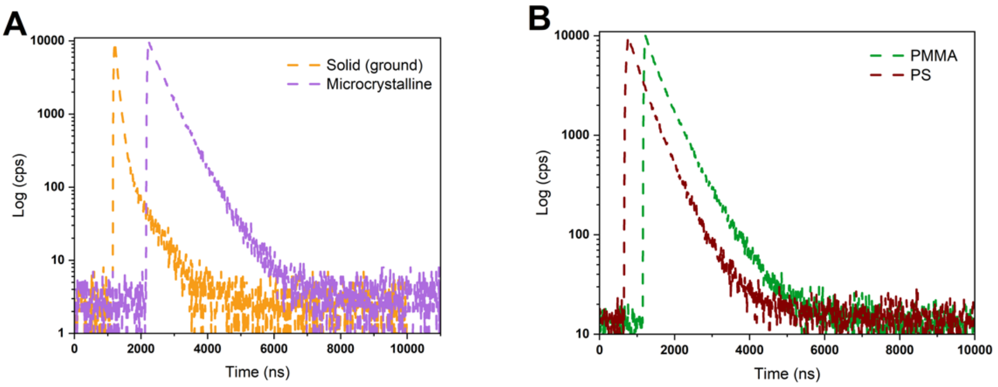

Figure S34. Emission decays of compound **4** at room temperature in solid state (ground; orange), microcrystalline state (purple), PMMA matrix (green) and PS matrix (brown).

Table S6. Selected photophysical data of compound **4** at RT.

	Medium	λ_{max} (nm)	τ (μs)[a]	φ	k_r (10^5 s^{-1})[b]
**4**	Solid(ground)	700	0.09 (94.6)/ 0.49 (5.4)	0.11	9.6
	Microcrystalline	610	0.39 (97.5)/ 0.72 (2.5)	0.75	19
	PMMA	630	0.27 (43.4)/ 0.59 (56.6)	0.30	6.6
	PS	650	0.28 (56.2)/ 0.53 (43.8)	0.34	8.7
	THF	785	n.d.	n.d.	-

(a) For lifetimes fitted with two exponentials, the pre-exponential factors B are given in parentheses. (b) k_r was calculated using amplitude-weighted averaged lifetimes

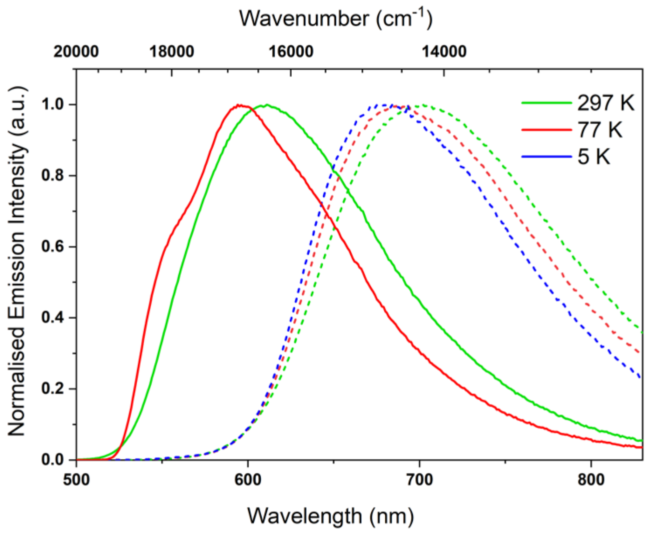

Figure S35. Normalized emission spectra of compound **4** in solid state (ground; short-dash lines), and microcrystalline state (solid lines) at various temperature. Green: 297 K, red: 77 K and blue: 5 K.

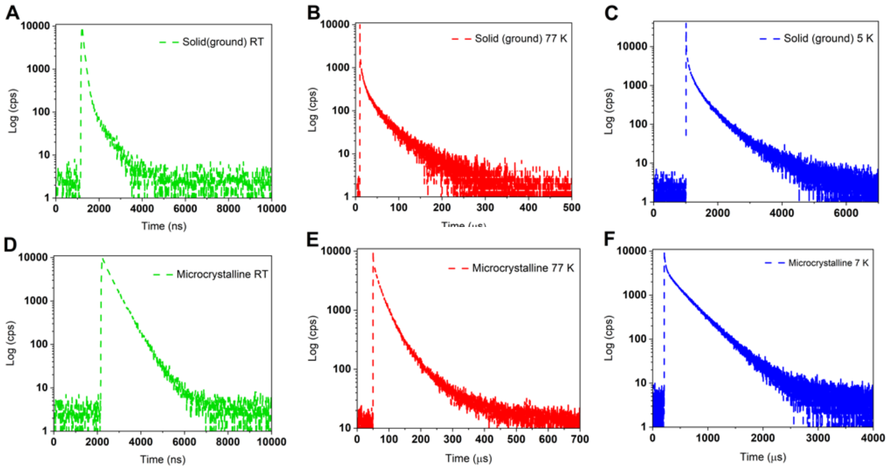

Figure S36. Emission decays of compound **4** at various temperature in solid state (ground; **A** (RT); **B** (77 K); **C** (5 K)), and microcrystalline state (**D** (RT); **E** (77 K); **F** (7 K)).

Table S7. Selected photophysical data of compound **4** at various temperature.

	Medium	λ_{max} (nm)	τ (µs)[a]	φ	k_r (10^5 s^{-1})[b]
**4**	Solid(ground; RT)	700	0.09 (94.6)/ 0.49 (5.4)	0.11	9.6
	Solid(ground; 77 K)	690	2.6 (52.6)/ 13 (35.9)/ 52 (11.5)	0.18	0.15
	Solid(ground; 5 K)	680	33 (35.0)/ 109 (33.9)/ 329 (25.7)/ 841(5.4)	-	-
	Microcrystal(RT)	610	0.39 (97.5)/ 0.72 (2.5)	0.75	19
	Microcrystal(77 K)	595	9.0 (30.1)/ 29 (62.3)/ 89 (7.6)	0.95	0.34
	Microcrystal(7 K)		38 (33.3)/ 297 (51.7)/ 594 (15.0)	-	

(a) For lifetimes fitted with two exponentials, the pre-exponential factors B are given in parentheses. (b) k_r was calculated using amplitude-weighted averaged lifetimes.

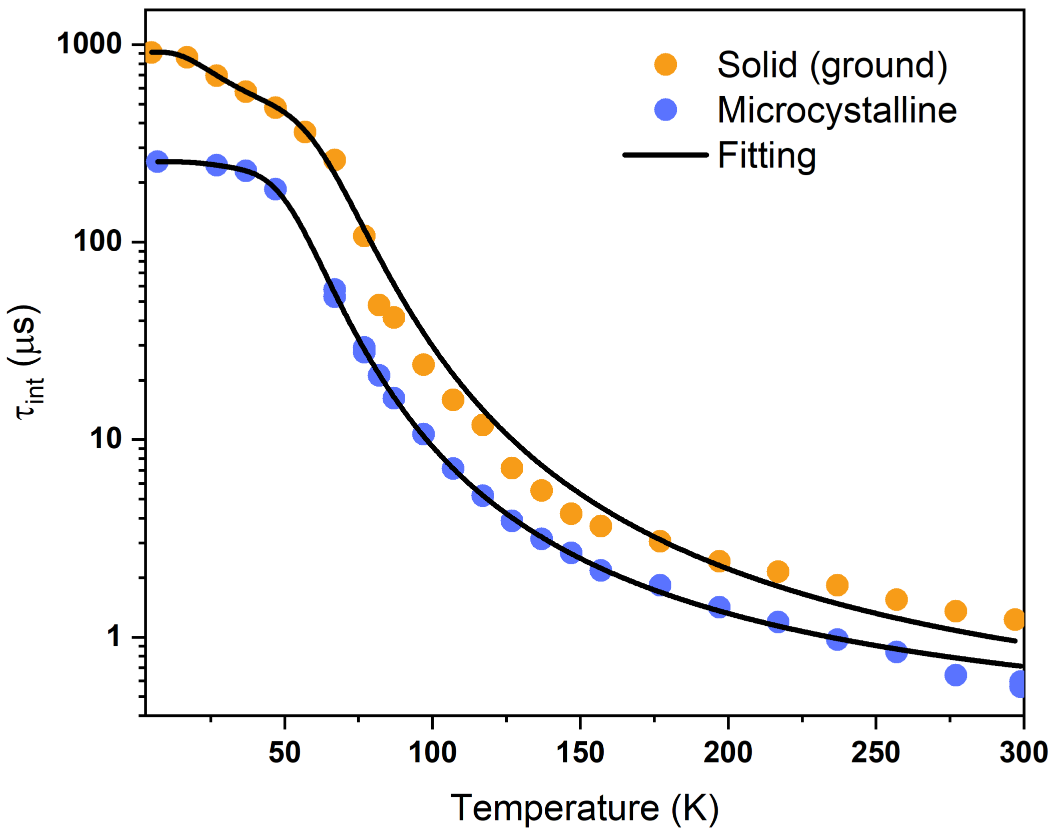

Figure S37. Variable temperature lifetimes of **4** in ground solid state (orange) and in microcrystalline state (blue).

5-exponential TADF fitting equation:

$$\tau_{\mathrm{r,av}}(T)=\frac{\left(2+e^{\frac{-\Delta E\left(T_1^{I}-T_1^{II,III}\right)}{k_BT}}+e^{\frac{-\Delta E\left(S_1-T_1^{II,III}\right)}{k_BT}}\right)}{\left(\frac{2}{\tau_r\left(T_1^{II,III}\right)}+\frac{e^{\frac{-\Delta E\left(T_1^{I}-T_1^{II,III}\right)}{k_BT}}}{\tau_r(T_1^{I})}+\frac{e^{\frac{-\Delta E\left(S_1-T_1^{II,III}\right)}{k_BT}}}{\tau_r(S_1)}\right)}$$

Table S8. Temperature dependent life-times details for compound **4** in solid state (ground).

Temp (K)	τ_1 (μs)	τ_2 (μs)	τ_3 (μs)	τ_2 (μs)	τ <amp> (μs)	τ <int> (μs)	χ^2	Φ%	τ <intrinsic>
5	32.97(35)	109.44(34)	329.02(26)	841.36(5)	178.75	395.03	1.08	19.6	912.03
7	28.27(28)	111.85(34)	292.17(31)	611.79(7)	180.18	321.03	1.08	19.6	919.31
17	35.18(24)	156.23(56)	348.66(20)		165.89	231.76	1.07	19.3	859.55
27	26.11(33)	134.63(49)	318.60(18)		131.91	208.26	1.05	19	694.29
37	15.70(29)	59.33(27)	159.62(34)	331.67(10)	107.84	191.40	1.04	18.7	576.71
47	16.71(35)	57.01(29)	149.63(29)	309.55(7)	88.15	164.36	1.04	18.4	479.11
57	14.26(32)	41.11(35)	110.12(26)	244.36(7)	65.04	124.89	1.01	18.1	359.36
67	15.84(40)	35.08(31)	82.26(23)	181.26(6)	46.34	83.13	1.03	17.8	260.37
77	2.61(53)	13.34(36)	51.69(11)		12.11	30.95	0.97	17.6	68.84
87	1.86(54)	8.55(36)	28.45(10)		7.03	15.99	0.93	16.9	41.62
97	1.14(52)	4.87(38)	15.07(10)		4.01	8.30	1.01	16.8	23.88
107	0.87(52)	3.36(39)	9.73(9)		2.61	4.96	1.09	16.5	15.86
117	0.65(45)	2.25(45)	5.93(10)		1.92	3.20	1.08	16.2	11.86
127	0.30(40)	1.24(47)	3.47(13)		1.14	1.98	1.12	15.8	7.21
137	0.23(37)	0.91(50)	2.44(13)		0.85	1.40	1.10	15.5	5.52
147	0.17(37)	0.69(50)	1.72(13)		0.63	1.01	1.08	15.1	4.21
157	0.17(40)	0.64(50)	1.52(10)		0.53	0.81	1.13	14.6	3.65
177	0.18(43)	0.54(49)	1.08(8)		0.42	0.58	1.20	13.9	3.06
197	0.14(41)	0.39(50)	0.78(9)		0.31	0.42	1.02	13.2	2.41
217	0.18(72)	0.49(28)			0.27	0.34	1.25	12.6	2.15
237	0.17(82)	0.48(18)			0.22	0.28	1.13	12.3	1.82
257	0.15(88)	0.46(12)			0.18	0.24	1.07	11.9	1.55
277	0.12(91)	0.45(9)			0.15	0.21	1.36	11.4	1.35
297	0.11(93)	0.47(7)			0.13	0.19	1.16	11	1.22

τ<amp>: amplitude-weighted averaged lifetimes; τ<int>: intensity-weighted averaged lifetimes. A linear increase of Φ% was found upon decreasing the temperature from 297 K to 77 K. At temperature <77 K, Φ% were estimated by assuming this linear relationship.

Table S9. Temperature dependent life-times details for compound **4** in microcrystalline state.

Temp (K)	τ_1 (μs)	τ_2 (μs)	τ_3 (μs)	τ <amp> (μs)	τ <int> (μs)	χ^2	Φ%	τ <intrinsic>
7	38.39(33)	296.96(52)	593.68(15)	255.35	387.56	1.13	100	255.35
27	73.70(21)	258.70(70)	498.49(9)	242.62	292.71	1.03	100	242.62
37	56.61(19)	231.73(69)	445.99(12)	224.80	275.08	1.10	98.89	227.32
47	59.87(21)	191.87(72)	411.78(7)	179.70	218.40	1.10	96.29	186.62
67	23.46(22)	52.05(74)	190.28(4)	50.75	67.91	1.18	95.02	53.40
77	9.01(30)	29.46(62)	88.71(8)	27.82	41.82	1.05	94.31	29.49
87	8.88(63)	25.81(37)		15.12	19.53	1.16	93.60	16.15
97	6.68(69)	16.83(31)		9.80	12.03	1.03	92.18	10.63
107	4.20(62)	10.18(38)		6.45	7.75	1.00	90.75	7.10
117	3.11(58)	6.83(42)		4.69	5.41	1.02	90.42	5.18
127	2.16(46)	4.58(54)		3.48	3.90	1.04	90.09	3.86
137	1.50(33)	3.29(67)		2.69	2.95	1.02	85.88	3.13
147	1.18(28)	2.56(72)		2.17	2.35	1.00	81.68	2.65
157	0.67(19)	2.02(81)		1.77	1.93	1.02	81.44	2.17
177	1.44(100)			1.44	1.44	0.96	78.06	1.84
197	0.27(12)	1.11(88)		1.01	1.08	0.94	76.38	1.32
217	0.23(15)	0.90(85)		0.81	0.88	0.92	73.69	1.09
237	0.10(15)	0.72(85)		0.63	0.71	1.06	73.29	0.85
257	0.14(13)	0.62(87)		0.56	0.61	0.99	72.90	0.76
277	0.14(21)	0.56(79)		0.47	0.53	1.04	71.76	0.65
297	0.20(33)	0.52(67)		0.41	0.47	0.91	74.72	0.54

τ<amp>: amplitude-weighted averaged lifetimes; τ<int>: intensity-weighted averaged lifetimes. A linear increase of Φ% was found upon decreasing the temperature from 297 K to 77 K. At temperature <77 K, Φ% were estimated by assuming this linear relationship.

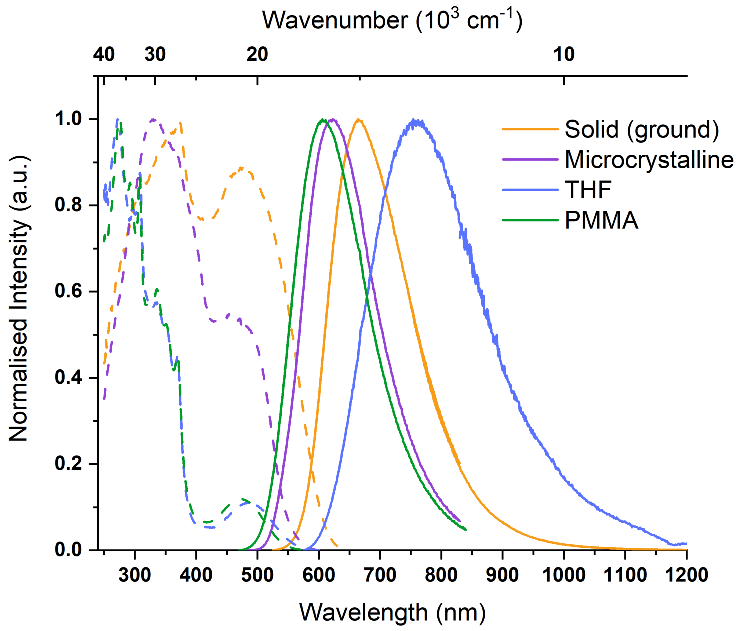

Figure S38. Normalized excitation and emission spectra of compound **5** in solid state (orange), microcrystalline state (purple), PMMA matrix (green; 1 wt% of the compound in DCM), and THF (blue) at room temperature. Solid lines for emission spectra and dash lines for excitation spectra.

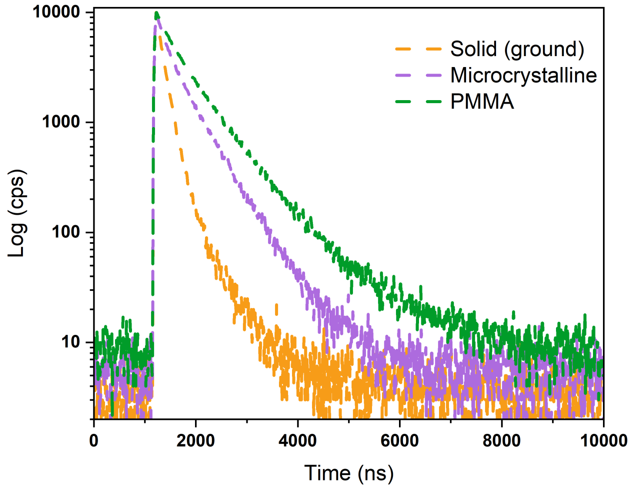

Figure S39. Emission decays of compound **5** at room temperature in solid state (ground; orange), microcrystalline state (purple), and PMMA matrix (green).

Table S10. Selected photophysical data of compound **5** at RT.

	Medium	λ_{max} (nm)	τ (μs)[a]	φ	k_r (10^5 s^{-1})[b]
**5**	Solid(ground)	665	0.15 (92.4)/ 0.48 (7.6)	0.15	8.4
	Microcrystalline	620	0.18 (39.5)/ 0.50 (60.5)	0.51	14
	PMMA	610	0.35 (52.2)/ 0.80 (47.8)	0.33	5.8
	THF	755	n.d.	n.d.	-

(a) For lifetimes fitted with two exponentials, the pre-exponential factors B are given in parentheses. (b) k_r was calculated using amplitude-weighted averaged lifetime.

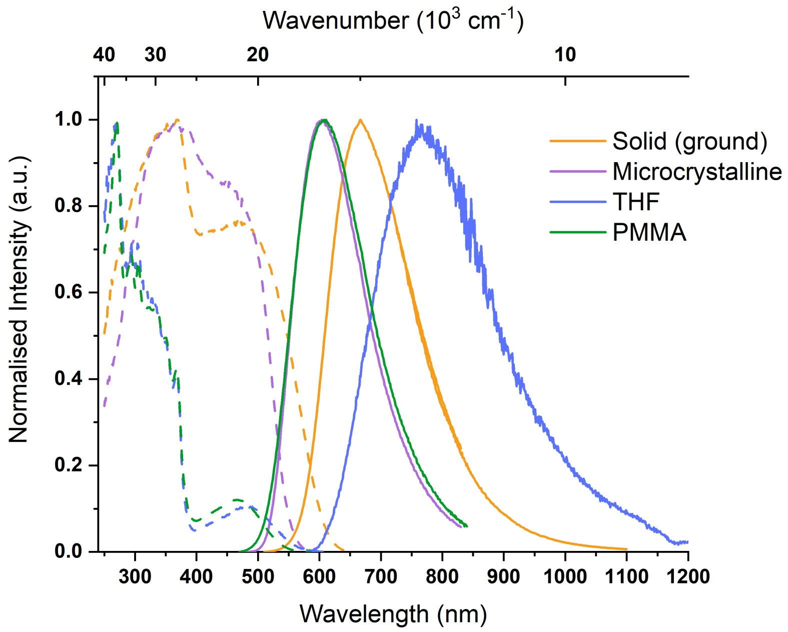

Figure S40. Normalized excitation and emission spectra of compound **6** in solid state (orange), microcrystalline state (purple), PMMA matrix (green; 1 wt% of the compound in DCM), and THF (blue) at room temperature. Solid lines for emission spectra and dash lines for excitation spectra.

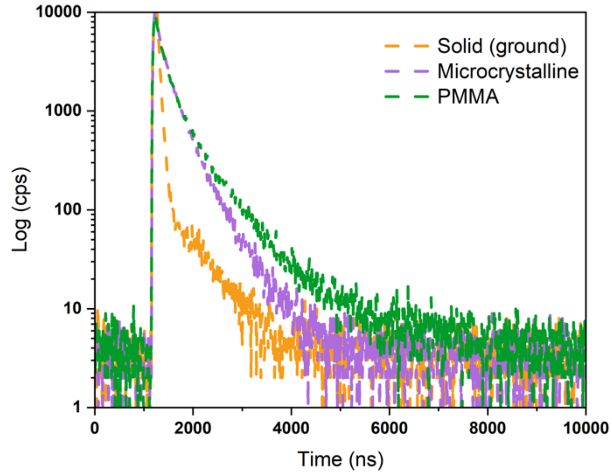

Figure S41. Emission decays of compound **6** at room temperature in solid state (ground; orange), microcrystalline state (purple), and PMMA matrix (green).

Table S11. Selected photophysical data of compound **6** at RT.

	Medium	λ_{max} (nm)	τ (μs)^{a}	φ	k_r (10^5 s^{-1})^{b}
**6**	Solid(ground)	665	0.034 (83.2)/ 0.078 (16.8)	0.03	8
	Microcrystalline	605	0.17 (70.5)/ 0.45 (29.5)	0.29	11
	PMMA	610	0.12 (64.4)/ 0.40 (30.9)/ 0.96 (4.6)	0.11	4.3
	THF	765	n.d.	n.d.	-

(a) For lifetimes fitted with two or multi exponentials, the pre-exponential factors B are given in parentheses. (b) k_r was calculated using amplitude-weighted averaged lifetime.

9. TD-DFT calculations

DFT and TD-DFT calculations were performed with the ORCA 6.0.1 program suite with tight SCF convergence criteria.[7] Geometry optimizations (gas-phase) omitting the respective anions were carried out with the BP86 functional[8] as implemented in ORCA, and a frequency analysis ensuring that the optimized structures correspond to energy minima. The def2-SVP[9] basis set was used for all atoms together with the auxiliary basis set SARC/J[10-14] in order to accelerate the computations within the framework of RI approximation. Relativistic effects were accounted for by employing the ZORA method as implemented for the def2-SVP basis set in Orca, and by employing the SARC-ZORA-TZVP[15,16] basis set for the metal atoms. Van der Waals interactions have been considered by an empirical dispersion correction (Grimme-D3BJ).[17,18] TD-DFT calculations for the first 20 singlet and triplet excited states were performed with the same basis sets, but the PBE0 functional[19,20] was used. Representations of electronic transition differences at isovalues of 0.0005 were produced with orca_plot as provided by ORCA 6.0.1 and with Chimera.[21]

Table S12. TD-DFT calculated vertical electronic transitions from the DFT optimized ground state S_0 of **3** to the first 20 singlet and triplet excited states.

Transition			Energy (eV)	Energy (cm^{-1})	Wavelength (nm)	Oscillator strength f
0-1A	->	1-1A	2,501	20172	495,7	0,1206
0-1A	->	2-1A	2,767	22317	448,1	0,0069
0-1A	->	3-1A	3,244	26164	382,2	0,0000
0-1A	->	4-1A	3,517	28366	352,5	0,0343
0-1A	->	5-1A	3,736	30132	331,9	0,0532
0-1A	->	6-1A	3,785	30526	327,6	0,0087
0-1A	->	7-1A	3,817	30783	324,9	0,0465
0-1A	->	8-1A	3,825	30848	324,2	0,0019
0-1A	->	9-1A	3,848	31039	322,2	0,0172
0-1A	->	10-1A	3,858	31117	321,4	0,0046
0-1A	->	11-1A	3,900	31458	317,9	0,0024
0-1A	->	12-1A	3,997	32241	310,2	0,0778
0-1A	->	13-1A	4,024	32457	308,1	0,0156
0-1A	->	14-1A	4,049	32661	306,2	0,0479
0-1A	->	15-1A	4,145	33435	299,1	0,0024
0-1A	->	16-1A	4,219	34030	293,9	0,0255
0-1A	->	17-1A	4,291	34609	288,9	0,0066
0-1A	->	18-1A	4,296	34648	288,6	0,0083
0-1A	->	19-1A	4,359	35158	284,4	0,0331
0-1A	->	20-1A	4,383	35349	282,9	0,0015
0-1A	->	1-3A	2,313	18655	536,0	
0-1A	->	2-3A	2,504	20195	495,2	
0-1A	->	3-3A	2,896	23360	428,1	

0-1A	->	4-3A	3,058	24667	405,4
0-1A	->	5-3A	3,097	24980	400,3
0-1A	->	6-3A	3,223	25998	384,6
0-1A	->	7-3A	3,432	27681	361,3
0-1A	->	8-3A	3,535	28512	350,7
0-1A	->	9-3A	3,599	29024	344,5
0-1A	->	10-3A	3,640	29360	340,6
0-1A	->	11-3A	3,651	29448	339,6
0-1A	->	12-3A	3,682	29697	336,7
0-1A	->	13-3A	3,723	30032	333,0
0-1A	->	14-3A	3,776	30457	328,3
0-1A	->	15-3A	3,833	30918	323,4
0-1A	->	16-3A	3,840	30972	322,9
0-1A	->	17-3A	3,860	31132	321,2
0-1A	->	18-3A	3,878	31277	319,7
0-1A	->	19-3A	3,929	31689	315,6
0-1A	->	20-3A	3,953	31880	313,7

Table S13. TD-DFT calculated vertical electronic transitions from the ground state S_0 at the optimized geometry of the T_1 state of **3** to the first 10 singlet and triplet excited states.

Transition			Energy (eV)	Energy (cm^{-1})	Wavelength (nm)	Oscillator strength f
0-1A	->	1-1A	1,987	16029	623,9	0,0768
0-1A	->	2-1A	2,476	19970	500,7	0,0176
0-1A	->	3-1A	2,909	23464	426,2	0,0000
0-1A	->	4-1A	3,171	25579	390,9	0,0274
0-1A	->	5-1A	3,367	27160	368,2	0,0442
0-1A	->	6-1A	3,404	27455	364,2	0,0122
0-1A	->	7-1A	3,430	27664	361,5	0,0069
0-1A	->	8-1A	3,463	27930	358,0	0,0083
0-1A	->	9-1A	3,634	29308	341,2	0,0151
0-1A	->	10-1A	3,681	29691	336,8	0,0668
0-1A	->	1-3A	1,817	14651	682,5	
0-1A	->	2-3A	2,220	17907	558,4	
0-1A	->	3-3A	2,553	20588	485,7	
0-1A	->	4-3A	2,768	22322	448,0	
0-1A	->	5-3A	2,898	23376	427,8	
0-1A	->	6-3A	3,029	24433	409,3	
0-1A	->	7-3A	3,130	25245	396,1	
0-1A	->	8-3A	3,248	26200	381,7	
0-1A	->	9-3A	3,275	26418	378,5	
0-1A	->	10-3A	3,300	26614	375,7	

Table S14. Cartesian coordinates of the DFT optimized ground state S_0 of **3**.

H	7.86034631205626	7.23519266045784	5.42670558891934
H	8.00313581358260	7.30124333431664	7.18989668633136
H	8.28189743103092	9.78165764916218	5.49039474387149
C	8.57300887754608	7.15885862583025	6.26028130893714
H	9.17785442402264	4.17123638677255	3.56666744095984
H	8.67164007351788	9.79963346297196	7.23197839790554
H	11.63253966604904	12.85583202627024	12.05289476071920
H	8.98920964624673	6.14416219293804	6.26359100382394
C	9.08545288821466	9.63428505893813	6.22672795133283
H	9.60612352253227	2.93856026930586	5.69109768923050
H	11.19844878836990	10.67851165245905	10.93521322653719
C	11.76016918453593	12.77092859910754	10.97273879742857
C	9.97529598467791	4.49344211987415	4.23733002939551
C	9.67278908584251	8.21690116573707	6.10745535229246
C	11.51377140803391	11.53687569701933	10.33937899321410
H	9.43848150398078	7.67899119904652	8.74229894515114
C	10.21297755726397	3.80524269331112	5.42615796708536
H	9.85593202544405	10.40266264126717	6.05375995923476
H	12.34692422430703	14.83746321038038	10.72834495887916
H	10.09157957129498	8.11185770749539	5.09804172963549
C	12.16044683560697	13.88281848388313	10.23238791059063
H	10.54582031093029	6.14280123824232	2.97557378670470
C	10.74938364532596	5.60648042901250	3.90071622613891
H	10.83534445721169	10.26547551311005	0.39656940013612
C	11.66249958121468	11.38949654080317	8.96336417319160
C	10.48205521573947	7.80694385476696	8.45562206324712
H	11.46616934965241	10.43299263081423	8.47781699583165
H	11.28015606055900	7.91365076705683	-0.10260503966685
C	10.79600221630941	8.04815390358761	7.11535639535002
C	11.22942290700134	4.24039367740570	6.28034875713043
C	12.32154640822630	13.75693285631093	8.84714998404534
C	11.26377056261362	9.63213628198135	1.17462846454185
H	11.42074541490869	3.71784144541178	7.21808667519428
C	11.51398838398351	8.29185319886929	0.89447359722615
C	12.07383791910255	12.50087890356767	8.21719141194956
H	11.24751970107313	12.22892601549704	1.84010239730155
C	11.78080855183648	6.03808702609060	4.73901748763505
H	11.20997061675977	7.54557454570383	10.46848741590617
C	11.73964194620590	10.21578305840728	2.35085250163499
H	12.30777486774007	5.54158130947973	0.75403843030806
H	13.11757862599710	16.60238210392280	8.65349994526706
C	11.47503577044130	7.74132173540644	9.42866133868979
C	12.00882661996642	5.34241402973746	5.93724246892447
C	12.71761246087567	14.65454186244101	7.78916669559284
C	12.22239299845532	7.45607697517286	1.77459381999582
C	12.10999160451504	9.30486477300677	3.35787335724289
C	12.31468468031981	7.93461009153206	3.08760654146246
C	12.02409333709669	11.68851380630680	2.39842076440007
N	12.29917210802255	12.57548759652295	6.84828177316578
C	13.09146278377812	16.00256388775809	7.74157980960842
C	12.15849772873191	8.20873787427801	6.77264113243467
Cu	12.23389621291362	11.11659014391373	5.74404926803590
C	12.27165147181481	9.56623207894535	4.77173450098435
C	12.98555311425762	6.28652759022899	1.19779270783496
N	12.46406422953652	8.38095291681734	5.37091931655871
C	12.66882834962227	7.22661278016277	4.38887875376988
H	11.99871568686645	12.05077032232394	3.43868589685984
C	12.68762817791606	13.88059742017869	6.58782466410116
H	12.80898704221607	5.65877249597379	6.60484337875470
H	13.31026646068132	12.91076714045334	1.09041512633151
H	13.21485743439799	11.22781849192365	-0.90039578497446
H	13.53701852177777	5.77760702476634	1.99546645830615
H	13.57081395290643	6.56512682582706	-0.92072320740646
H	13.45331035087049	8.88991997171309	-1.64050461267153
C	12.79850731648251	7.97705918369096	9.07862862011242
C	13.44949117576095	12.05452450451745	1.76472479703671
C	13.43210540608583	16.57039375891079	6.51474696391446
C	13.73696610636813	10.51621568180339	-0.25674664369461
C	13.16724080672705	8.23225314982628	7.75204058309052
C	13.87662598135059	9.18918031054027	-0.67864722345944
H	13.72438853335823	17.62009865532197	6.46373963481129
C	14.01441758088982	6.74974875815185	0.06795828829332
C	13.03432313730182	14.45759792837047	5.35930410035977
H	13.85471771633121	4.70976921397402	4.30587601711501
C	14.08591912325361	10.88854271079770	1.04871165044294
H	13.56631779600016	7.99438344398712	9.85258401856438
C	13.40278954486610	15.80057857693632	5.33553623469678
C	14.38278603530321	8.21145561638582	0.18794682392133

H	14.10562956422010	12.38643680681500	2.58194649380267
H	13.01438926675280	13.86465305108399	4.44275451821859
C	14.16330672479394	6.84549294839375	4.39347799871347
C	14.58817077095971	5.51406035854905	4.32633463862332
H	14.89957494249114	6.10129373206396	0.14900737980852
H	13.67441689328048	16.26545406362072	4.38613723327076
C	14.85974019416627	9.97573237248874	1.78018123743314
C	15.01764595602818	8.66335662050736	1.35043122448304
C	14.60154194574958	8.60975790347296	7.45418531776983
H	14.66605174472926	8.82095767086093	6.38283270381365
H	14.81433023235616	8.89558885812814	4.39573903316227
H	14.30799345316682	10.72659958423660	7.91603324218609
C	15.13141403056521	7.85419637183162	4.39243622970288
H	15.24424437865311	10.27237444148117	2.75799734355843
H	15.52133453774857	7.94513313875848	1.99551428549690
H	15.31557527616941	6.55840026909367	7.19383330614436
C	14.98456315283332	9.90487180081780	8.18784533833297
H	14.94049651712528	9.78465666031345	9.27969767021470
C	15.94970244866400	5.20249214401007	4.28907572945914
C	15.56993884994814	7.46227861576241	7.76546400816988
H	15.55186693056456	7.20699020046084	8.83530644773628
H	16.25926728721277	4.15773359204888	4.24146667119253
C	16.48969644426511	7.54824428923762	4.37283851796831
H	16.01003669734664	10.20024977834936	7.92103657466759
H	16.59932918654511	7.74660381296161	7.50522562826092
C	16.90618339785048	6.21669018466961	4.32364563388629
H	17.22401005093061	8.35452356700100	4.38508029592933
H	17.96890020027416	5.97303569778910	4.30822275423748

Table S15. Cartesian coordinates of the DFT optimized triplet excited state T_1 of **3**.

H	7.99274199111245	6.96825135266943	5.07531247099029
H	7.93176369635280	7.17282795940178	6.83398671569057
H	8.17838574033467	9.54882151752008	5.01432859759122
C	8.61141805092659	7.01822624769210	5.98278028417044
H	9.58989945699102	3.84073774830434	3.85836971855408
H	8.38760707102844	9.70540195496756	6.78004449669059
H	11.55591307857114	12.67429883371987	12.00403087709790
H	9.10864031894242	6.04930060804685	6.11092315342238
C	8.91372351388804	9.51858770462084	5.83147716647900
H	10.20335146123134	2.78941935339936	6.03567210721862
H	10.65613463783684	10.74265999353864	10.73599159354074
C	11.68194134363643	12.63706903530991	10.92132450865387
C	10.36185662898149	4.28840683972086	4.48602855654521
C	9.63097266278060	8.15833504061598	5.85938462449735
C	11.16810607467074	11.54513601337399	10.20518359126190
H	9.19193527940345	7.76020236035712	8.48833680731248
C	10.70283223677523	3.70066340210285	5.70369545183164
H	9.63329073963891	10.33741402854041	5.66933193900803
H	12.76104717547115	14.51754789715607	10.84423059111560
H	10.15161244293043	8.04347956167257	4.90045828360849
C	12.36237390220175	13.67911766733806	10.27119017207300
H	10.69681901382889	5.92889040692122	3.12902690138626
C	10.99195843123184	5.46301235657281	4.06754902279141
H	10.55115707502315	10.11025986026276	0.35863786006187
C	11.31522805447607	11.45977246804612	8.82131604293866
C	10.24917498265435	7.93588759295001	8.28694535463383
H	10.94032043100032	10.60142901064947	8.26229470910197
H	11.00969144824517	7.72553518695347	-0.04025419457740
C	10.66836444079486	8.13311021386862	6.96706801181702
C	11.68924577559501	4.29333346138425	6.49670198488592
C	12.51998309673795	13.61280704185005	8.89138116423551
C	11.00452391777197	9.51913429571289	1.15723819051824
H	11.96622393630983	3.84974756731472	7.45387675642220
C	11.26439528078122	8.15925848550029	0.92802726090911
C	11.98881604187614	12.50113240668904	8.17384073458157
H	11.02755593414098	12.12261146386016	1.57429056015717
C	11.99084469204423	6.05648965315752	4.84422726600406
H	10.81277626490192	7.76864518285412	10.36325980080774
C	11.49289755207775	10.16683839345819	2.28224041398403
H	12.15232449292080	5.49998968603351	0.79470713052700
H	14.00458241599054	16.19268062714858	8.87038277941811
C	11.15686394805574	7.94588197345840	9.34284707713342
C	12.33335229515515	5.44912273997304	6.06330454255404
C	13.16141323328902	14.45330493067306	7.89396088419675
C	12.05021483314162	7.40224364725157	1.82503560051946
C	11.94826327975597	9.32522180300192	3.33647042828968
C	12.21487470719307	7.93744385674271	3.10208607510099

C	11.74392461780818	11.64763897637320	2.26009968442127
N	12.26344311810968	12.60681722477018	6.81798273055235
C	13.85127518469927	15.66036437256786	7.93044998474645
C	12.04812636254071	8.33507219485508	6.71938736659596
Cu	12.00140795332974	11.23968279412236	5.61724551366473
C	12.19500274567672	9.65355054302084	4.68601490340968
C	12.82620050929142	6.23467781494402	1.26216864616960
N	12.50281994446501	8.47926691567334	5.36582873577844
C	12.73174781890216	7.31873730977949	4.40779609922773
H	11.58435136988616	12.09780717483996	3.25726476734767
C	12.97642007731982	13.78253398508240	6.64826912052468
H	13.11860349035710	5.88515340717397	6.67836389260594
H	13.16302378651118	12.91112306229627	1.15034294259115
H	12.89846995325143	11.13062060495341	-0.84433188966729
H	13.35966288977167	5.71453105566912	2.06372010142114
H	13.47587832197015	6.47617950064702	-0.83930334709540
H	13.16376215266942	8.78208771036098	-1.54143557356227
C	12.49759922645661	8.21199179224856	9.09275938385130
C	13.22316940796436	12.01189031507877	1.78132255108999
C	14.35242735523500	16.18737018280283	6.73043727217379
C	13.47577130009307	10.44987967723029	-0.21470258242029
C	12.96372401577022	8.42887617837891	7.78992378920270
C	13.62503268221279	9.11695525087844	-0.60959098428044
H	14.89676219412175	17.13221694082745	6.74584976707814
C	13.88146475891576	6.69980880373392	0.15773408155317
C	13.47916477181901	14.30768113744574	5.45263728880477
H	14.10933434367758	4.92361095294283	4.06298587242637
C	13.87871430890788	10.85867893484359	1.06360210968697
H	13.20012616554069	8.26948976137013	9.92520693566569
C	14.16751771351253	15.51959227256812	5.50914695322464
C	14.20509721114111	8.17211837008651	0.25072733867988
H	13.82424195924584	12.26771089664732	2.66609167362417
H	13.33525646137414	13.77580193778131	4.51094937749361
C	14.25017318782521	7.05832693092275	4.34816535829813
C	14.77943193936825	5.77923051719144	4.14348924567801
H	14.78559399059920	6.08588723928197	0.28628532335051
H	14.57112250035227	15.95277694885148	4.59363654761827
C	14.70474099259065	9.98250821478669	1.78149114145621
C	14.87958827487151	8.66256068747570	1.37220524010473
C	14.42053797601577	8.78471951814891	7.57772908137202
H	14.52029765894696	9.08569422580007	6.52980966470602
H	14.73017314337054	9.13646559690738	4.58004553664695
H	14.18485421390025	10.84297327396330	8.27988105917513
C	15.13704880415340	8.13471886899717	4.46020548621207
H	15.11406199594201	10.30502711855948	2.73988510198887
H	15.42951156546596	7.97144817995800	2.00853419656696
H	15.10613921017899	6.75564960406099	7.11696156752454
C	14.84428232586264	9.98026315426440	8.44344010617629
H	14.82172758153856	9.73967541588945	9.51655896212363
C	16.16006330851079	5.58124942518462	4.06336628633642
C	15.34055126529659	7.57732270265528	7.80757806614062
H	15.24610214997478	7.20065136182079	8.83752563083988
H	16.55136584651940	4.57446371160040	3.90966880035611
C	16.51488387651273	7.94135742665599	4.39479156954004
H	15.87218347719201	10.28127915241406	8.19308813948954
H	16.39067489102944	7.85614242558238	7.63920492423379
C	17.03483461478728	6.66051476210615	4.19666749188223
H	17.18532845754099	8.79690613891715	4.48916779851208
H	18.11315950040309	6.50478991365039	4.14596910133128

10. OLED Fabrication

ITO-covered glass substrates (22·22 mm², 70 to 100 Ω resistivity) were purchased from SPI Supplies®. The ITO layer was pre-patterned by a standard photolithographic procedure. Prior to organic layer deposition the patterned substrates were cleaned by consecutive ultrasonication in double distilled water, acetone, and isopropanol (15 minutes each) and were dried in a nitrogen stream. Afterwards Poly(3,4-ethylenedioxythiophene) polystyrene sulfonate (PEDOT:PSS, Heraeus Clevios™ P VP AI 4083), utilized as a hole-injection layer, was spin-coated at 3000 rpm for 60 s, resulting in a film thickness of 40 nm. The PEDOT:PSS dispersion was filtered by a 0.2 µm nylon syringe filter (VWR®) for removal of unwanted impurities and agglomerates. An additional thermal annealing step on a hot plate at 130 °C for 30 minutes facilitates removal of parasitic water from the PEDOT:PSS layer. Directly afterwards, the substrates were transferred to a nitrogen glovebox system (Jacomex, oxygen < 1ppm, water < 0.1ppm). The emissive layer of the OLEDs consists of the matrix material 1,3-Bis(N-carbazolyl)benzene (mCP) doped with **4**. mCP (purchased from Sigma Aldrich at 97 % purity) was further purified by gradient sublimation. Mixtures of the matrix material and the emitter, with the Cu^{I} iPC complex representing a proportion of 5 wt%, were dissolved in dichloromethane (DCM, purchased from Sigma Aldrich at spectroscopic grade) at concentrations of 5 $mg \cdot mL^{-1}$. The solutions were spin-coated at 2000 rpm for 60 s resulting in an emissive layer thickness of 40 nm. Removal of parasitic solvent was carried out by thermal annealing on a hot plate at 70 °C for 45 minutes. Subsequently, the samples were transferred into a high vacuum deposition system (base pressure 10^{-9} mbar) and 50 nm of Tris(2,4,6-trimethyl-3-(pyridin-3-yl)phenyl)borane (3TPYMB, purchased from Ossila) were deposited as electron transport layer at a deposition rate of 2 to 4 $nm \cdot min^{-1}$. 3TPYMB was deposited from boron nitride crucibles (R D Mathis) by resistive heating and the film thickness was monitored by a quartz crystal microbalance. Before and after, the samples were exposed to ambient air (yellow light conditions) for a time span less than 10 minutes. Top contacts were deposited in a separate and glovebox-integrated high-vacuum deposition system (Leybold UNIVEX450, base pressure 10^{-7} mbar) to avoid cross contamination of the electron transport layer and to ensure inert conditions in between top contact deposition and device encapsulation. Layered cathode top contacts were fabricated by consecutive evaporation of 1 nm LiF (purchased from MaTecK GmbH at 99.9 % purity) at deposition rates of 0.1 $Å \cdot s^{-1}$ and 120 nm Al (purchased from Chempur at 99.999 % purity) at 2 to 10 $Å \cdot s^{-1}$. Both materials were deposited from commercial tungsten boat sources (Lesker) by resistive heating and the film thickness was monitored by a quartz crystal microbalance. The cathode structure was patterned by shadow masks, resulting in a circular active area of approximately 3 mm^2. Uniform top contacts were ensured by substrate rotation (16 rpm) during the deposition process. Finally, the devices were encapsulated with microscope slides and a two-component epoxy resin (Loctite EA9492), which was cured overnight before ejecting the device from the glovebox system.

***J-V-EL* Characteristics, Spectral Emission and Transient PL.** Current-voltage measurements were carried out using source measurement units of a semiconductor parameter analyzer (Keysight B1500A). The sweep step size was 250 mV with a hold time of 200 ms for each data point. Electroluminescence spectra were recorded under constant voltage operation at a home-built confocal microscopy setup. Light was collected with a high numerical aperture immersion oil objective (Olympus UAPON,100 x TIRF, NA = 1.49) and spectrally analyzed by a spectrometer (Princeton Instruments, Acton SP2300) in combination with a CCD camera (Princeton Instruments, Pixis 400B). During the *I-V* sweeps the OLED emission was tracked with an avalanche photodetector (APD, Excelitas SPCM-AQRH-14, QE 65 % at 650 nm, dark counts < 100 $counts \cdot s^{-1}$). A dichroic mirror and a razor-edge filter were used for PL measurements. Time-correlated single photon counting to track the transient decay of **4** in mCP was carried out on an actual OLED beside the active pixel. A pulsed laser (λ_{ex} = 520 nm) and a hardware correlator (PicoHarp 300) synchronized by the electrical signal

of the laser, were utilized to record histograms. PL spectra were measured under cw-excitation with a 532 nm Laser (CNI).

External Quantum Efficiency. The OLED pixel was adjusted surface parallel to the APD detector (Excelitas SPCM-AQRH-14, QE 65 % at 650 nm, dark counts < 100 counts·s^{-1}) in 34 cm distance. In this case the detector area can be treated as point-like with respect to the pixel area. Proper orientation was ensured by an alignment laser. The electroluminescence was tracked during an *I*-*V* sweep (see above). We have calculated the EQE under assumption of Lambertian emission into the hemisphere with the integrated detector area ($2.5 \cdot 10^{-8}$ m^2) and a maximum APD detection efficiency of 65 %. The luminance was calculated together with the spectral data and the brightness sensitivity curve *V(λ)* as well as the photopic efficiency at 555 nm, according to the International Commission on Illumination (CIE).

Transient Electroluminescence. Transient EL was measured at a home-built setup. The OLED pixels were contacted with micromanipulators (FormFactor DPP210), shielded triaxial probe arms (FormFactor 100525) and tungsten needles (FormFactor PTT-120). A photomultiplier tube (PMT) in combination with a collection lens was used for EL detection. The EL signal of the PMT was amplified by a current amplifier (DHPCA-100, high speed mode, amplification: 10^3) and tracked via an oscilloscope (Tektronix TDS 3032) at 50 Ω input impedance. The OLED pixel was biased via a Hewlett Packard pulse generator. The voltage cycle was synchronized with the EL signal via the second oscilloscope input at 1 MΩ input impedance. Duty cycles of 15 V (2 ms, on-phase) and 0 V (8 ms / 98 ms, off-phase) were applied to the OLED. 500 duty cycles were averaged to yield the transient EL of the OLED. The data were corrected by the instrument response of the setup, recorded under the condition that the PMT is shielded from OLED emission. The current response was tracked via the 50 Ω input impedance of the oscilloscope in a second measurement. The transient voltage, current and electroluminescence response is exemplarily shown in Figure S44.

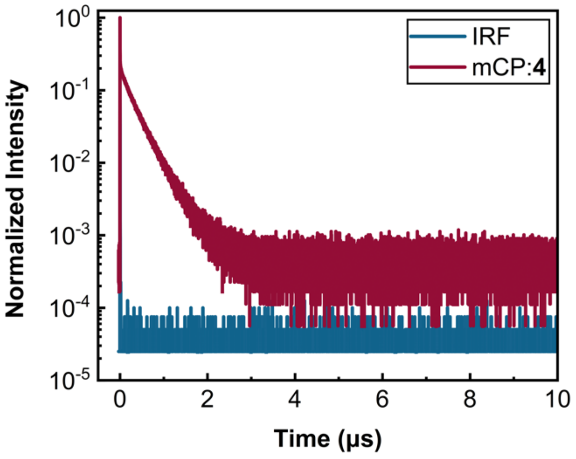

Figure S42. Transient PL decay of **4** (5 wt%) in mCP. The instrument response function (IRF) of the setup is shown for comparison. The decay was measured on an actual OLED beside the active pixel.

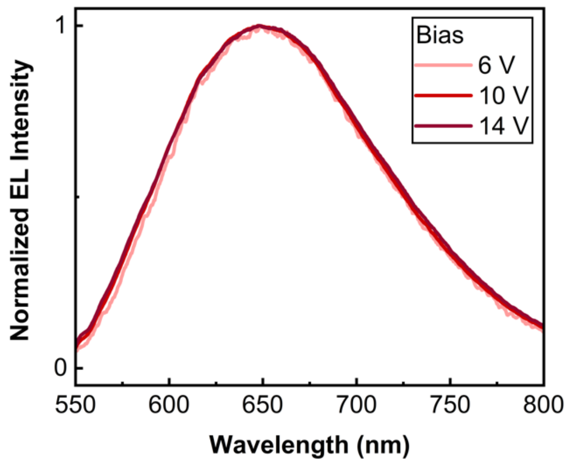

Figure S43. Normalized EL spectra measured under constant voltage operation at 6 V, 10 V and 14 V.

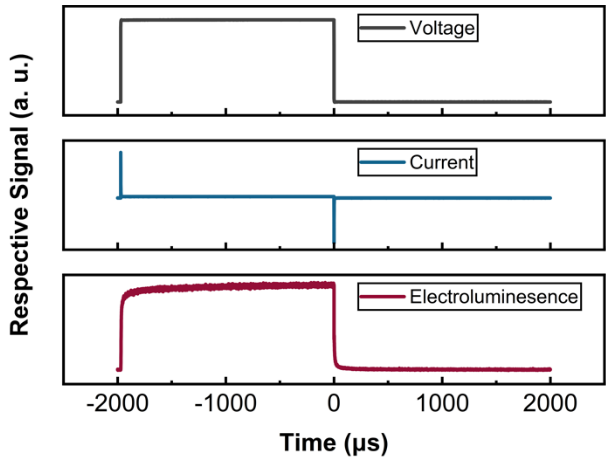

Figure S44. Transient response of a representative OLED pixel to a voltage (15 V / 0 V) duty cycle of 100 ms period length (displayed is an excerpt). The voltage, current and electroluminescence signals are shown from top to bottom. Each signal is averaged over 500 cycles.

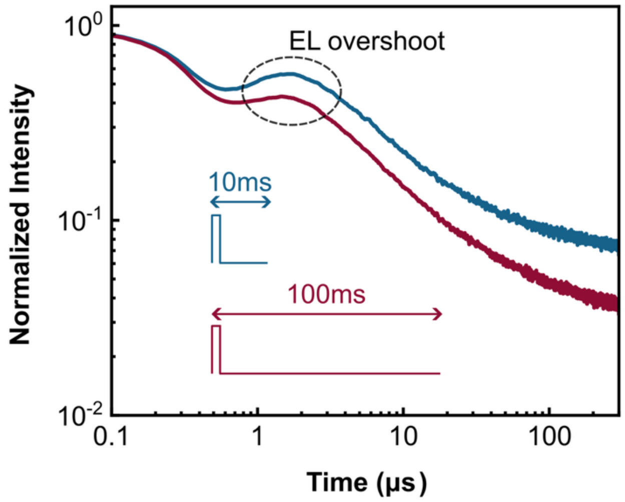

Figure S45. Normalized transient EL decay of a representative pixel with variation of the duty cycle length (10 ms and 100 ms). The overshoot in the EL signal 2 μs after switching-off the pixel refers to trapped and stored charge carriers which exhibit delayed recombination. The EL overshoot scales inversely with the duty cycle length as excess charge carriers are hardly removed during short off-periods. The EL signals are averaged over 500 cycles.

11. Mechanochromic TADF

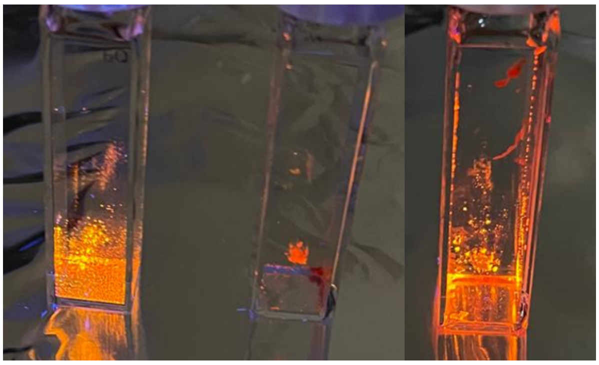

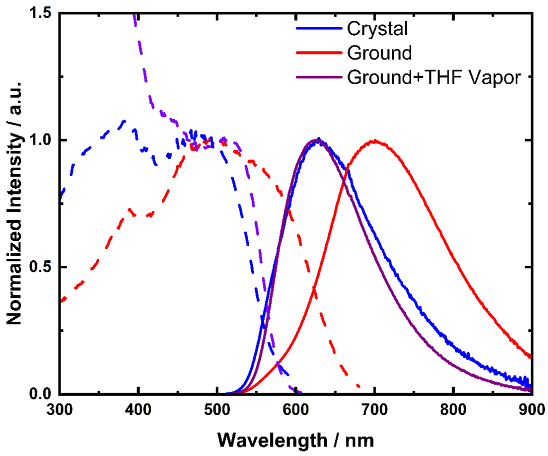

Figure S46. Left: Luminescence of [Cu(^{tbu}Cbz)(iPC)] irradiated with a UV lamp (365 nm) in the crystalline solid state, after grinding and upon THF vapor exposure. Right: Corresponding normalized excitation (dashed) and emission (solid) spectra of [Cu(^{tbu}Cbz)(iPC)].

After recording the emission spectrum in the crystalline state, the sample was ground evenly in the glovebox, causing the colour of the solid to change from orange to dark red (Figure S46). The emission spectrum recorded afterwards shows a bathochromic shift with a maximum at 700 nm. When the cuvette containing the ground sample is exposed to THF vapors, the sample exhibits reversible behavior after a short time and returns to an orange solid with an emission maximum at 630 nm.

Powder X-ray diffraction

Powder X-ray diffraction (PXRD) was performed on a Bruker D8 Advance in Bragg-Brentano geometry using Cu Kα radiation. Data were collected over a 2θ range of 4° to 50° with a step size of 0.02°. The samples were loaded on a sample holder and covered with a plastic dome in an argon-filled glovebox.

The crystal structure of **4**, determined by SC-XRD, exhibits a very large cell (unit-cell volume = 6852.8(4) Å^3), which results in a PXRD pattern with a high density of overlapping reflections. However, the simulated and experimental PXRD patterns show good overall agreement (Figure S47). Discrepancies between the experimental and simulated PXRD patterns may arise from the different data-collection temperatures (~100 K for SC-XRD and room temperature for PXRD). Although, the data do not allow for unequivocal assignment of the bulk microcrystalline material to the same phase as the single crystal, repeated SC-XRD measurements on multiple crystals from that sample batch consistently yielded the identical structure, supporting phase assignment.

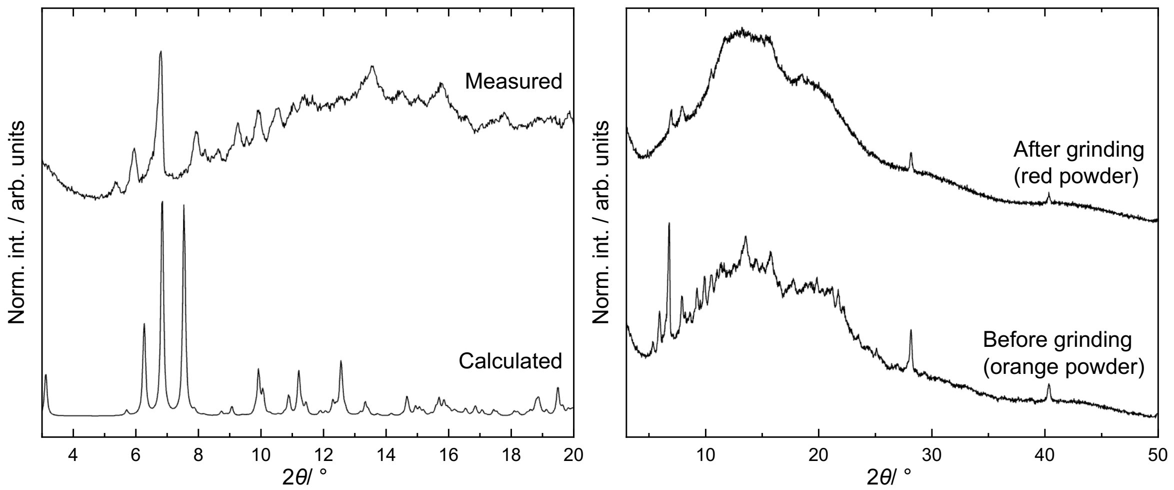

Figure S47. PXRD pattern of [Cu(^{tbu}Cbz)(iPC)]. Left: Calculated and measured PXRD pattern of the single crystal and the crystalline bulk, respectively. Right: PXRD pattern of the orange crystalline and the grounded phase.

After grinding microcrystalline **4**, most of the reflections in the PXRD pattern disappear. Only a few weak reflections remain, and the loss of the strong reflections characteristic of the microcrystalline phase indicates the ground solid is largely amorphous. Two rather sharp reflections at ~28° and ~40° 2θ can be attributed to potassium chloride, a side product of the complex synthesis.

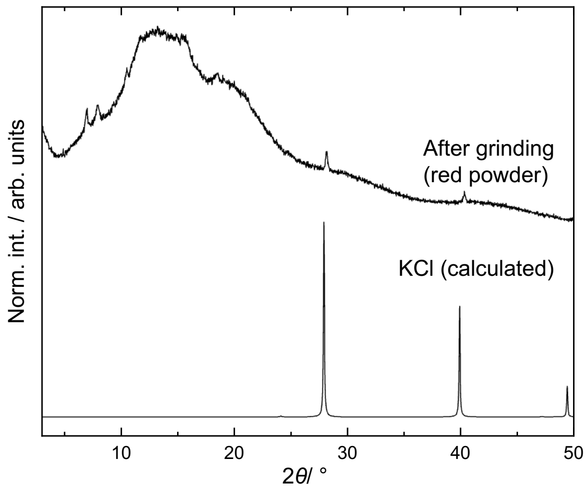

Figure S48. PXRD pattern of the ground [Cu(^{tbu}Cbz)(iPC)] in combination with the calculated PXRD pattern of potassium chloride (KCl).

12. References

[1] W. L. F. Armarego, *Purification of laboratory Chemicals*, Butterworth Heinemann, Oxford; Boston, **1996**.

[2] S. Maity, A. M. T. Muthig, I. Sen, O. Mrózek, A. Belyaev, B. Hupp, A. Steffen, *Angew. Chem. Int. Ed.* **2024**, e202409115.

[3] R. B. Bedford, M. Betham, *J. Org. Chem.* **2006**, *71*, 9403.

[4] G. M. Sheldrick, *Acta Crystallogr. C* **2015**, *71*, 3.

[5] O. V. Dolomanov, L. J. Bourhis, R. J. Gildea, J. A. K. Howard, H. Puschmann, *J. Appl. Crystallogr.* **2009**, *42*, 339.

[6] A. L. Spek, *Acta Cryst.* **2015**, *71*, 9.

[7] F. Neese, WIRES Comput. Molec. Sci., 2022 12(1)e1606.

[8] A. D. Becke, Phys. Rev. A. 38, 3098 (1988).

[9] F. Weigend and R. Ahlrichs, Phys. Chem. Chem. Phys. 7, 3297 (2005).

[10] F. Weigend, Phys. Chem. Chem. Phys. 8, 1057 (2006).

[11] D. A. Pantazis and F. Neese, J. Chem. Theory Comput. 5, 2229 (2009).

[12] D. A. Pantazis, X. Y. Chen, C. R. Landis and F. Neese, J. Chem. Theory Comput. 4, 908 (2008).

[13] D. A. Pantazis and F. Neese, Theor. Chem. Acc. 131, 1292 (2012).

[14] D. A. Pantazis and F. Neese, J. Chem. Theory Comput. 7, 677 (2011).

[15] J. D. Rolfes, F. Neese and D. A. Pantazis, J. Comput. Chem. 41, 1842 (2020).

[16] D. A. Pantazis, X. Y. Chen, C. R. Landis and F. Neese, J. Chem. Theory Comput. 4, 908 (2008).

[17] S. Grimme, J. Antony, S. Ehrlich and H. A. Krieg, J. Chem. Phys. 132, 154104 (2010).

[18] S. Grimme, S. Ehrlich and L. Goerigk, J. Comput. Chem. 32, 1456 (2011).

[19] J. P. Perdew, K. Burke and M. Ernzerhof, Phys. Rev. Lett. 77, 3865 (1996).

[20] C. Adamo and V. Barone, J. Chem. Phys. 110, 6158 (1999).

[21] E. F. Pettersen, T. D. Goddard, C. C. Huang, G. S. Couch, D. M. Greenblatt, E. C. Meng and T. E. Ferrin, J. Comput. Chem. 25, 1605 (2004).
